# Patterns and trends of eating disorders among women of childbearing age: a comprehensive analysis from 1990 to 2021 with future predictions

**DOI:** 10.1007/s40519-026-01842-8

**Published:** 2026-03-23

**Authors:** Qianhui Wen, Qian Wang

**Affiliations:** 1 Department of Pediatrics, West China Second University Hospital, Sichuan University, Chengdu, Sichuan China; 2 Key Laboratory of Birth Defects and Related Diseases of Women and Children (Sichuan University), Ministry of Education, Chengdu, Sichuan China

**Keywords:** Eating disorders, Women of childbearing age, Global Burden of Diseases, Disability-adjusted life years

## Abstract

**Purpose:**

Eating disorders (EDs) among women of childbearing age (WCBA; defined by the World Health Organization as women aged 15–49 years) have emerged as a significant public health concern on a global scale. This study aims to explore the patterns and trends of EDs—specifically anorexia nervosa (AN) and bulimia nervosa (BN), as defined by the Global Burden of Disease (GBD) 2021—among WCBA from 1990 to 2021.

**Methods:**

Using data from the GBD Study 2021, we quantified the disease burden using disability-adjusted life years (DALYs) and age-standardized DALY rates (ASDR). Temporal trends were assessed via the estimated annual percentage change (EAPC). Relationships between socioeconomic development and burden were evaluated using Spearman’s correlation with the Sociodemographic Index (SDI). Future trends were projected using a Bayesian Age-Period-Cohort (BAPC) model.

**Results:**

In 2021, the global burden of EDs in WCBA reached 2,035,736 DALYs (95% UI: 1,229,250–3,188,529). From 1990 to 2021, the global ASDR rose from 93.45 to 106.05 per 100,000 population, with an EAPC of 0.48 (95% CI 0.44–0.52). BN consistently imposed a higher burden than AN. ASDR was positively correlated with SDI (*r* = 0.808, *P* < 0.001), with high-SDI regions exhibiting the highest burden, while the middle-SDI quintile and the East Asian region showed the most rapid increases. By 2035, the global ASDR is projected to reach 109.84, with peak burdens shifting toward the 20–29 age group.

**Conclusions:**

The global burden of EDs among WCBA is substantial and increasing, particularly in rapidly urbanizing and high-income regions. Given the intersection of EDs with reproductive health, integrated screening within obstetric and gynecological care is urgently needed. Public health strategies should prioritize BN and regions undergoing rapid socioeconomic transition to mitigate this escalating crisis.

*Level of evidence* Level IV, evidence obtained from multiple time series (descriptive time-trend analysis).

**Supplementary Information:**

The online version contains supplementary material available at 10.1007/s40519-026-01842-8.

## Introduction

Eating disorders (EDs) in women of childbearing age (WCBA) have emerged as a critical global public health challenge. Over the past few decades, the incidence of EDs in this demographic has exhibited a significant upward trend, likely associated with heightened sociocultural scrutiny of body image, shifting nutritional patterns, and escalating psychosocial stressors [1].

Epidemiological data from Western nations indicate that ED prevalence in young adult women ranges from 5.5% to 17.9%, rates that markedly surpass those observed in males [2]. In Australia, the prevalence of EDs in young women reaches 22.2% [3], and similar rising patterns are increasingly evident in South Asia [4].

Beyond their psychological toll, EDs impose profound physiological burdens on WCBA, primarily by disrupting neuroendocrine regulation and reproductive health [5, 6]. Clinically, these disorders often co-occur with conditions such as polycystic ovary syndrome (PCOS) [7]. Notably, the perinatal period represents a particularly vulnerable window where hormonal fluctuations and psychological stressors may exacerbate ED symptoms, predisposing individuals to severe maternal complications [8].

Collectively, EDs account for over 3.3 million DALYs globally across all ages [9, 10]. However, despite these severe maternal and neonatal risks, current epidemiological insights are predominantly derived from high-income Western nations. This geographic disparity obscures the rising burden of EDs in low- and middle-income regions [11, 12], creating a blind spot in global health policy.

To address this gap, this study draws on data from the Global Burden of Disease (GBD) Study to quantify temporal trends in EDs among WCBA from 1990 to 2021, with projections through 2035. By elucidating spatiotemporal distributions and regional disparities in ED-related health loss, we aim to provide a robust evidence base for developing equitable, targeted intervention strategies.

## Methods

### Data source and disease definition

Data analyzed in this study were derived from the 2021 GBD study. GBD 2021 provides standardized estimates of the epidemiological burden for 371 diseases and injuries. The GBD geographic hierarchy organizes 204 countries and territories into 21 geographic regions based on geographical proximity and epidemiological similarity [13]. These locations encompass both sovereign states and non-sovereign entities, such as territories, protectorates, or autonomous regions. Furthermore, to analyze health patterns across different socioeconomic development stages, these locations are categorized into five Socio-demographic Index (SDI) quintiles (high, high-middle, middle, low-middle, and low). The SDI is a composite measure of per capita income, average education years, and total fertility rate, providing a basis for comparing health outcomes across varying levels of development [13]. In this framework, EDs are categorized into two primary types: AN and BN. Notably, other prevalent EDs, such as binge eating disorder (BED) and other specified feeding and eating disorders (OSFED), are excluded from current GBD estimates.

The GBD methodology incorporates cases meeting diagnostic criteria from the Diagnostic and Statistical Manual of Mental Disorders (DSM) or the International Classification of Diseases (ICD). For this analysis, WCBA were defined as females aged 15 to 49 years, consistent with World Health Organization guidelines [14]. Data were retrieved from the Global Health Data Exchange (GHDx) platform (https://ghdx.healthdata.org/gbd-2021/sources). To assess age-specific trends, DALYs were analyzed across seven stratified age cohorts: 15–19, 20–24, 25–29, 30–34, 35–39, 40–44, and 45–49 years.

### Statistical analysis

In this study, the burden of EDs was quantified using DALYs and the age-standardized DALY rate (ASDR). Using the formula [15], we calculated the ASDR per 100,000 women within the WCBA population. The ASDR is computed using the formula:

ASDR = $\frac{{\sum }_{i=1}^{N}{\alpha }_{i}{W}_{i}}{\sum_{i=1}^{N}Wi}$($\alpha_{i}$: the age-specific rate in the i-th age group; $W_{i}$: the number of individuals in age group i from the 2021 GBD standard population; N: the total number of age groups)

To analyze temporal trends in ASDR, we employed the estimated annual percentage change (EAPC), a metric widely used in epidemiologic studies for its ability to summarize long-term trends while minimizing the impact of short-term fluctuations [16]. This analysis was conducted using the stats R package. The computational formula is expressed as:

y = α + βx + ε.

EAPC = 100 × (exp(β) − 1).

(x: the year; y: the natural logarithm of the rate; α: the intercept; β: the slope; ε: the random error term).

If the EAPC and its 95% confidence intervals (CIs) are positive, this indicates an increasing trend in ASDR; if negative, a decreasing trend. If the 95% CI includes 0, the trend is statistically non-significant.

To characterize the global spatial distribution and regional disparities in ED burden, we generated choropleth maps using the ggplot2 and sf packages in R. To evaluate the relationship between socioeconomic status and disease burden, we performed Spearman’s rank correlation analysis between the ASDR and the SDI. This non-parametric method was selected over Pearson’s correlation to accommodate potential non-linear associations and non-normally distributed data. Finally, to project the future burden of EDs among WCBA through 2035, we applied a Bayesian Age-Period-Cohort (BAPC) model [17]. This predictive framework, implemented using the INLA and BAPC packages, systematically integrates age, period, and cohort effects to forecast disease trends based on GBD data from 1990 to 2021.

All statistical analyses and data visualizations were performed using R software (version 4.3.2). Descriptive statistics are reported as means with corresponding 95% uncertainty intervals (UIs) or 95% CIs. UIs are employed for GBD burden estimates to account for uncertainties arising from the modeling and simulation process, whereas CIs are utilized for regression-based parameters, such as the EAPC, to reflect the precision of the trend estimates. For all hypothesis tests and trend analyses, a *P*-value of < 0.05 was considered statistically significant.

## Results

### Burden of EDs at the global level

**Fig. 1:**
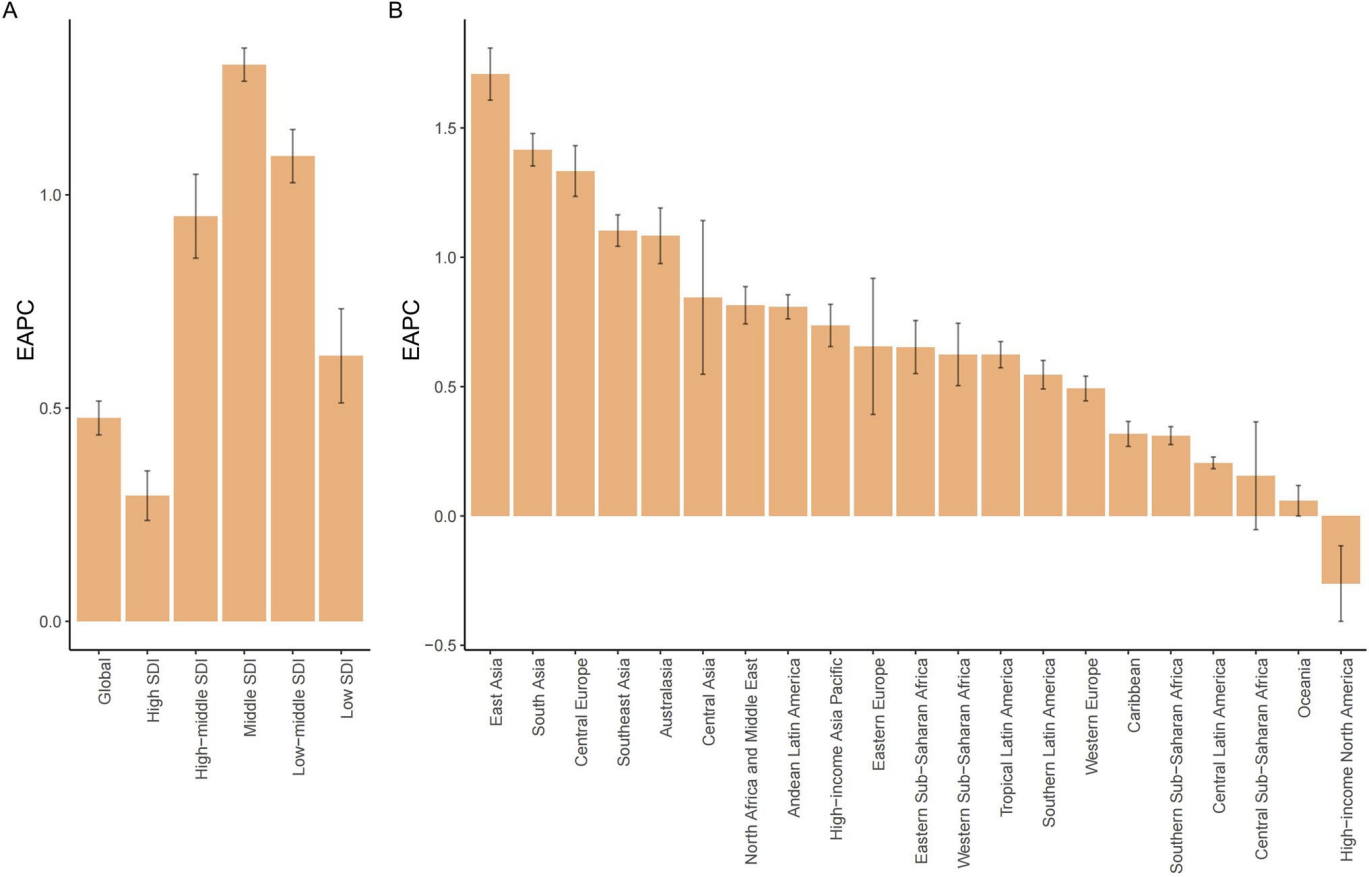
EAPC of DALYs for EDs at the global and regional levels. *EAPC* estimated annual percentage change, *DALYs* disability-adjusted life years, *EDs* eating disorders

In 2021, the global burden of EDs among WCBA amounted to 2,035,736.39 DALYs (95% UI: 1,229,250.35–3,188,528.83). Between 1990 and 2021, the ASDR for EDs in this population rose from 93.45 to 106.05 per 100,000, reflecting an EAPC of 0.48 (95% CI 0.44–0.52) (Table 1 and Fig. 1).

**Table 1:** DALYs of ED cases among WCBA in 1990 and 2021 at the global and regional levels, along with their EAPCs from 1990 to 2021

Location	DALYs				
	Number of cases (95% UI)		ASDR per 100,000 population (95% UI)		EAPC (95% CI)
	1990	2021	1990	2021	1990–2021
Global	1,307,570.07 (785,068.12, 2,056,686.38)	2,035,736.39 (1,229,250.35, 3,188,528.83)	93.45 (53.21, 149.85)	106.05 (59.77, 171.79)	0.48 (0.44, 0.52)
Low SDI	62,618.00 (37,609.16, 100,335.90)	175,878.57 (105,156.01, 283,816.38)	51.68 (27.92, 85.47)	59.66 (32.39, 98.80)	0.62 (0.51, 0.73)
Low-middle SDI	173,444.96 (103,870.86, 274,240.35)	414,121.32 (245,542.75, 660,954.16)	59.20 (32.17, 97.33)	79.50 (43.02, 131.52)	1.09 (1.03, 1.15)
Middle SDI	306,749.89 (180,265.91, 494,694.62)	550,506.86 (331,608.48, 875,004.49)	63.59 (34.68, 104.72)	92.51 (50.97, 152.56)	1.31 (1.27, 1.34)
High-middle SDI	270,694.27 (164,422.86, 423,382.94)	334,040.08 (200,773.65, 522,135.02)	94.34 (53.93, 150.48)	120.98 (67.87, 196.58)	0.95 (0.85, 1.05)
High SDI	492,866.05 (297,555.22, 759,888.09)	559,598.29 (342,876.52, 860,248.11)	220.89 (127.84, 352.88)	248.65 (146.04, 397.30)	0.29 (0.24, 0.35)
High-income Asia Pacific	84,727.99 (51,450.80, 130,376.62)	82,024.53 (50,562.14, 125,265.15)	192.64 (111.66, 305.91)	246.91 (147.00, 388.78)	0.74 (0.65, 0.82)
High-income North America	202,339.88 (121,226.81, 313,930.17)	215,000.67 (130,108.65, 336,158.34)	276.33 (158.33, 446.84)	268.19 (154.39, 434.05)	− 0.26 (− 0.41, − 0.12)
Western Europe	250,428.44 (154,172.64, 382,131.05)	262,347.72 (161,676.55, 403,250.16)	265.90 (154.94, 416.88)	305.23 (177.80, 485.02)	0.49 (0.44, 0.54)
Australasia	21,395.72 (12,822.31, 33,648.73)	36,317.72 (23,449.72, 52,913.43)	402.63 (226.15, 655.76)	530.29 (326.15, 808.17)	1.08 (0.98, 1.19)
Andean Latin America	15,971.80 (8786.45, 26,373.12)	34,752.68 (19,687.74, 56,996.72)	155.87 (80.44, 266.07)	196.16 (103.08, 333.67)	0.81 (0.76, 0.86)
Tropical Latin America	49,084.27 (28,112.07, 78,435.30)	77,877.62 (46,033.36, 123,066.03)	114.95 (62.88, 189.89)	133.83 (73.75, 218.02)	0.62 (0.57, 0.67)
Central Latin America	61,148.40 (34,523.37, 99,572.72)	95,675.98 (54,124.02, 155,067.45)	133.83 (71.32, 225.64)	141.17 (75.32, 236.45)	0.21 (0.18, 0.23)
Southern Latin America	21,470.21 (12,764.65, 33,684.58)	34,304.87 (20,017.15, 53,404.25)	170.40 (93.42, 282.70)	202.00 (110.36, 335.39)	0.55 (0.49, 0.60)
Caribbean	11,864.78 (6879.73, 18,820.17)	14,982.26 (8633.26, 24,113.03)	119.42 (63.58, 197)	125.31 (66.90, 211.73)	0.32 (0.27, 0.37)
Central Europe	24,045.01 (14,629.26, 37,872.10)	25,395.99 (15,690.34, 39,095.29)	80.29 (44.85, 131.31)	111.16 (63.38, 179.13)	1.33 (1.24, 1.43)
Eastern Europe	52,288.83 (32,109.55, 81,497.05)	44,215.36 (27,345.04, 68,855.49)	95.71 (54.04, 155.61)	104.78 (59.63, 169.53)	0.66 (0.39, 0.92)
Central Asia	13,145.13 (7963.20, 20,631.85)	19,836.59 (12,142.92, 31,553.38)	72.03 (40.07, 118.79)	82.80 (45.79, 135.74)	0.84 (0.55, 1.14)
North Africa and Middle East	86,410.97 (49,883.31, 138,556.42)	194,127.61 (113,480.53, 309,277.81)	103.02 (55.18, 171.72)	122.23 (65.55, 204.47)	0.81(0.74, 0.89)
South Asia	144,502.42 (86,292.42, 230,468.16)	400,821.43 (239,087.77, 639,261.67)	53.23 (28.71, 88.53)	79.25 (43.05, 130.22)	1.42 (1.35, 1.48)
Southeast Asia	58,625.02 (35,360.69, 94,032.86)	114,842.71 (68,658.37, 184,298.57)	45.23 (24.69, 74.33)	63.91 (34.88, 104.77)	1.10 (1.04, 1.16)
East Asia	136,318.21 (80,335.40, 215,817.44)	185,217.30 (112,763.75, 295,431.33)	38.23 (20.80, 63.05)	62.59 (34.12, 103.46)	1.71 (1.61, 1.81)
Oceania	775.25 (452.71, 1254.84)	1744.59 (1046.14, 2799.29)	46.16 (23.77, 77.98)	48.53 (25.42, 83.09)	0.06 (− 0.00, 0.12)
Western Sub-Saharan Africa	29,552.10 (17,590.90, 47,700.82)	89,922.36 (53,718.73, 144,892.87)	61.53 (33.30, 101.45)	69.47 (37.57, 114.47)	0.62 (0.50, 0.74)
Eastern Sub-Saharan Africa	23,261.41 (13,815.76, 37,374.91)	66,315.73 (39,656.81, 107,450.29)	48.78 (26.57, 81.56)	57.13 (30.64, 94.57)	0.65 (0.55, 0.76)
Central Sub-Saharan Africa	7796.69 (4519.21, 12,187.19)	19,790.86 (11,412.97, 31,432.25)	57.85 (29.90, 97.34)	56.49 (29.66, 93.62)	0.16 (− 0.05, 0.36)
Southern Sub-Saharan Africa	12,417.53 (7384.15, 19,944.16)	20,221.80 (12,156.69, 32,276.70)	86.2 (46.76, 141.99)	91.58 (50.09, 150.21)	0.31 (0.28, 0.35)

*DALYs* disability-adjusted life-years, *ED* eating disorder, *WCBA* women of childbearing age, *ASDR* age-standardized DALY rate, *EAPC* estimated annual percentage change, *UI* uncertainty interval, *CI* confidence interval

**Fig. 2:**
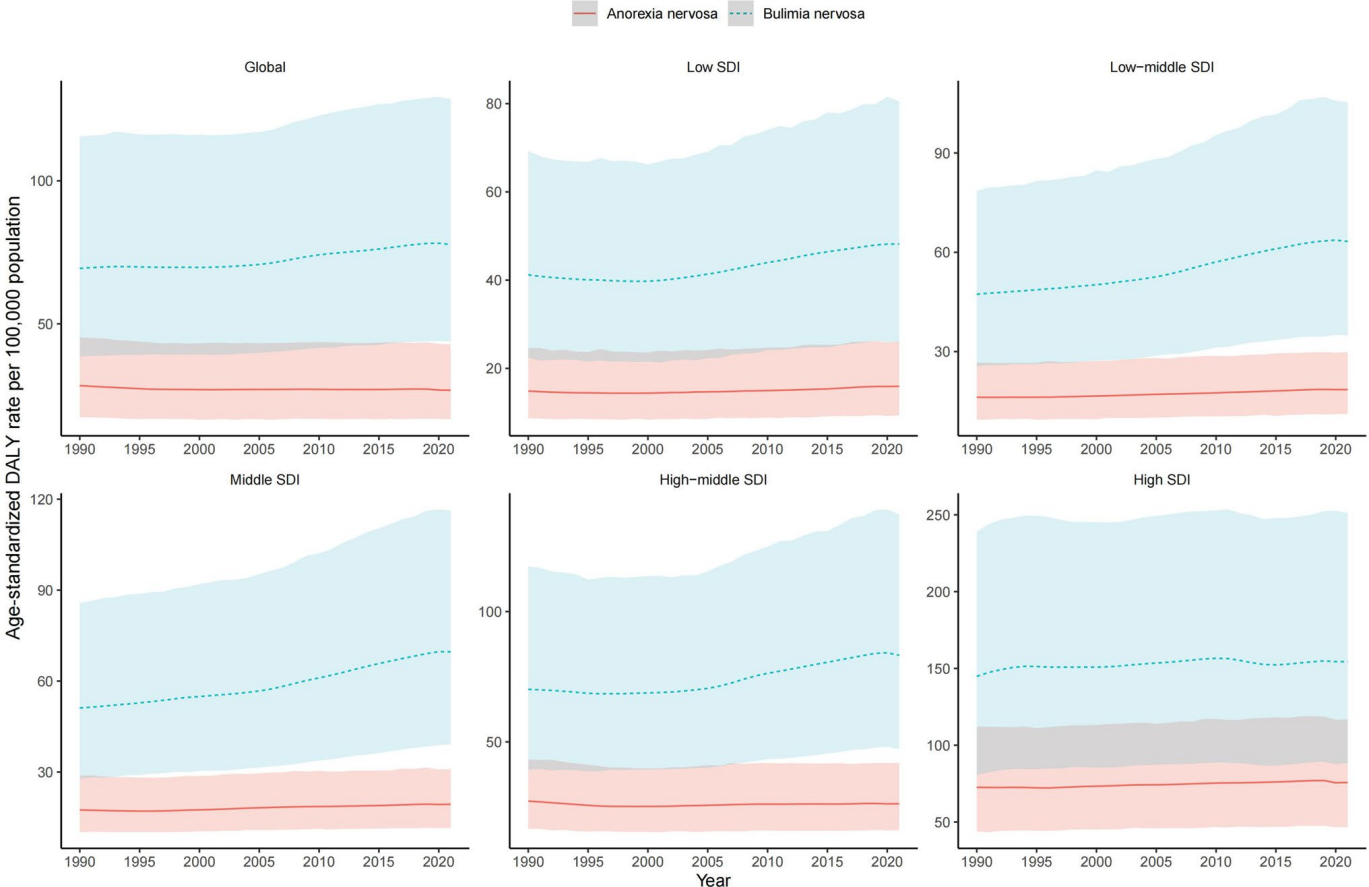
ASDR for AN and BN at the global and SDI regional levels. *ASDR* age-standardized DALY rate, *AN* anorexia nervosa, *BN* bulimia nervosa, *SDI* sociodemographic index

When stratified by subtype, the global disease burden of BN consistently exceeded that of AN throughout the study period (Fig. 2). Comprehensive data on DALYs, ASDR trends, and EAPCs for AN and BN are provided in Supplementary Tables S1–S2 and Supplementary Figs. S1–S2.

### Burden of EDs at the SDI regional level

Analysis by SDI quintile revealed that in 2021, WCBA in high-SDI regions exhibited the highest ASDR for EDs, while those in low-SDI regions had the lowest (Table 1). This pattern held consistent for both specific subtypes (AN and BN).

Longitudinally, the ASDR for EDs increased across all SDI regions from 1990 to 2021, with the most rapid growth observed in middle-SDI regions (EAPC = 1.31, 95% CI 1.27–1.34) (Fig. 1). Throughout the study period, the burden of BN remained higher than that of AN across all SDI categories (Fig. 2). Specific ASDR values and 95% UIs for each SDI region are detailed in Supplementary Tables S1–S2.

### Burden of EDs at the geographic regional level

Among the 21 GBD regions, Australasia had the highest ASDR for EDs in 2021, followed by Western Europe, while Oceania had the lowest (Table 1). From 1990 to 2021, the ASDR for EDs increased in all regions except High-income North America, which showed a slight decline (EAPC = − 0.26, 95% CI − 0.41 to − 0.12). East Asia experienced the largest EAPC in ED ASDR during this period (Fig. 1).

Australasia recorded the highest ASDRs for both AN and BN in 2021. Central Europe had the largest EAPC for AN ASDR, while East Asia had the largest EAPC for BN ASDR from 1990 to 2021. Detailed regional data on AN and BN are provided in Supplementary Tables S1–S2 and Supplementary Figs. S1–S2.

### Burden of EDs at the national level

**Fig. 3:**
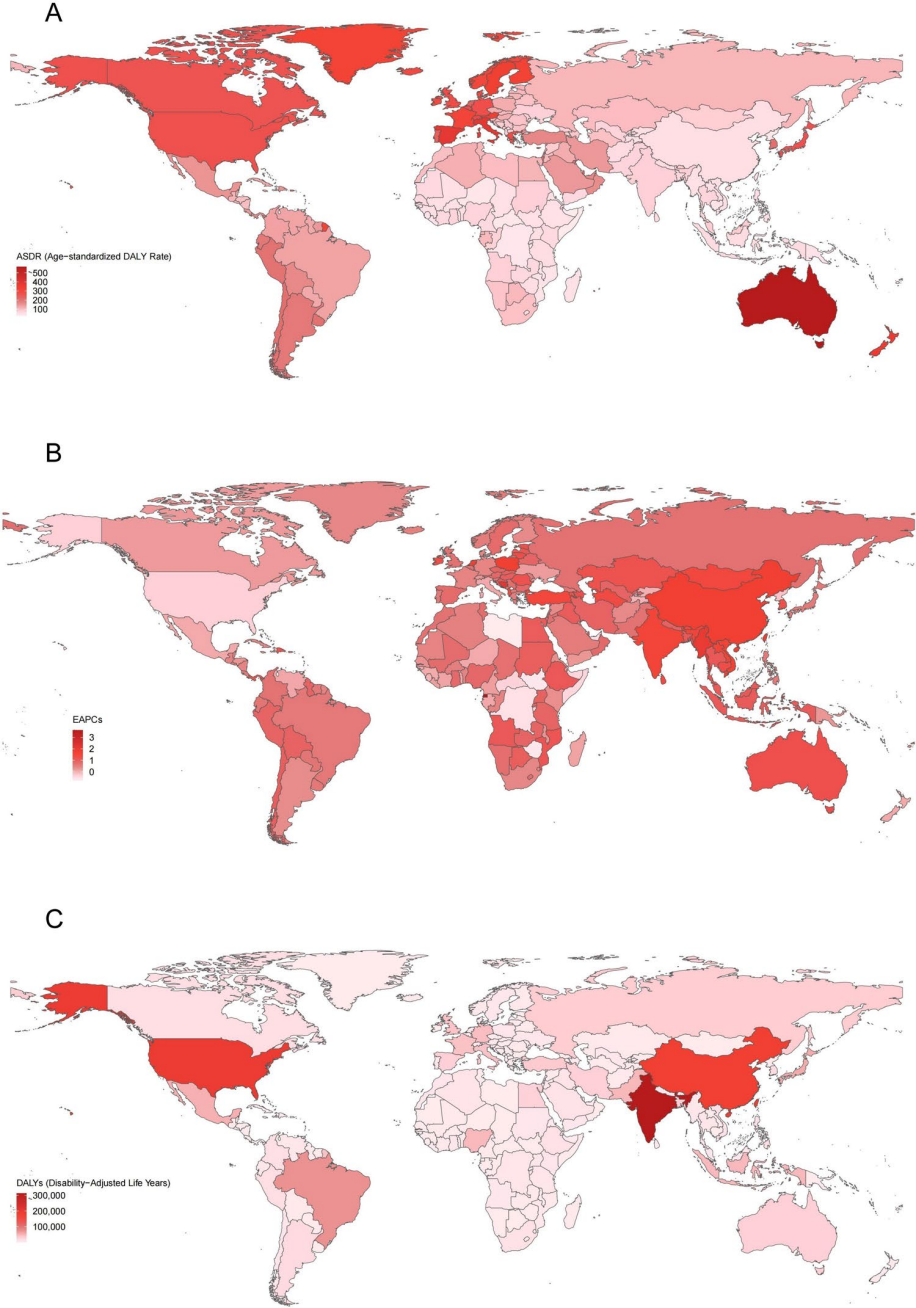
EDs burden in 204 countries and territories. **A** The ASDR in 2021; **B** EAPC in ASDR from 1990 to 2021; **C** DALYs in 2021. *EDs* eating disorders, *ASDR* age-standardized DALY rate, *EAPC* estimated annual percentage change, *DALYs* disability-adjusted life years

In 2021, Australia had the highest ED ASDR globally, while Equatorial Guinea exhibited the largest EAPC for ED ASDR from 1990 to 2021 (Fig. 3). In terms of total ED DALYs, India, the United States, and China were the top three countries globally.

Monaco had the highest AN ASDR, and Australia had the highest BN ASDR among WCBA worldwide in 2021. Equatorial Guinea showed the largest EAPC for both AN and BN ASDRs during the study period. India also accounted for the highest total DALYs for both AN and BN globally. All detailed national-level data on EDs, AN, and BN are presented in Supplementary Tables S3–S5 and Supplementary Figs. S3–S4.

### The changing patterns at different SDI levels and baseline burden

**Fig. 4:**
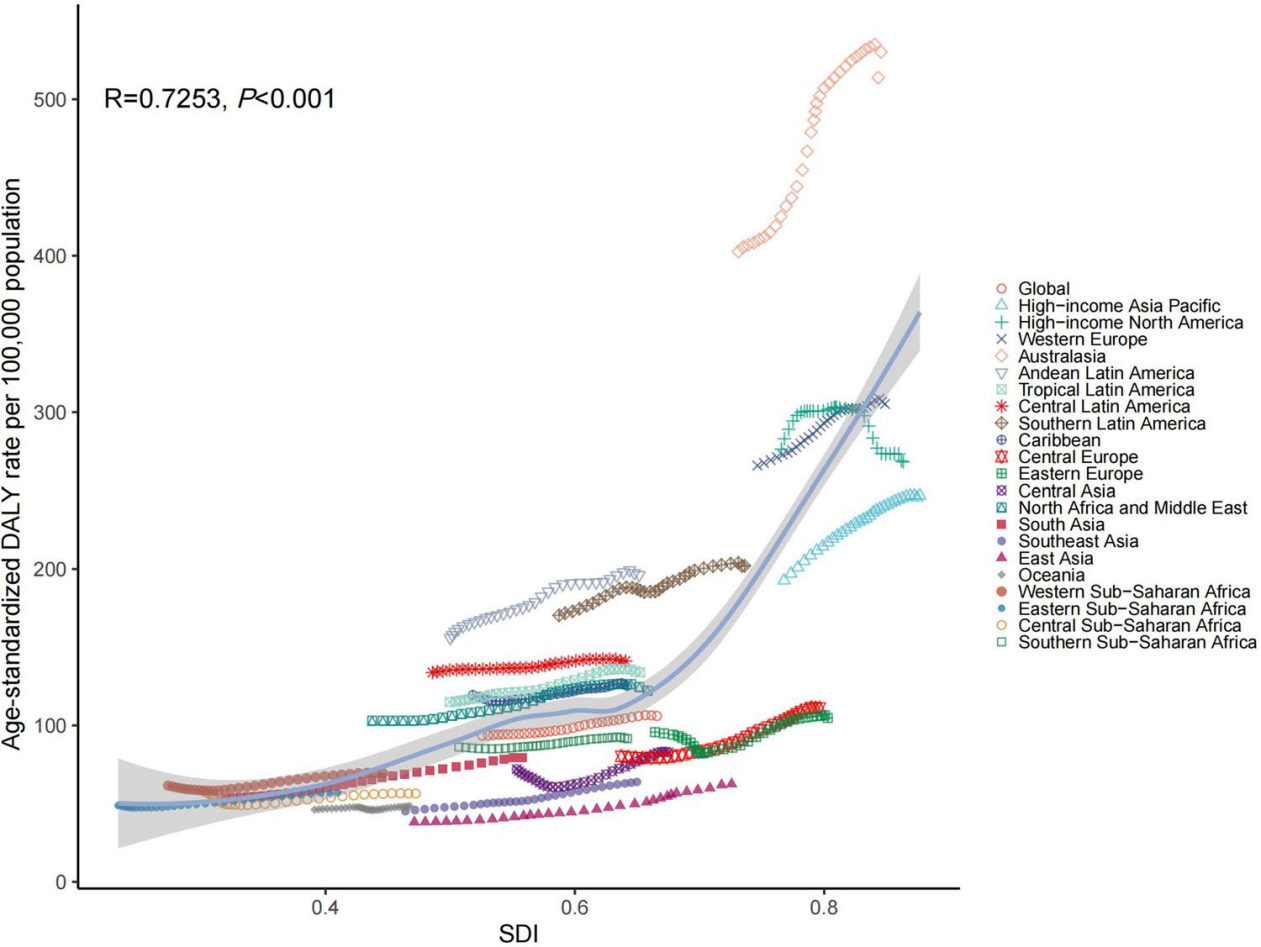
The associations between the SDI and ASDR of EDs across 21 GBD regions. *SDI* sociodemographic index, *ASDR* age-standardized DALY rate, *EDs* eating disorders, *GBD* Global Burden of Disease

**Fig. 5:**
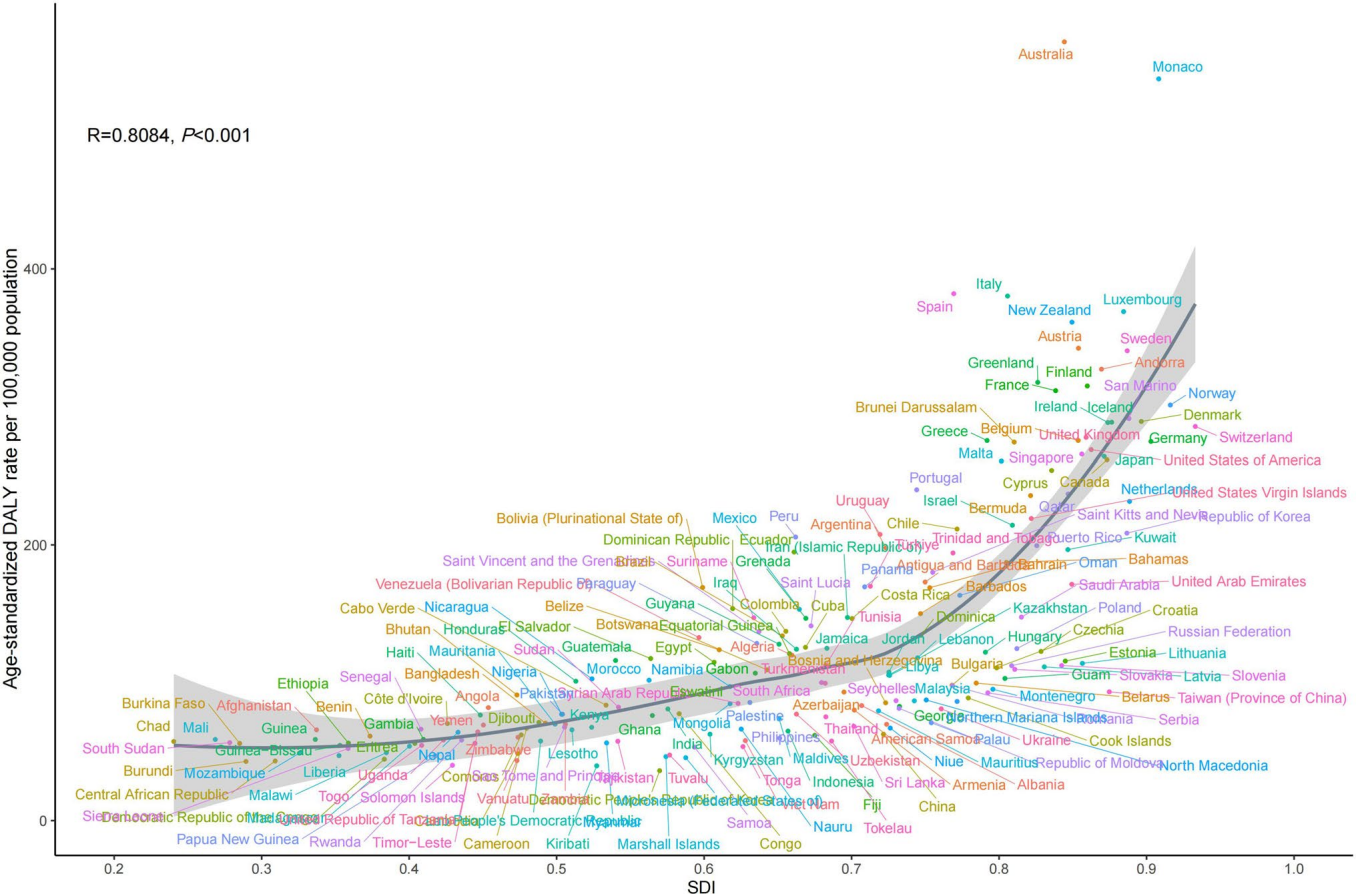
The associations between the SDI and ASDR of EDs across 204 countries and territories. *SDI* sociodemographic index, *ASDR* age-standardized DALY rate, *EDs* eating disorders

A strong positive correlation was identified between the ASDR of EDs and SDI at both the regional level (*r* = 0.725, *P* < 0.001; Fig. 4) and the national level (*r* = 0.808, *P* < 0.001; Fig. 5) over the 1990–2021 period. Significant positive correlations were similarly observed for both AN and BN individually. Detailed correlation coefficients and visual plots are available in Supplementary Figs. S5–S8.

### Prediction analysis of ED burden

**Fig. 6:**
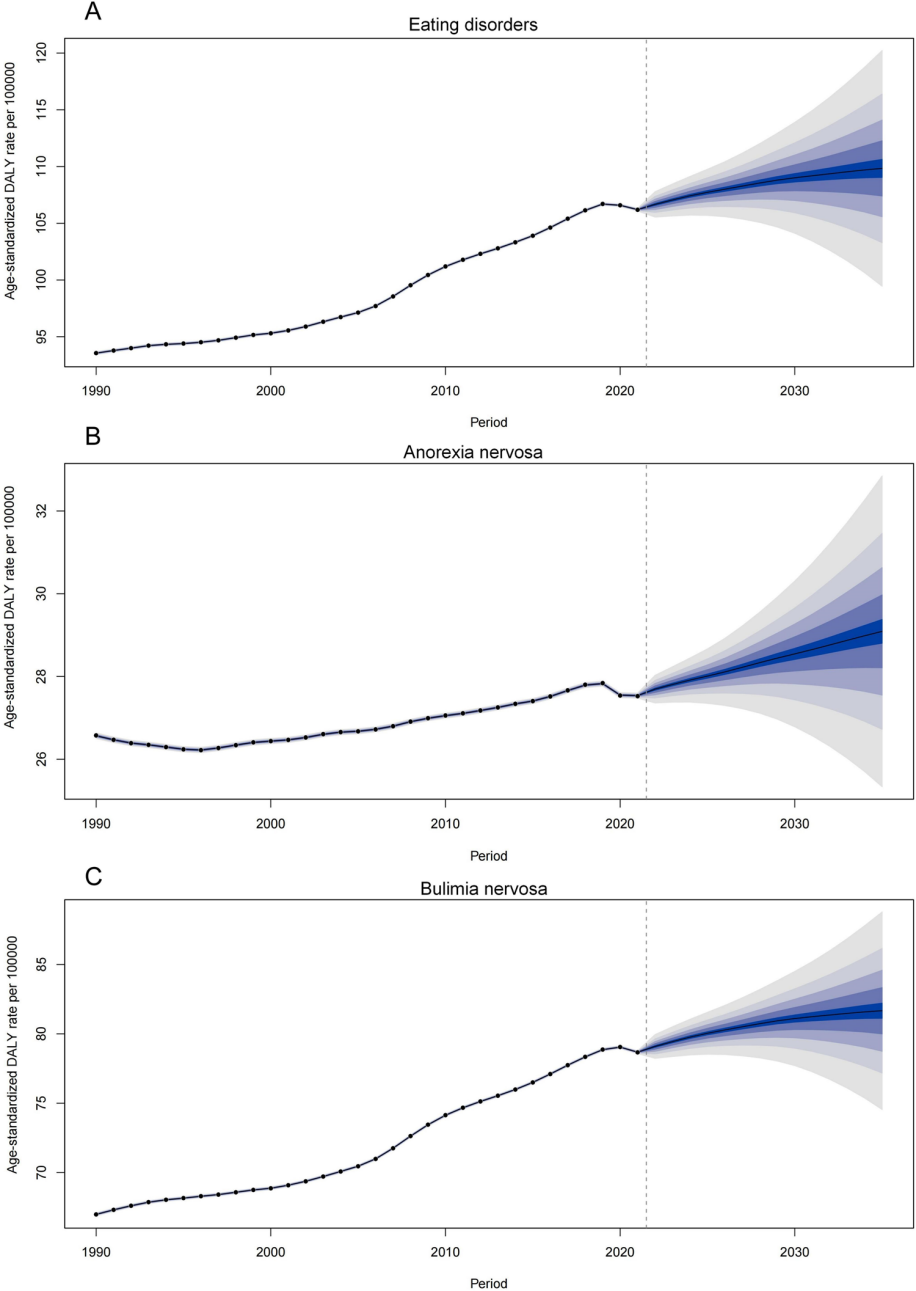
Future predictions of the global burden of EDs among WCBA from 2022 to 2035. **A** EDs; **B** AN; **C** BN. *EDs* eating disorders, *WCBA* women of childbearing age, *AN* anorexia nervosa, *BN* bulimia nervosa

Assuming current trends persist without major shifts in diagnostic criteria or intervention policy, the global ASDR for EDs among WCBA is projected to reach 109.84 per 100,000 population by 2035. Similar upward trajectories are projected for both AN and BN (Table S6 and Fig. 6).

Age-stratified projections indicate that by 2035, the 20–24 age group will exhibit the highest burden for total EDs and AN, while the 25–29 age group is expected to account for the highest burden for BN. Detailed projections are available in Supplementary Table S6 and Supplementary Figs. S9–S11.

## Discussion

Over the past 32 years, the global burden of EDs among WCBA has increased substantially. Based on GBD 1990–2021 data, total DALYs increased by more than 50% by 2021 compared with 1990, and this upward trend is projected to continue through 2035. It should be noted, however, that because the GBD 2021 framework includes only AN and BN and excludes BED and OSFED, our estimates likely understate the overall ED burden.

The rising burden observed in our study is likely multifactorial, potentially reflecting a complex interplay of sociocultural and biological factors that warrant further mechanistic investigation. Media-driven thin ideals, weight stigma, and peer norms have been associated with body dissatisfaction and maladaptive weight-control behaviors [18–23]. These influences may coexist with biological susceptibilities in WCBA, including hormonal fluctuations and PCOS [24–26].

Our findings highlight a particularly high burden among women aged 20–29 years, which overlaps with the peak reproductive period. In contrast to postpartum depression rates of approximately 13–19% in the general population, 40.2% of pregnant women with an ED history report worsening symptoms, including binge eating and body image concerns [27, 28]. Postpartum stress has also been associated with an increased risk of relapse [29]. Taken together, these findings support incorporating ED risk screening into routine reproductive care at key touchpoints, such as the first prenatal visit and early postpartum follow-up [8, 30]. Screening can combine brief validated tools with targeted history-taking focusing on restrictive eating, binge eating, compensatory behaviors, and body-image distress [31]. For individuals who screen positive, a stepped-care pathway may be adopted, including risk stratification, referral, and coordinated follow-up across obstetrics and gynecology, primary care, and mental health services, consistent with emerging models of integrated ED care [32].

Consistent with prior population-based studies, we observed a positive association between ED burden and the SDI [4, 33]. In high-SDI settings, higher ASDRs may reflect both a greater underlying burden and improved detection, possibly related to more developed health systems and greater mental health awareness. In addition, sociocultural pressures related to appearance ideals and perfectionism have been associated with increased ED risk in these contexts [34, 35].

Conversely, lower ASDRs in low-SDI regions may reflect underdetection rather than a genuinely lower burden [18, 36]. This pattern may be related to limited mental health workforce capacity and the scarcity of culturally validated screening instruments [37, 38]. Persistent stigma and beliefs framing EDs as “disorders of affluence” may also be associated with reduced help-seeking and may further obscure the true epidemiological burden [39].

Regionally, East Asia experienced the fastest increase in ED burden from 1990 to 2021, followed by South Asia. This trend may be associated with rapid socioeconomic transition and lifestyle changes, including Westernized dietary patterns and increased exposure to Western beauty ideals through social media [40, 41]. However, GBD estimates are not designed for causal inference, and longitudinal primary studies are needed to better understand the mechanisms underlying these patterns in rapidly urbanizing settings.

Across subtypes, BN contributed a consistently greater burden than AN globally and across all SDI regions, consistent with BN’s higher prevalence despite the higher mortality associated with AN [12]. Because GBD 2021 estimates include only AN and BN, disorders such as BED and OSFED are not captured. Clinical evidence indicates that BED and OSFED account for a substantial proportion of ED cases (approximately 30% and 41%, respectively) [12], implying that the overall ED burden may be higher than that reflected in current GBD estimates.

Our analysis suggests that the global burden of EDs among WCBA is projected to increase through 2035 based on BAPC modeling. Given the substantial financial costs to healthcare systems [42, 43], targeted prevention and early detection strategies may be warranted. As an example of a national response in a high-burden setting, Australia launched the National Eating Disorders Research and Translation Strategy in 2023 to prioritize prevention, early detection, and personalized care [44, 45].

These findings have implications for both policy and clinical practice. At the policy level, priorities may include strengthening ED surveillance, improving systems that capture a broader spectrum of eating disorders beyond AN and BN, and investing in mental health workforce capacity and culturally validated screening tools in low-SDI settings. At the clinical level, our findings highlight the importance of incorporating ED risk assessment within routine reproductive and primary care, particularly for women in the peak reproductive age range. Strengthening coordination across obstetrics/gynecology, primary care, and mental health services may help support early identification and continuity of care for affected women.

Future research may benefit from prioritizing improved global comparability of ED case definitions and measurement, expanding data coverage for underrepresented regions, and conducting multicenter cohort studies to better characterize trajectories and modifiable risk factors. Such efforts may help strengthen the evidence base needed to inform prevention strategies and policy responses.

### Strength and limits

This study provides a comprehensive global analysis of the ED burden specifically among WCBA from 1990 to 2021, incorporating future projections through 2035. However, several limitations should be acknowledged when interpreting these findings. First, estimates depend on country-level reporting capacity, and heterogeneity in data quality may affect cross-national comparability. Second, the exclusion of BED and OSFED in GBD estimates may result in underestimation of the total burden of EDs. Third, the descriptive design precludes causal inference or identification of individual-level risk factors. Fourth, temporal changes in diagnostic practices and awareness may influence trend estimates. Finally, DALYs may not fully capture psychosocial impairment and effects on family functioning associated with EDs.

## Conclusion

Our analysis shows that the global burden of EDs among WCBA increased from 1990 to 2021 and is projected to rise further through 2035. BN consistently contributed a greater burden than AN, and ED burden was positively associated with SDI. These findings support prioritizing BN and high-burden regions and strengthening integrated screening and care within reproductive health services.

### What is already known on this subject?

EDs are recognized as a significant public health challenge that disproportionately affects WCBA and severely impacts reproductive and neuroendocrine health. Prior to this study, most epidemiological data were derived from high-income Western nations, often obscuring the escalating burden in low- and middle-income regions undergoing rapid socioeconomic transition.

### What this study adds?

This study quantifies the global DALYs and temporal trends of AN and BN specifically for WCBA, revealing a substantial increase in burden, particularly within middle-SDI regions and East Asia. We found that BN consistently imposes a higher burden than AN in this demographic and that the peak burden is projected to shift toward the 20–29 age group by 2035. Our findings underscore the urgent need to integrate ED screening into routine obstetric and gynecological care to address the intersection of mental and reproductive health.

## Supplementary Information

Below is the link to the electronic supplementary material.

Supplementary Material 1. EAPC of DALYs for AN at the global and regional levels.

Supplementary Material 2. EAPC of DALYs for BN at the global and regional levels.

Supplementary Material 3. AN burden in 204 countries and territories. **A** The ASDR in 2021; **B** EAPC in ASDR from 1990 to 2021; **C** DALYs in 2021.

Supplementary Material 4. BN burden in 204 countries and territories. **A** The ASDR in 2021; **B** EAPC in ASDR from 1990 to 2021; **C** DALYs in 2021.

Supplementary Material 5. The associations between the SDI and ASDR of AN across 21 GBD regions.

Supplementary Material 6. The associations between the SDI and ASDR of BN across 21 GBD regions.

Supplementary Material 7. The associations between the SDI and ASDR of AN across 204 countries and territories.

Supplementary Material 8. The associations between the SDI and ASDR of BN across 204 countries and territories.

Supplementary Material 9. Future predictions of the global burden of EDs among WCBA at all age stages from 2022 to 2035.

Supplementary Material 10. Future predictions of the global burden of AN among WCBA at all age stages from 2022 to 2035.

Supplementary Material 11. Future predictions of the global burden of BN among WCBA at all age stages from 2022 to 2035.

Supplementary Material 12.

Supplementary Material 13.

Supplementary Material 14.

Supplementary Material 15.

Supplementary Material 16.

Supplementary Material 17.

## Data Availability

Additional details about the GBD database are available on the official website at https://ghdx.healthdata.org/gbd-2021/sources.

## Abbreviations

EDs: Eating disorders
AN: Anorexia nervosa
BN: Bulimia nervosa
BED: Binge eating disorder
WCBA: Women of childbearing age
GBD: The Global Burden of Diseases
DALYs: Disability-adjusted life years
ASDR: Age-standardized DALY rate
UI: Uncertainty interval
CI: Confidence interval
SDI: Sociodemographic index
EAPC: Estimated annual percentage change
BAPC: Bayesian age-period-cohort
DSM: Diagnostic and Statistical Manual of Mental Disorders
ICD: International Classification of Diseases
PCOS: Polycystic ovary syndrome
OSFED: Other specified feeding and eating disorders
