# Supplementary figures and images for: Patterns and trends of eating disorders among women of childbearing age: a comprehensive analysis from 1990 to 2021 with future predictions
Source: Eat Weight Disord. 2026 Mar 23;31(1):40. doi: 10.1007/s40519-026-01842-8 (PMC13132930; doi:10.1007/s40519-026-01842-8)

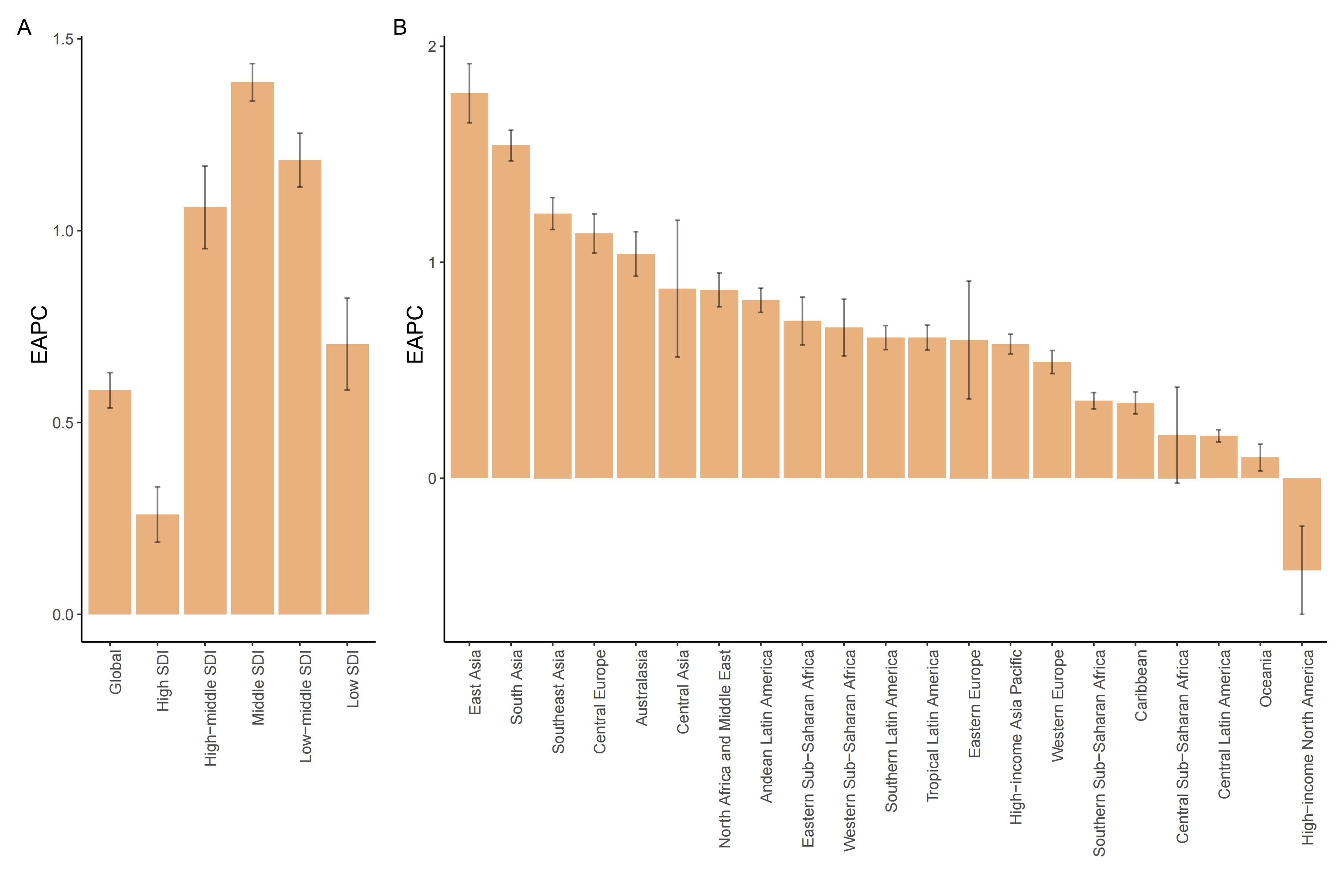

Supplement: Supplementary file 1 — Supplementary Material 1. EAPC of DALYs for AN at the global and regional levels. [file 40519_2026_1842_MOESM1_ESM.jpeg]

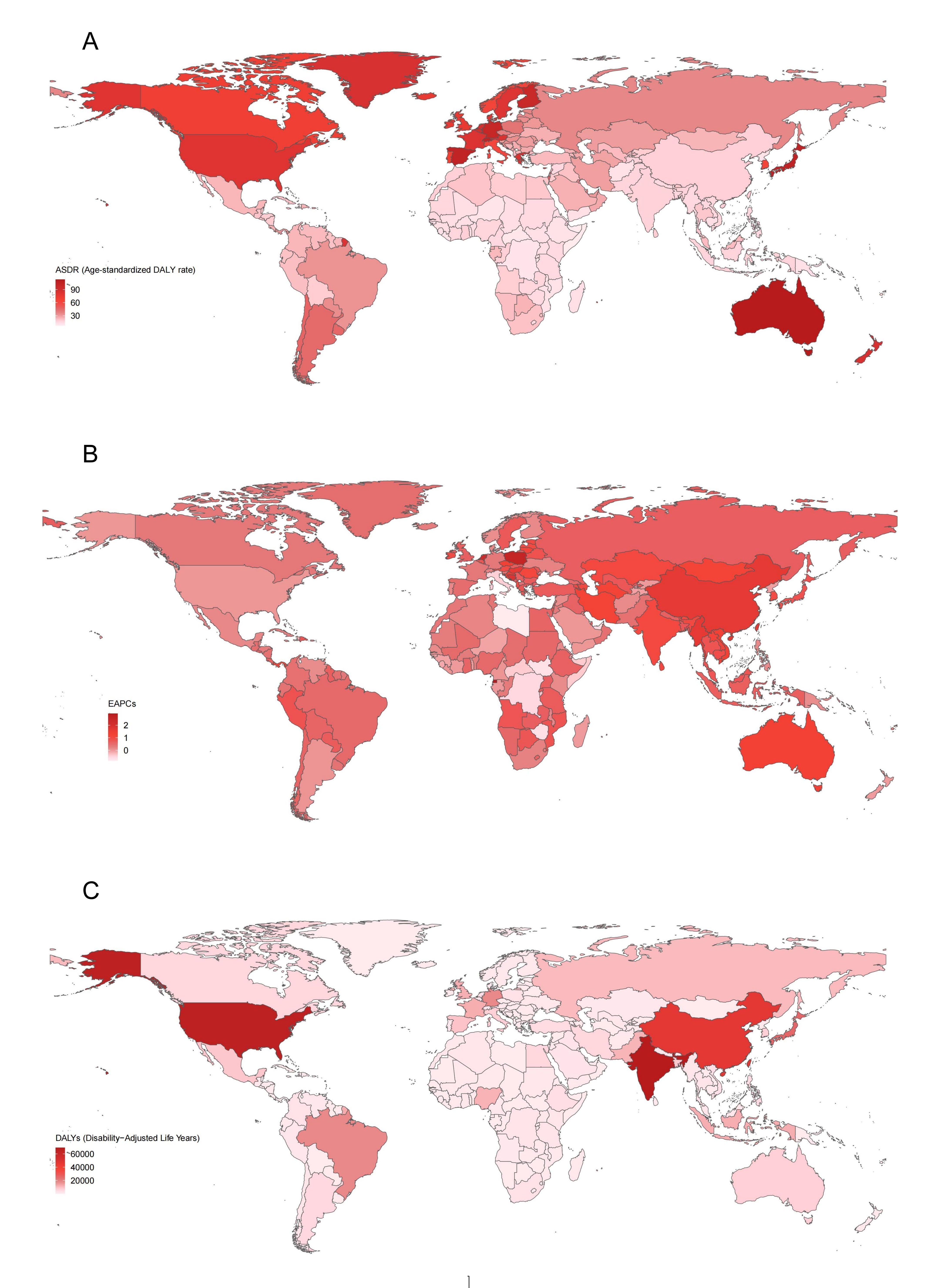

Supplement: Supplementary file 2 — Supplementary Material 2. EAPC of DALYs for BN at the global and regional levels. [file 40519_2026_1842_MOESM2_ESM.jpeg]

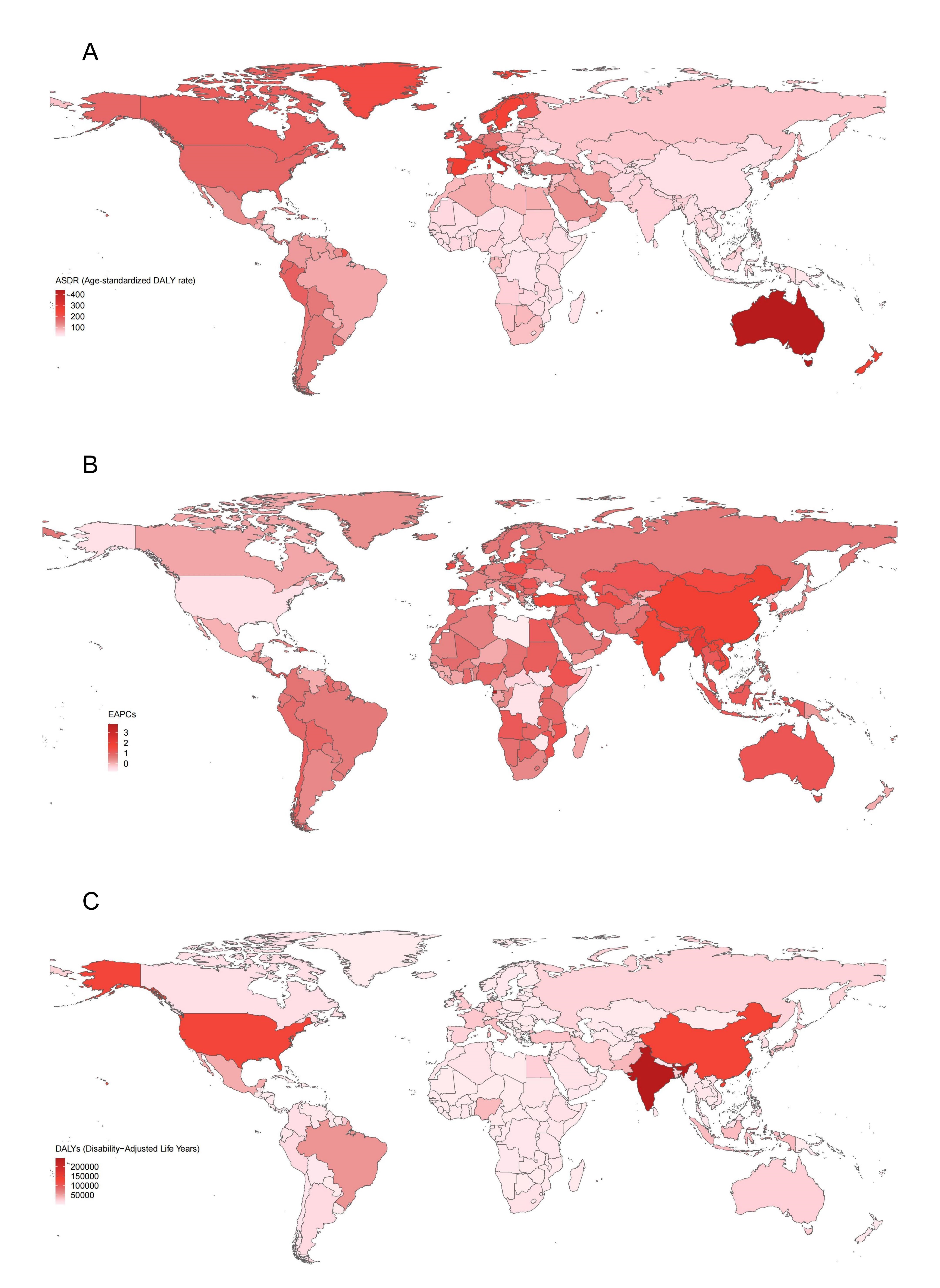

Supplement: Supplementary file 3 — Supplementary Material 3. AN burden in 204 countries and territories. A The ASDR in 2021; B EAPC in ASDR from 1990 to 2021; C DALYs in 2021. [file 40519_2026_1842_MOESM3_ESM.jpeg]

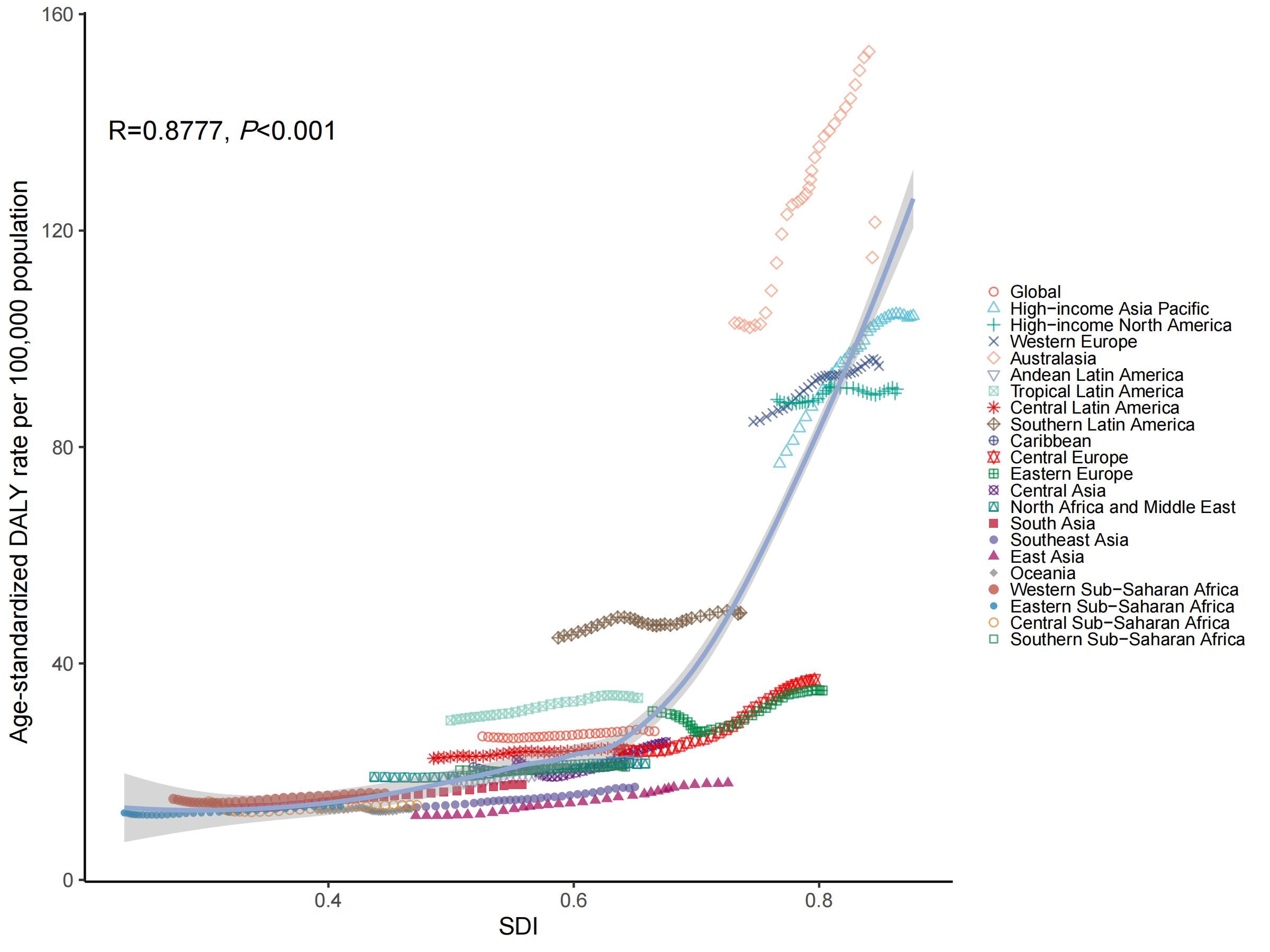

Supplement: Supplementary file 4 — Supplementary Material 4. BN burden in 204 countries and territories. A The ASDR in 2021; B EAPC in ASDR from 1990 to 2021; C DALYs in 2021. [file 40519_2026_1842_MOESM4_ESM.jpeg]

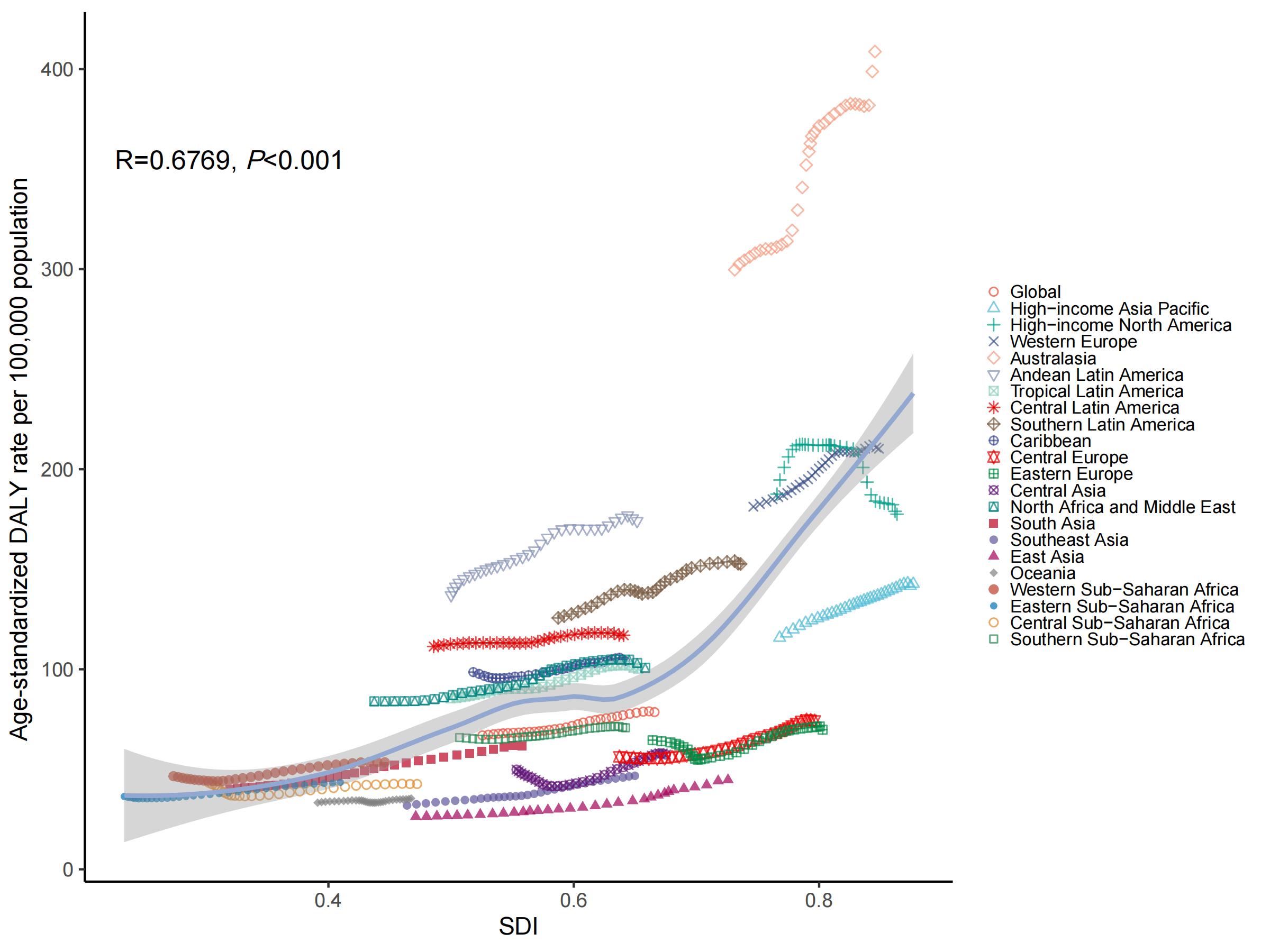

Supplement: Supplementary file 5 — Supplementary Material 5. The associations between the SDI and ASDR of AN across 21 GBD regions. [file 40519_2026_1842_MOESM5_ESM.jpeg]

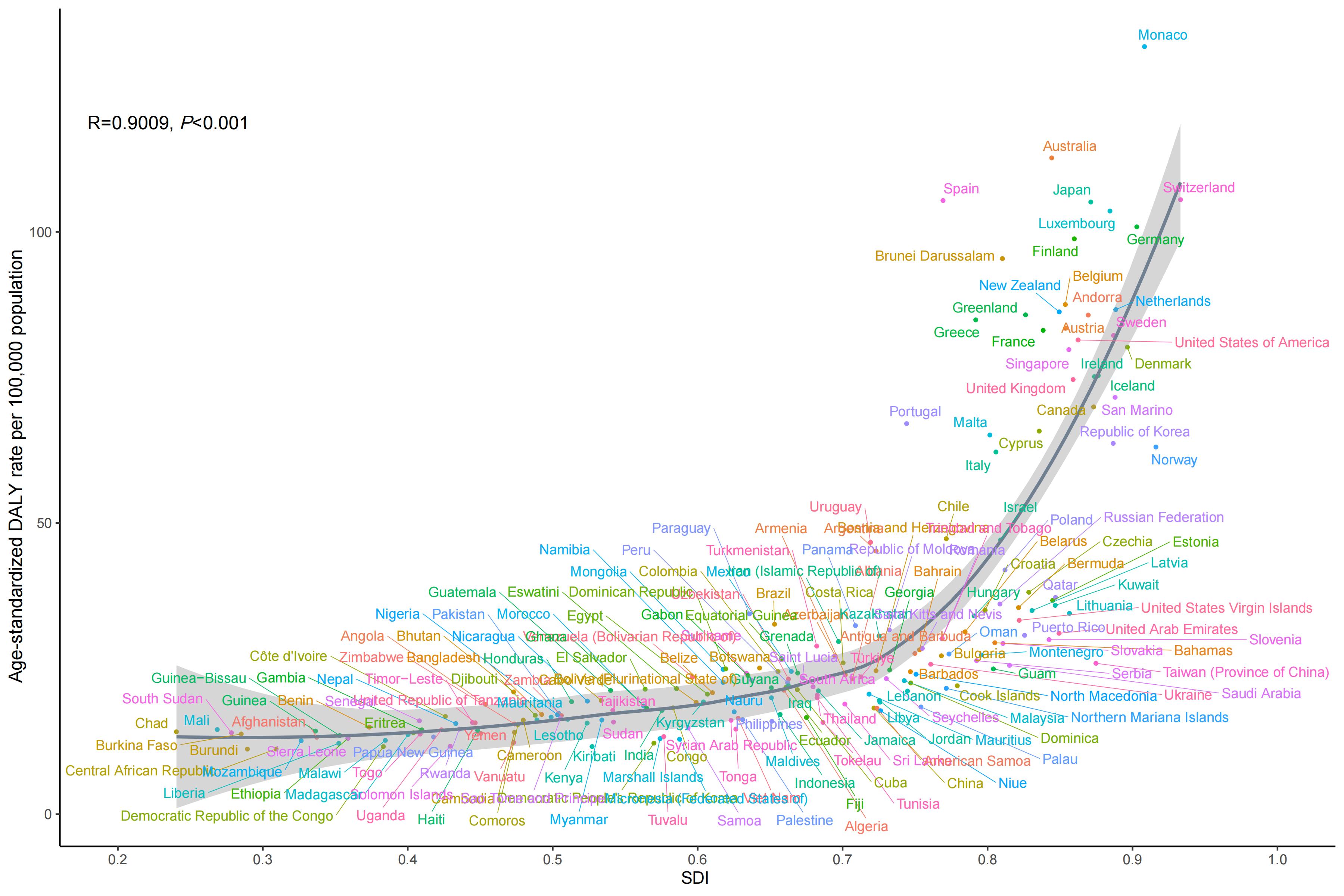

Supplement: Supplementary file 6 — Supplementary Material 6. The associations between the SDI and ASDR of BN across 21 GBD regions. [file 40519_2026_1842_MOESM6_ESM.jpeg]

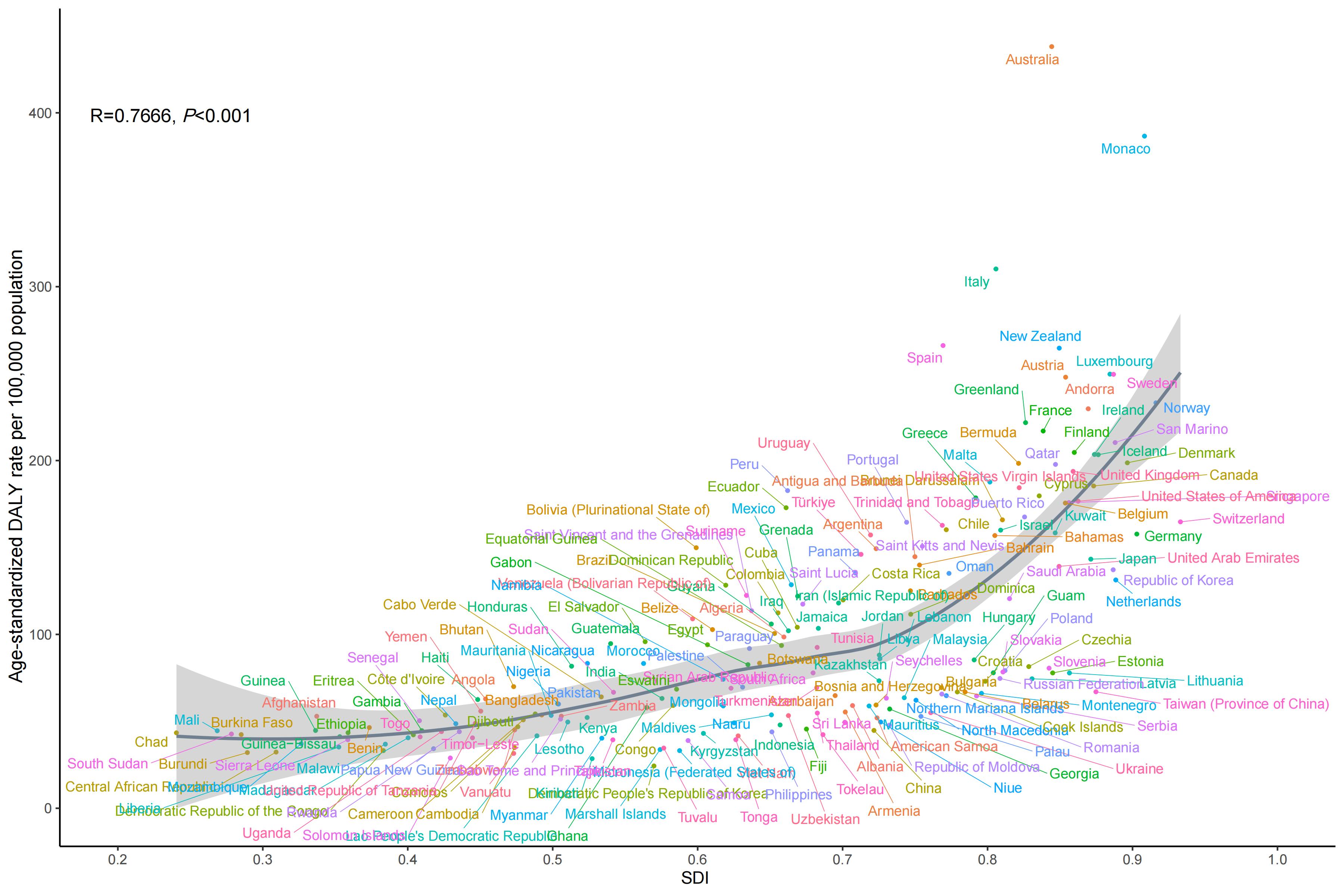

Supplement: Supplementary file 7 — Supplementary Material 7. The associations between the SDI and ASDR of AN across 204 countries and territories. [file 40519_2026_1842_MOESM7_ESM.jpeg]

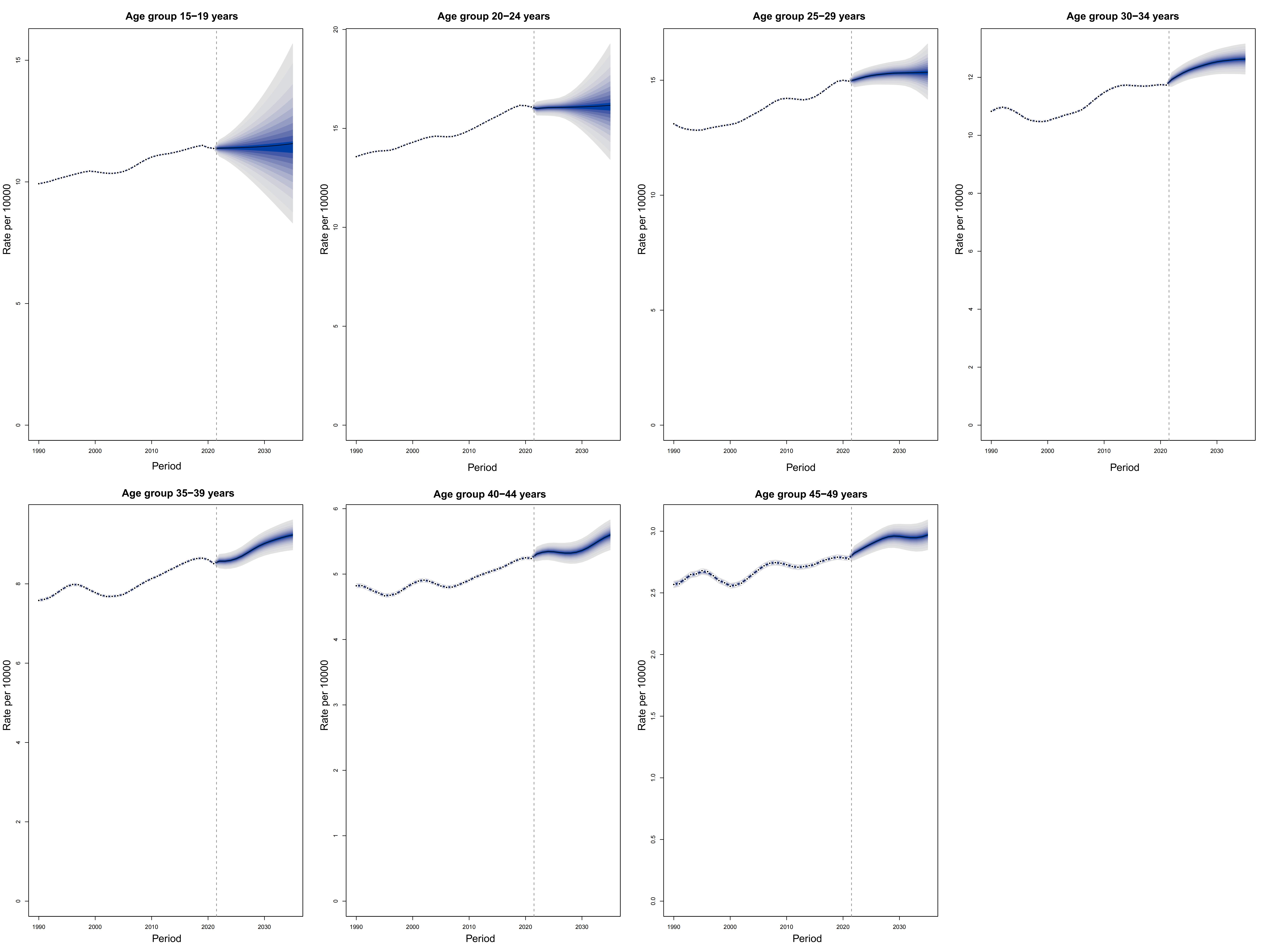

Supplement: Supplementary file 8 — Supplementary Material 8. The associations between the SDI and ASDR of BN across 204 countries and territories. [file 40519_2026_1842_MOESM8_ESM.jpeg]

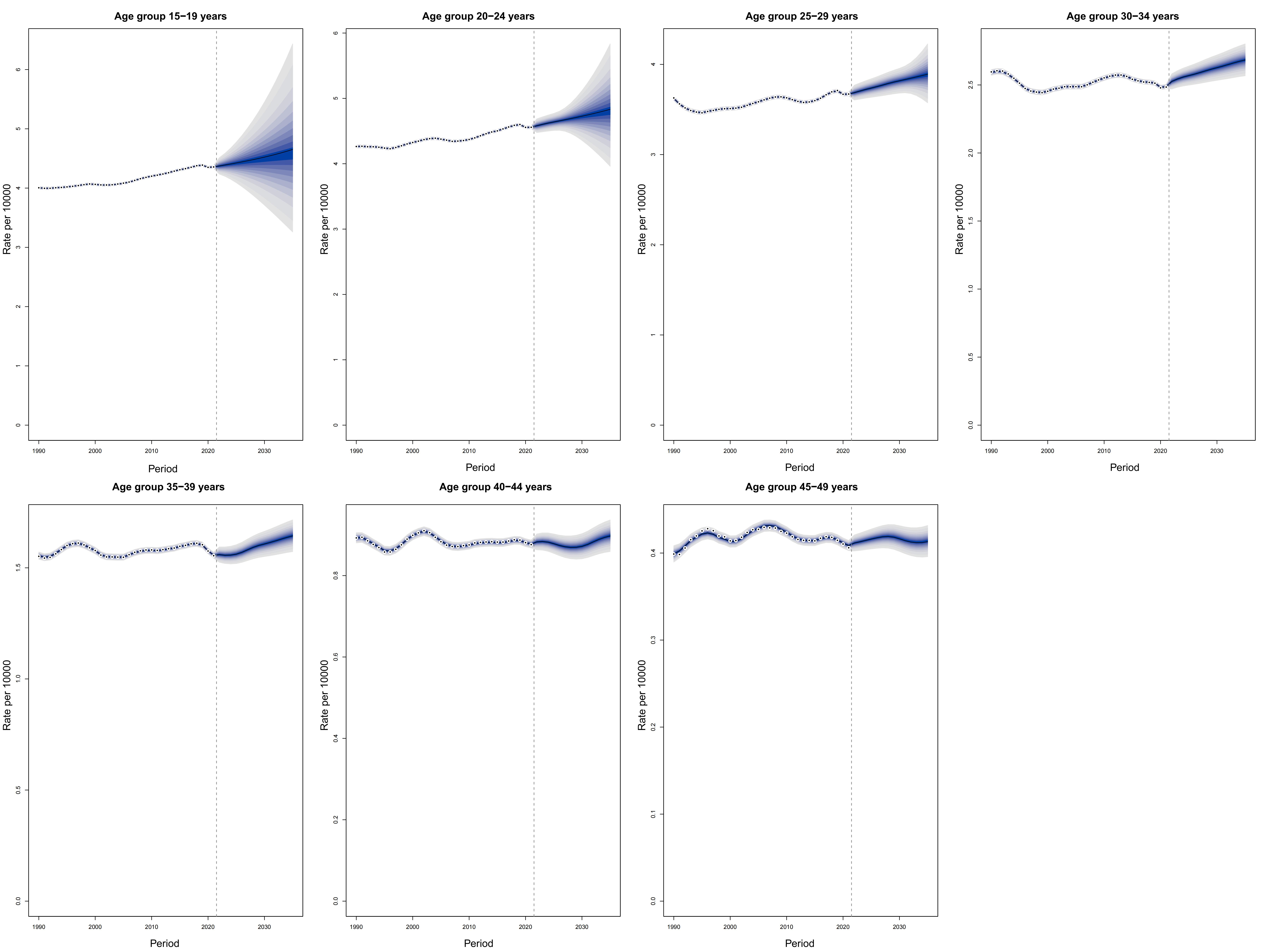

Supplement: Supplementary file 9 — Supplementary Material 9. Future predictions of the global burden of EDs among WCBA at all age stages from 2022 to 2035. [file 40519_2026_1842_MOESM9_ESM.jpeg]

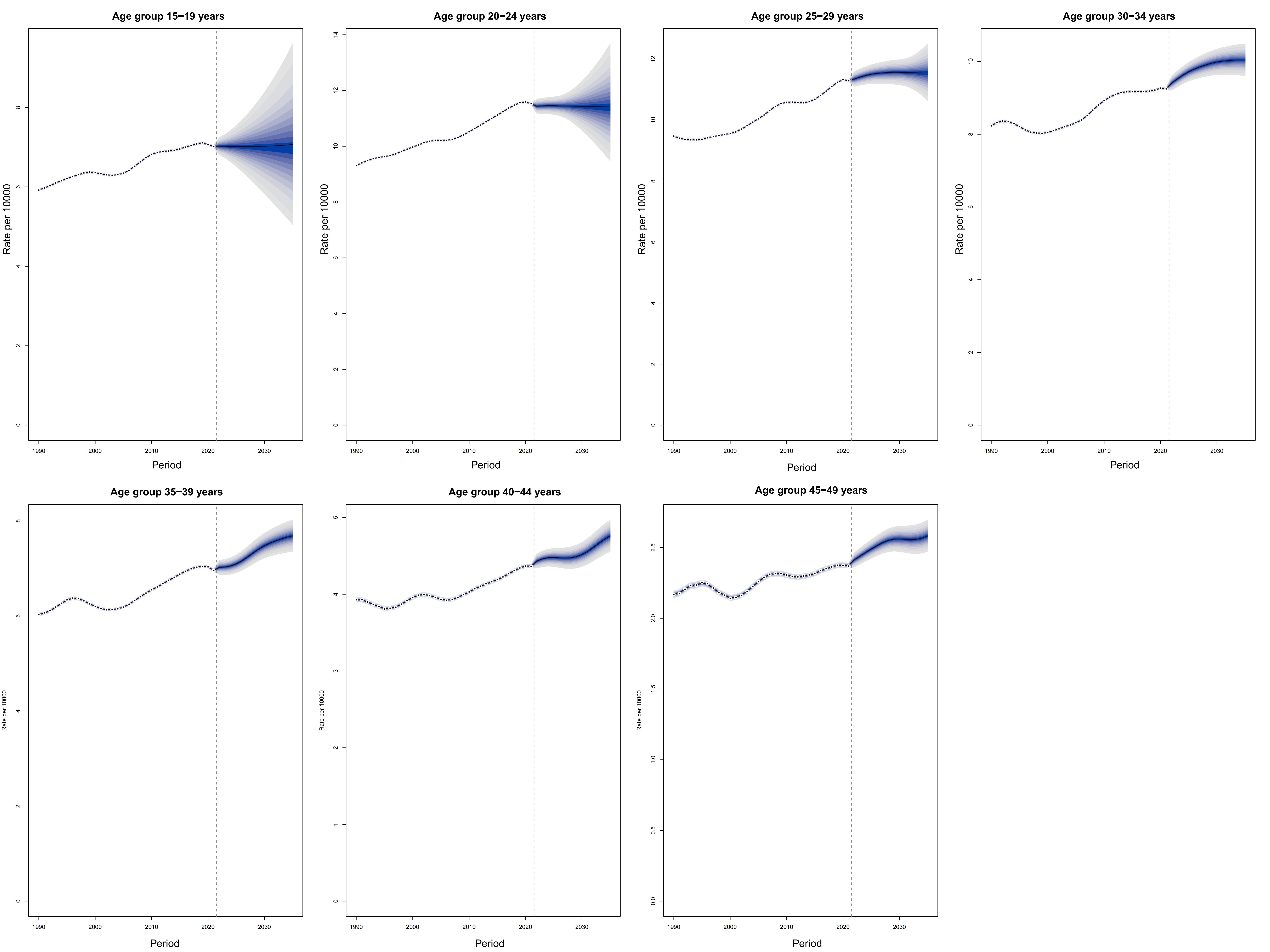

Supplement: Supplementary file 10 — Supplementary Material 10. Future predictions of the global burden of AN among WCBA at all age stages from 2022 to 2035. [file 40519_2026_1842_MOESM10_ESM.jpeg]

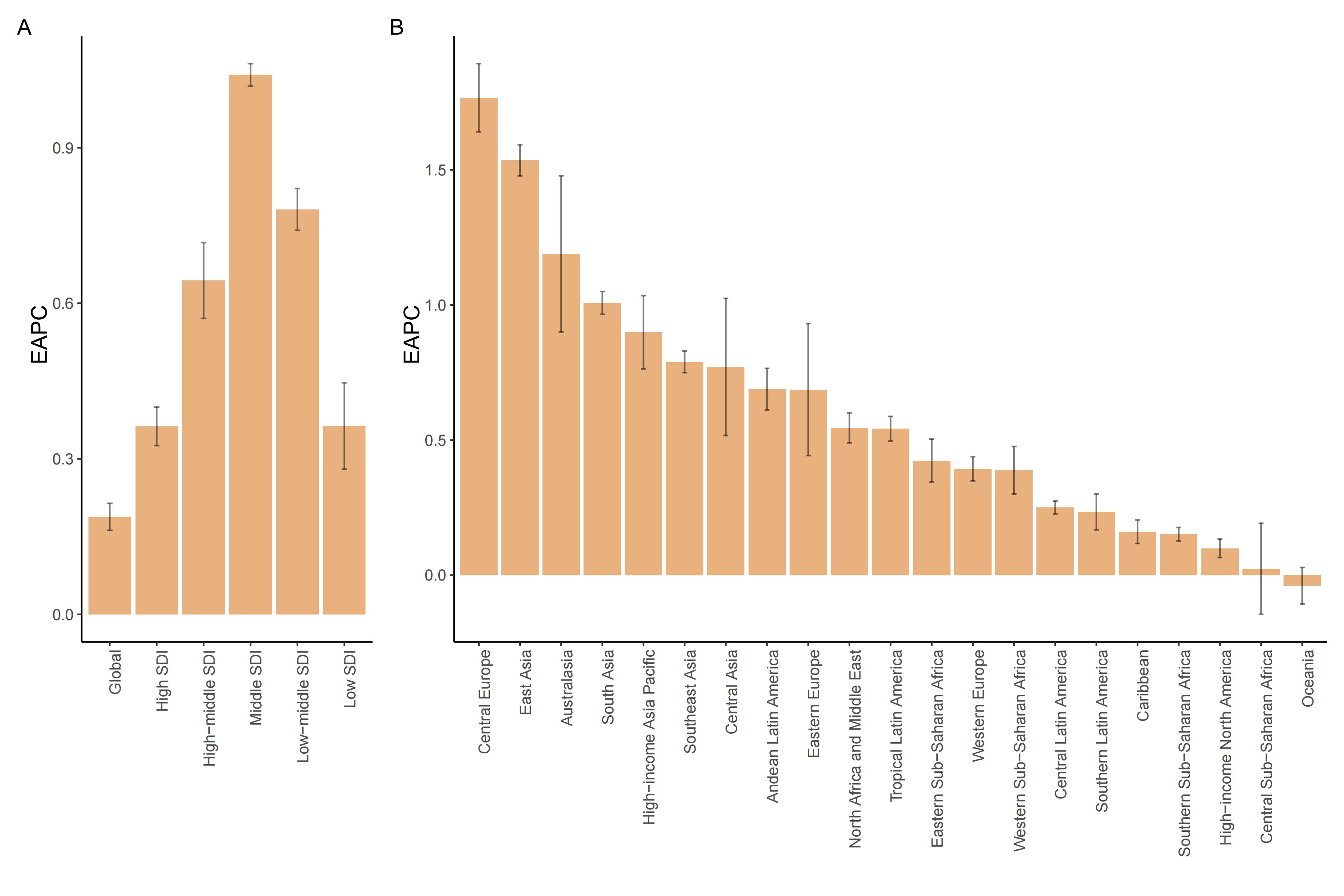

Supplement: Supplementary file 17 — Supplementary Material 17. [file 40519_2026_1842_MOESM17_ESM.jpeg]
