# Supplementary material for: Patterns and trends of eating disorders among women of childbearing age: a comprehensive analysis from 1990 to 2021 with future predictions
Source: Eat Weight Disord. 2026 Mar 23;31(1):40. doi: 10.1007/s40519-026-01842-8 (PMC13132930; doi:10.1007/s40519-026-01842-8)
Supplement: Supplementary file 11 — Supplementary Material 11. Future predictions of the global burden of BN among WCBA at all age stages from 2022 to 2035. [file 40519_2026_1842_MOESM11_ESM.docx]

Table S1. DALYs of AN cases among WCBA in 1990 and 2021 at the global and regional levels, along with their EAPCs from 1990 to 2021.

| Location | DALYs | | | | |
| --- | --- | --- | --- | --- | --- |
|  | Number of cases(95% UI) | | ASDR per 100,000 population (95% UI) | | EAPC(95% CI) |
|  | 1990 | 2021 | 1990 | 2021 | 1990-2021 |
| Global | 379603.90 (220005.72, 614121.48) | 521748.87 (303909.12, 849457.88) | 26.53 (15.41, 42.87) | 27.47 (15.98, 44.77) | 0.19 (0.16, 0.21) |
| Low SDI | 16593.39 (9263.48, 28183.85) | 43721.29 (24186.77, 73789.27) | 12.81 (7.15, 21.70) | 13.88 (7.69, 23.39) | 0.36 (0.28, 0.45) |
| Low-middle SDI | 44269.09 (24070.97, 74927.61) | 93718.68 (51970.57, 157383.46) | 14.28 (7.79, 2 4.11) | 17.71 (9.82, 29.72) | 0.78 (0.74, 0.82) |
| Middle SDI | 78138.69 (42905.80, 134091.06) | 119689.32 (66753.86, 200848.45) | 15.47 (8.51, 26.49) | 20.78 (11.58, 34.91) | 1.04 (1.02, 1.06) |
| High-middle SDI | 75774.81 (43071.61, 124032.62) | 80050.94 (45469.53, 131367.05) | 26.16 (14.88, 42.80) | 31.00 (17.55, 50.95) | 0.64 (0.57, 0.72) |
| High SDI | 164520.52 (94842.76, 263101.49) | 184185.68 (108472.00, 292386.48) | 74.63 (42.99, 119.43) | 83.81 (49.07, 133.32) | 0.36 (0.33, 0.40) |
| High-income Asia  Pacific | 33612.86 (19300.06, 54491.37) | 33814.42 (20197.13, 53180.65) | 76.92 (44.12, 124.74) | 104.22 (61.80, 164.35) | 0.90 (0.76, 1.03) |
| High-income North  America | 64013.72 (35602.96, 103528.50) | 71952.21 (41486.78, 116243.22) | 88.78 (49.36, 143.70) | 90.68 (52.14, 146.74) | 0.10 (0.07, 0.13) |
| Western Europe | 79028.98 (46863.75, 124059.88) | 78755.11 (46406.91, 126097.57) | 84.65 (50.16, 132.93) | 94.99 (55.79, 152.13) | 0.39 (0.35, 0.44) |
| Australasia | 5421.06 (2959.64, 9064.15) | 8087.19 (4525.19, 13390.85) | 102.93 (56.16, 172.25) | 121.53 (67.88, 201.48) | 1.19 (0.90, 1.48) |
| Andean Latin America | 2040.17 (1006.09, 3669.81) | 3846.22 (1908.34, 6827.55) | 18.86 (9.19, 34.10) | 22.04 (10.93, 39.13) | 0.69 (0.61, 0.77) |
| Tropical Latin America | 12898.90 (7082.49, 21662.60) | 18946.11 (10536.77, 31764.33) | 29.46 (16.17, 49.48) | 33.61 (18.69, 56.38) | 0.54 (0.50, 0.59) |
| Central Latin America | 10815.61 (5845.56, 18380.48) | 16240.64 (8840.16, 27459.08) | 22.45 (12.12, 38.15) | 24.17 (13.15, 40.87) | 0.25 (0.23, 0.27) |
| Southern Latin  America | 5687.29 (2953.16, 9809.20) | 8223.81 (4278.16, 14171.08) | 44.74 (23.19, 77.22) | 49.38 (25.79, 85.08) | 0.23 (0.17, 0.30) |
| Caribbean | 2148.25 (1120.09, 3657.47) | 2507.27 (1291.97, 4310.12) | 20.85 (10.83, 35.57) | 21.26 (10.97, 36.55) | 0.16 (0.12, 0.20) |
| Central Europe | 7073.83 (3801.46, 12017.23) | 8025.70 (4636.99, 13056.35) | 24.08 (12.95, 40.90) | 37.00 (21.24, 60.26) | 1.77 (1.64, 1.89) |
| Eastern Europe | 16432.20 (8919.78, 27510.36) | 13613.97 (7699.74, 22587.59) | 31.16 (16.92, 52.15) | 35.00 (19.76, 58.20) | 0.69 (0.44, 0.93) |
| Central Asia | 4163.34 (2156.33, 7230.96) | 5919.89 (3090.38, 10253.15) | 22.18 (11.44, 38.59) | 25.47 (13.34, 44.08) | 0.77 (0.52, 1.02) |
| North Africa and Middle East | 17219.32 (9180.11, 29260.05) | 33748.00 (18419.06, 57526.97) | 19.03 (10.14, 32.33) | 21.50 (11.75, 36.65) | 0.54 (0.49, 0.60) |
| South Asia | 37656.65 (20530.08, 63695.38) | 90040.62 (49390.31, 150479.50) | 13.21 (7.22, 22.30) | 17.64 (9.67, 29.47) | 1.01 (0.97, 1.05) |
| Southeast Asia | 17971.64 (9977.56, 30940.70) | 30312.40 (16740.79, 51032.22) | 13.30 (7.37, 22.84) | 17.18 (9.48, 28.94) | 0.79 (0.75, 0.83) |
| East Asia | 43808.00 (23646.53, 75946.42) | 48249.97 (26536.53, 82745.38) | 11.86 (6.40, 20.55) | 17.89 (9.81, 30.78) | 1.54 (1.48, 1.59) |
| Oceania | 228.83 (108.84, 410.98) | 479.97 (218.86, 886.72) | 12.85 (6.06, 23.19) | 13.11(5.95, 24.26) | -0.04 (-0.11, 0.03) |
| Western Sub-Saharan  Africa | 7759.92 (4307.28, 13219.30) | 22230.11 (12286.76, 37399.06) | 14.97 (8.31, 25.47) | 15.98 (8.85, 26.87) | 0.39 (0.30, 0.48) |
| Eastern Sub-Saharan  Africa | 6397.18 (3472.82, 10996.10) | 17013.85 (9214.81, 28865.00) | 12.39 (6.72, 21.21) | 13.64 (7.38, 23.13) | 0.42 (0.34, 0.50) |
| Central Sub-Saharan  Africa | 2106.19 (1017.85, 3689.28) | 5172.99 (2619.72, 9302.63) | 14.55 (6.94, 25.68) | 13.84 (6.94, 24.95) | 0.02 (-0.15, 0.19) |
| Southern Sub-Saharan Africa | 3119.99 (1646.83, 5348.60) | 4568.42 (2422.77, 7769.58) | 20.35 (10.73, 34.85) | 20.83 (11.05, 35.43) | 0.15 (0.13, 0.18) |

Abbreviations: DALYs, disability-adjusted life-years; AN, anorexia nervosa; WCBA, women of childbearing age; ASDR, age-standardized DALY rate; EAPC, estimated annual percentage change; UI, uncertainty interval; CI, confidence interval
