# Supplementary material for: Patterns and trends of eating disorders among women of childbearing age: a comprehensive analysis from 1990 to 2021 with future predictions
Source: Eat Weight Disord. 2026 Mar 23;31(1):40. doi: 10.1007/s40519-026-01842-8 (PMC13132930; doi:10.1007/s40519-026-01842-8)
Supplement: Supplementary file 12 — Supplementary Material 12. [file 40519_2026_1842_MOESM12_ESM.docx]

Table S2. DALYs of BN cases among WCBA in 1990 and 2021 at the global and regional levels, along with their EAPCs from 1990 to 2021.

| Location | DALYs | | | | |
| --- | --- | --- | --- | --- | --- |
|  | Number of cases(95% UI) | | ASDR per 100,000 population (95% UI) | | EAPC(95% CI) |
|  | 1990 | 2021 | 1990 | 2021 | 1990-2021 |
| Global | 927966.17 (459864.55, 1632277.14) | 1513987.52 (751032.54, 2628790.65) | 66.92 (33.36, 116.92) | 78.58 (38.87, 136.84) | 0.58 (0.54, 0.63) |
| Low SDI | 46024.61 (21539.49, 83528.32) | 132157.28 (62364.36, 238295.41) | 38.87 (18.42, 69.59) | 45.78 (21.85, 81.37) | 0.70 (0.58, 0.82) |
| Low-middle SDI | 129175.87 (61393.14, 232918.89) | 320402.64 (153661.03, 567622.79) | 44.91 (21.54, 80.04) | 61.80 (29.73, 109.17) | 1.18 (1.11, 1.25) |
| Middle SDI | 228611.20 (109106.84, 410225.67) | 430817.54 (213434.49, 758069.75) | 48.12 (23.17, 85.50) | 71.73 (35.31, 126.95) | 1.39 (1.34, 1.44) |
| High-middle SDI | 194919.46 (98002.58, 338569.96) | 253989.14 (128054.49, 434325.72) | 68.19 (34.37, 118.21) | 89.98 (44.77, 156.00) | 1.06 (0.95, 1.17) |
| High SDI | 328345.53 (165892.48, 570021.71) | 375412.61 (193694.23, 641539.95) | 146.26 (73.55, 255.33) | 164.84 (84.26, 285.10) | 0.26 (0.19, 0.33) |
| High-income Asia  Pacific | 51115.13 (24859.46, 90741.23) | 48210.10 (23863.02, 83487.01) | 115.72 (56.22, 205.75) | 142.69 (70.10, 250.38) | 0.62 (0.57, 0.67) |
| High-income North  America | 138326.16 (68596.34, 240579.49) | 143048.46 (71552.21, 251459.75) | 187.55 (92.31, 328.68) | 177.51 (88.21, 314.27) | -0.43 (-0.63, -0.22) |
| Western Europe | 171399.46 (88742.95, 290019.10) | 183592.61 (94822.66, 312612.96) | 181.24 (93.70, 307.55) | 210.24 (107.72, 361.16) | 0.54 (0.48, 0.59) |
| Australasia | 15974.66 (7849.28, 28224.85) | 28230.53 (16118.41, 45520.27) | 299.71 (146.80, 530.70) | 408.76 (232.60, 661.97) | 1.04 (0.94, 1.14) |
| Andean Latin America | 13931.64 (6810.88, 25005.67) | 30906.46 (15317.49, 54535.44) | 137.01 (67.38, 244.57) | 174.12 (86.14, 307.68) | 0.82 (0.77, 0.88) |
| Tropical Latin America | 36185.37 (17319.31, 65134.29) | 58931.51 (29190.28, 103709.70) | 85.49 (41.08, 152.97) | 100.22 (49.42, 177.49) | 0.65 (0.59, 0.71) |
| Central Latin America | 50332.80 (24784.54, 88919.94) | 79435.33 (39505.15, 139803.24) | 111.38 (55.17, 195.23) | 117.00 (58.15, 206.21) | 0.20 (0.17, 0.22) |
| Southern Latin  America | 15782.91 (7621.04, 28109.03) | 26081.06 (12652.47, 46375.23) | 125.65 (60.79, 223.47) | 152.62 (73.73, 272.78) | 0.65 (0.60, 0.71) |
| Caribbean | 9716.53 (4742.63, 17052.80) | 12475.00 (6186.02, 21944.33) | 98.57 (48.37, 172.13) | 104.05 (51.53, 183.35) | 0.35 (0.30, 0.40) |
| Central Europe | 16971.18 (8061.74, 30154.77) | 17370.30 (8479.33, 30784.15) | 56.21 (26.56, 100.46) | 74.16 (35.62, 133.59) | 1.13 (1.04, 1.22) |
| Eastern Europe | 35856.63 (17244.15, 63541.94) | 30601.38 (14982.08, 53321.07) | 64.55 (30.71, 115.52) | 69.78 (33.50, 124.21) | 0.64 (0.37, 0.91) |
| Central Asia | 8981.80 (4226.04, 16090.80) | 13916.70 (6643.48, 24703.38) | 49.84 (23.51, 88.84) | 57.32 (27.09, 102.57) | 0.88 (0.56, 1.19) |
| North Africa and Middle East | 69191.65 (33442.39, 124310.63) | 160379.62 (79435.75, 282684.28) | 83.99 (41.11, 148.90) | 100.72 (49.78, 177.90) | 0.87 (0.79, 0.95) |
| South Asia | 106845.77 (49957.08, 194738.12) | 310780.81 (148890.66, 551230.03) | 40.02 (18.88, 72.24) | 61.61 (29.59, 109.09) | 1.54 (1.47, 1.61) |
| Southeast Asia | 40653.38 (18765.57, 74100.20) | 84530.32 (40218.36, 151391.40) | 31.93 (14.84, 57.73) | 46.73 (22.18, 83.98) | 1.23 (1.15, 1.30) |
| East Asia | 92510.21 (42962.78, 168339.14) | 136967.33 (64767.69, 241002.01) | 26.36 (12.36, 47.66) | 44.70 (20.69, 80.22) | 1.78 (1.65, 1.92) |
| Oceania | 546.42 (237.32, 1027.46) | 1264.63 (563.01, 2343.36) | 33.31 (14.63, 62.05) | 35.42 (15.80, 65.52) | 0.10 (0.03, 0.16) |
| Western Sub-Saharan  Africa | 21792.18 (10253.85, 39608.17) | 67692.25 (31870.95, 121748.56) | 46.56 (22.22, 83.15) | 53.48 (25.51, 94.74) | 0.70 (0.57, 0.83) |
| Eastern Sub-Saharan  Africa | 16864.23 (7781.91, 30945.80) | 49301.89 (22906.74, 88646.91) | 36.39 (17.04, 65.60) | 43.48 (20.43, 77.22) | 0.73 (0.62, 0.84) |
| Central Sub-Saharan  Africa | 5690.50 (2517.17, 10629.89) | 14617.87 (6501.03, 26429.11) | 43.30 (19.30, 80.00) | 42.65 (19.22, 76.23) | 0.20 (-0.02, 0.42) |
| Southern Sub-Saharan Africa | 9297.54 (4435.74, 16636.31) | 15653.38 (7627.21, 27445.53) | 65.86 (31.82, 116.52) | 70.75 (34.38, 124.24) | 0.36 (0.32, 0.40) |

Abbreviations: DALYs, disability-adjusted life-years; BN, bulimia nervosa; WCBA, women of childbearing age; ASDR, age-standardized DALY rate; EAPC, estimated annual percentage change; UI, uncertainty interval; CI, confidence interval
