# Supplementary material for: Patterns and trends of eating disorders among women of childbearing age: a comprehensive analysis from 1990 to 2021 with future predictions
Source: Eat Weight Disord. 2026 Mar 23;31(1):40. doi: 10.1007/s40519-026-01842-8 (PMC13132930; doi:10.1007/s40519-026-01842-8)
Supplement: Supplementary file 13 — Supplementary Material 13. [file 40519_2026_1842_MOESM13_ESM.docx]

Table S3. DALYs of ED cases among WCBA in 1990 and 2021 at the national level, along with their EAPCs from 1990 to 2021.

| Location | DALYs | | | | |
| --- | --- | --- | --- | --- | --- |
|  | Number of cases(95% UI) | | ASDR per 100,000 population (95% UI) | | EAPC(95% CI) |
|  | 1990 | 2021 | 1990 | 2021 | 1990-2021 |
| Afghanistan | 1517.14 (752.50, 2686.88) | 5168.88 (2536.78, 8976.52) | 65.94 (32.99, 115.58) | 65.82 (32.43, 114.05) | 0.39 (0.11, 0.67) |
| Albania | 559.76 (289.17, 946.00) | 497.81 (260.67, 841.09) | 61.77 (31.75, 104.41) | 83.41 (43.67, 141.03) | 1.31 (1.20, 1.43) |
| Algeria | 7088.53 (3647.66, 12332.53) | 13133.17 (6730.06, 22792.84) | 111.06 (57.07, 192.39) | 120.04 (61.46, 208.55) | 0.50 (0.41, 0.60) |
| American Samoa | 10.32 (5.24, 17.70) | 7.94 (4.04, 13.67) | 78.78 (39.88, 135.42) | 69.84 (35.66, 120.08) | -0.30 (-0.33, -0.27) |
| Andorra | 46.57 (26.51, 75.30) | 56.14 (31.61, 89.77) | 309.09 (175.83, 501.88) | 327.23 (184.96, 524.57) | 0.29 (0.20, 0.39) |
| Angola | 1651.66 (830.64, 2858.54) | 6710.39 (3499.32, 11593.39) | 66.20 (33.16, 114.50) | 81.96 (42.53, 141.43) | 1.07 (0.91, 1.22) |
| Antigua and Barbuda | 26.14 (13.30, 44.44) | 39.89 (20.45, 67.92) | 150.75 (76.74, 256.34) | 173.06 (88.64, 294.90) | 0.44 (0.37, 0.51) |
| Argentina | 14225.23 (7603.98, 23829.05) | 22971.13 (12299.41, 38339.39) | 176.48 (94.34, 295.47) | 197.88 (105.97, 330.43) | 0.37 (0.31, 0.43) |
| Armenia | 607.71 (314.36, 1007.14) | 553.15 (291.60, 930.30) | 65.71 (33.64, 109.52) | 80.18 (42.33, 134.72) | 1.18 (0.90, 1.46) |
| Australia | 18085.17 (10128.99, 29555.34) | 32163.90 (19837.36, 48758.96) | 410.12 (229.36, 670.65) | 564.73 (348.93, 856.23) | 1.23 (1.10, 1.36) |
| Austria | 5318.34 (3010.41, 8598.20) | 6348.77 (3568.61, 10200.23) | 270.10 (153.02, 437.99) | 342.46 (191.94, 551.42) | 0.85 (0.79, 0.91) |
| Azerbaijan | 1608.69 (837.62, 2731.69) | 2487.75 (1292.71, 4134.55) | 77.31 (39.92, 131.39) | 93.25 (48.35, 155.34) | 1.25 (0.78, 1.71) |
| Bahamas | 148.90 (77.49, 251.11) | 195.36 (100.63, 330.68) | 190.42 (98.92, 321.13) | 187.38 (96.66, 316.89) | 0.08 (-0.01, 0.16) |
| Bahrain | 187.09 (97.82, 317.35) | 542.86 (289.23, 934.92) | 147.02 (76.73, 249.77) | 168.96 (89.89, 291.64) | 0.58 (0.52, 0.63) |
| Bangladesh | 13993.67 (7185.50, 24341.01) | 33240.70 (16280.99, 57011.34) | 51.37 (26.24, 89.14) | 70.67 (34.61, 121.15) | 1.07 (0.96, 1.18) |
| Barbados | 109.44 (55.35, 182.49) | 99.45 (51.32, 169.81) | 153.32 (77.40, 256.04) | 150.12 (77.71, 256.28) | -0.08 (-0.21, 0.05) |
| Belarus | 2157.16 (1138.41, 3665.91) | 1867.79 (992.34, 3091.85) | 84.87 (44.74, 144.64) | 99.76 (53.30, 165.03) | 0.90 (0.68, 1.13) |
| Belgium | 5466.83 (3104.89, 8833.43) | 6392.60 (3702.56, 10168.80) | 227.70 (129.10, 368.83) | 275.55 (159.21, 439.64) | 0.70 (0.65, 0.74) |
| Belize | 50.64 (25.81, 87.16) | 155.36 (79.14, 267.36) | 107.28 (54.50, 183.53) | 123.90 (63.04, 213.24) | 0.45 (0.37, 0.53) |
| Benin | 657.92 (328.27, 1149.87) | 2158.67 (1075.73, 3729.75) | 54.58 (27.17, 95.32) | 61.29 (30.43, 105.89) | 0.47 (0.43, 0.51) |
| Bermuda | 34.64 (18.32, 59.20) | 27.97 (14.53, 47.23) | 203.04 (107.39, 347.74) | 235.75 (122.69, 399.31) | 0.64 (0.56, 0.73) |
| Bhutan | 93.07 (46.49, 161.07) | 194.26 (98.90, 334.13) | 58.77 (29.25, 101.43) | 91.13 (46.32, 156.89) | 1.50 (1.48, 1.53) |
| Bolivia | 2144.35 (1069.64, 3739.33) | 5428.75 (2752.65, 9372.01) | 131.16 (65.42, 228.54) | 169.13 (85.74, 292.16) | 0.91 (0.88, 0.95) |
| Bosnia and Herzegovina | 580.35 (291.57, 987.55) | 565.80 (289.02, 950.27) | 48.99 (24.55, 83.46) | 85.44 (43.80, 143.66) | 2.17 (1.96, 2.39) |
| Botswana | 289.60 (148.93, 496.80) | 754.40 (394.18, 1287.31) | 81.92 (41.92, 140.38) | 109.36 (57.01, 186.81) | 0.98 (0.95, 1.01) |
| Brazil | 48010.21 (26186.89, 79572.53) | 75360.98 (41578.96, 122567.21) | 115.18 (62.90, 190.46) | 134.03 (73.94, 218.28) | 0.63 (0.57, 0.68) |
| Brunei Darussalam | 214.14 (122.52, 346.25) | 335.35 (192.64, 537.82) | 289.65 (165.50, 468.91) | 274.36 (157.37, 440.19) | 0.02 (-0.05, 0.08) |
| Bulgaria | 1595.29 (830.82, 2752.88) | 1212.16 (645.98, 2019.71) | 81.42 (42.53, 140.31) | 98.19 (52.62, 163.79) | 0.83 (0.66, 1.00) |
| Burkina Faso | 1083.44 (533.56, 1874.57) | 3346.22 (1655.43, 5769.47) | 48.02 (23.52, 83.07) | 56.01 (27.52, 96.64) | 0.53 (0.49, 0.57) |
| Burundi | 662.82 (326.90, 1147.68) | 1472.20 (696.81, 2580.71) | 47.96 (23.53, 83.30) | 42.91 (20.17, 75.33) | -0.34 (-0.39, -0.28) |
| Cabo Verde | 53.03 (26.98, 91.55) | 129.94 (67.05, 223.38) | 59.55 (30.08, 102.89) | 83.74 (43.04, 144.06) | 1.29 (1.22, 1.37) |
| Cambodia | 927.72 (457.20, 1628.82) | 2260.60 (1108.45, 3981.85) | 34.14 (16.64, 60.09) | 48.71 (23.84, 85.84) | 1.28 (1.18, 1.39) |
| Cameroon | 1723.30 (869.94, 2944.27) | 5653.12 (2921.91, 9781.79) | 66.31 (33.33, 112.91) | 66.96 (34.37, 115.78) | 0.17 (0.08, 0.26) |
| Canada | 18140.75 (9802.79, 29853.51) | 20185.47 (11267.70, 32526.74) | 252.01 (135.58, 415.93) | 261.66 (145.77, 422.06) | 0.21 (0.18, 0.23) |
| Central African Republic | 336.59 (164.20, 590.91) | 642.91 (311.11, 1137.05) | 48.17 (23.39, 84.57) | 43.24 (20.79, 76.43) | -0.34 (-0.37, -0.31) |
| Chad | 702.83 (348.73, 1199.17) | 2433.78 (1209.62, 4169.21) | 48.80 (24.14, 83.01) | 57.38 (28.39, 98.11) | 0.72 (0.62, 0.81) |
| Chile | 5984.36 (3217.10, 9952.24) | 9671.81 (5266.21, 16186.51) | 157.85 (84.82, 263) | 211.50 (115.20, 354.48) | 0.95 (0.91, 0.99) |
| China | 130126.26 (70295.16, 214951.85) | 178273.68 (97772.81, 293287.05) | 37.71 (20.43, 62.22) | 62.66 (34.15, 103.60) | 1.76 (1.66, 1.87) |
| Colombia | 10619.94 (5497.41, 18235.46) | 18086.49 (9287.36, 30752.68) | 112.17 (57.94, 192.64) | 137.31 (70.56, 233.67) | 0.69 (0.61, 0.78) |
| Comoros | 73.95 (37.20, 126.34) | 125.44 (63.57, 216.06) | 64.56 (32.32, 110.10) | 62.15 (31.40, 107.02) | -0.08 (-0.13, -0.02) |
| Congo | 438.63 (227.72, 743.60) | 1141.77 (589.29, 1960.19) | 70.96 (36.68, 120.26) | 77.54 (40.00, 132.95) | 0.48 (0.37, 0.58) |
| Cook Islands | 3.38 (1.69, 5.86) | 3.74 (1.92, 6.53) | 69.52 (34.73, 120.28) | 88.96 (45.83, 155.32) | 0.69 (0.66, 0.73) |
| Costa Rica | 1032.20 (535.84, 1758.45) | 1878.49 (999.79, 3227.19) | 122.08 (63.30, 208.00) | 146.56 (78.05, 252.15) | 0.63 (0.62, 0.64) |
| Cote d'Ivoire | 2029.77 (1062.99, 3482.97) | 5003.19 (2555.60, 8476.93) | 66.65 (34.64, 114.15) | 70.39 (35.92, 119.42) | 0.12 (-0.00, 0.25) |
| Croatia | 1034.18 (541.86, 1730.27) | 907.75 (503.78, 1483.75) | 88.82 (46.60, 148.86) | 110.83 (61.55, 181.19) | 1.07 (0.96, 1.18) |
| Cuba | 3973.80 (2027.21, 6770.96) | 2901.86 (1473.79, 5001.17) | 123.19 (62.87, 210.03) | 125.70 (64.08, 216.29) | 0.42 (0.24, 0.60) |
| Cyprus | 427.80 (240.26, 688.65) | 826.36 (458.29, 1341.32) | 217.71 (122.24, 350.82) | 253.95 (140.10, 413.51) | 0.52 (0.42, 0.62) |
| Czechia | 2364.84 (1275.86, 3966.00) | 2405.11 (1311.12, 3966.86) | 97.98 (53.08, 164.29) | 122.72 (67.19, 203.09) | 0.93 (0.86, 0.99) |
| Democratic People's Republic of Korea | 2306.13 (1101.42, 4004.55) | 2278.12 (1091.93, 4010.04) | 39.34 (18.75, 68.31) | 36.21 (17.44, 63.59) | -0.19 (-0.29, -0.09) |
| Democratic Republic of the Congo | 5056.28 (2479.60, 8811.55) | 10271.69 (4909.91, 17552.59) | 54.27 (26.42, 94.29) | 44.54 (21.23, 76.28) | -0.52 (-0.82, -0.23) |
| Denmark | 3224.39 (1846.54, 5191.88) | 3493.22 (2002.96, 5670.35) | 257.14 (147.32, 414.53) | 289.28 (165.85, 469.73) | 0.42 (0.35, 0.48) |
| Djibouti | 75.92 (37.51, 132.42) | 231.03 (117.26, 392.42) | 70.71 (34.80, 123.15) | 71.22 (36.09, 121.22) | 0.05 (-0.09, 0.19) |
| Dominica | 21.96 (11.10, 37.50) | 21.99 (11.28, 37.09) | 119.89 (60.60, 204.27) | 134.45 (68.94, 226.74) | 0.42 (0.38, 0.46) |
| Dominican Republic | 2342.58 (1183.52, 3959.02) | 4556.00 (2388.87, 7835.59) | 112.24 (56.61, 189.95) | 153.80 (80.58, 264.54) | 1.10 (1.08, 1.13) |
| Ecuador | 4628.43 (2343.05, 8063.22) | 9376.77 (4784.71, 15925.88) | 169.39 (85.77, 294.14) | 194.84 (99.33, 331.30) | 0.65 (0.57, 0.74) |
| Egypt | 11994.95 (6183.10, 20779.04) | 30597.47 (15793.96, 51990.15) | 86.74 (44.65, 149.99) | 114.84 (59.23, 195.02) | 1.01 (0.97, 1.05) |
| El Salvador | 1380.41 (697.03, 2369.99) | 2138.28 (1088.30, 3655.99) | 96.57 (48.67, 165.65) | 117.56 (59.84, 201.16) | 0.66 (0.62, 0.70) |
| Equatorial Guinea | 57.75 (28.17, 100.14) | 471.83 (246.31, 791.41) | 54.50 (26.55, 94.34) | 121.11 (63.15, 202.77) | 3.62 (2.98, 4.26) |
| Eritrea | 407.38 (200.92, 716.92) | 980.52 (492.33, 1715.99) | 47.74 (23.36, 84.08) | 56.02 (28.04, 98.05) | 0.34 (0.17, 0.51) |
| Estonia | 314.94 (164.64, 527.48) | 287.98 (154.74, 487.06) | 86.01 (45.01, 144.14) | 115.73 (62.60, 196.25) | 1.21 (1.12, 1.31) |
| Eswatini | 166.72 (85.68, 284.20) | 303.98 (160.72, 515.00) | 77.99 (39.81, 133.13) | 90.39 (47.68, 153.03) | 0.41 (0.38, 0.45) |
| Ethiopia | 5290.00 (2782.77, 8941.96) | 17000.51 (8963.01, 28550.21) | 42.50 (22.25, 71.65) | 56.22 (29.70, 94.27) | 1.09 (0.86, 1.31) |
| Fiji | 110.08 (55.35, 187.80) | 141.01 (70.76, 242.45) | 53.22 (26.65, 90.90) | 61.83 (31.02, 106.32) | 0.44 (0.40, 0.48) |
| Finland | 3371.55 (1973.25, 5379.47) | 3375.39 (1960.23, 5358.41) | 285.10 (166.93, 455.51) | 315.07 (182.87, 500.84) | 0.39 (0.29, 0.48) |
| France | 38705.39 (22136.26, 62438.81) | 41390.95 (23656.43, 66897.76) | 270.61 (154.57, 436.95) | 311.66 (177.91, 503.93) | 0.47 (0.44, 0.49) |
| Gabon | 255.78 (131.90, 433.18) | 552.27 (285.76, 932.66) | 104.98 (54.18, 177.42) | 106.94 (55.41, 180.36) | 0.08 (0.06, 0.09) |
| Gambia | 155.14 (78.85, 263.68) | 395.63 (199.64, 675.85) | 61.00 (30.81, 103.83) | 58.94 (29.61, 100.76) | -0.08 (-0.12, -0.05) |
| Georgia | 1142.99 (598.87, 1926.10) | 598.09 (308.78, 1009.92) | 81.65 (42.66, 137.75) | 82.77 (42.84, 140.10) | 0.62 (0.19, 1.04) |
| Germany | 45980.39 (27197.97, 71463.04) | 43739.08 (25976.25, 68739.81) | 239.19 (141.84, 372.19) | 274.84 (162.94, 433.09) | 0.45 (0.43, 0.47) |
| Ghana | 2295.48 (1149.82, 3957.27) | 7338.34 (3785.53, 12455.62) | 59.66 (29.80, 102.71) | 76.23 (39.21, 129.46) | 0.83 (0.75, 0.91) |
| Greece | 6013.24 (3432.81, 9692.89) | 5225.44 (3029.42, 8362.43) | 243.47 (139.13, 392.44) | 275.52 (159.70, 441.30) | 0.52 (0.39, 0.65) |
| Greenland | 47.01 (26.03, 77.15) | 40.67 (22.86, 66.50) | 295.69 (164.21, 486.35) | 317.68 (178.02, 521.08) | 0.43 (0.35, 0.51) |
| Grenada | 24.18 (12.52, 41.76) | 37.96 (19.85, 64.23) | 114.03 (58.73, 196.64) | 146.73 (76.74, 248.53) | 0.92 (0.85, 0.99) |
| Guam | 37.73 (19.35, 63.96) | 36.28 (18.69, 61.30) | 102.02 (52.28, 173.15) | 103.12 (53.25, 174.11) | 0.15 (0.11, 0.19) |
| Guatemala | 2037.66 (1029.62, 3494.28) | 5411.76 (2815.38, 9045.50) | 102.35 (51.55, 175.58) | 116.15 (60.32, 194.20) | 0.47 (0.45, 0.49) |
| Guinea | 814.57 (411.50, 1409.51) | 2114.59 (1065.91, 3663.51) | 56.21 (28.25, 97.26) | 58.88 (29.46, 102.03) | 0.05 (-0.06, 0.15) |
| Guinea-Bissau | 133.10 (66.78, 230.40) | 309.18 (151.53, 535.03) | 52.42 (26.11, 90.92) | 54.57 (26.70, 94.46) | 0.03 (-0.02, 0.09) |
| Guyana | 215.95 (107.05, 374.33) | 270.05 (136.60, 462.32) | 95.84 (47.50, 165.62) | 127.97 (64.83, 219.02) | 0.89 (0.85, 0.93) |
| Haiti | 1351.06 (682.40, 2337.21) | 2794.29 (1422.21, 4785.95) | 81.23 (40.94, 140.53) | 76.61 (38.92, 131.26) | -0.09 (-0.12, -0.05) |
| Honduras | 1071.09 (533.09, 1850.58) | 3050.83 (1530.46, 5267.34) | 92.79 (46.05, 159.94) | 101.12 (50.70, 174.34) | 0.27 (0.24, 0.30) |
| Hungary | 2253.80 (1174.03, 3848.48) | 2277.70 (1245.73, 3799.71) | 94.64 (49.41, 161.90) | 122.16 (67.10, 203.96) | 0.99 (0.93, 1.05) |
| Iceland | 167.57 (93.25, 272.08) | 221.68 (124.89, 359.74) | 253.10 (140.85, 411.22) | 288.78 (162.77, 469.84) | 0.49 (0.44, 0.54) |
| India | 112073.21 (60089.07, 186866.20) | 312189.33 (170338.47, 513536.47) | 52.41 (28.16, 87.18) | 81.00 (44.23, 133.22) | 1.56 (1.49, 1.62) |
| Indonesia | 24026.33 (13130.70, 39970.64) | 47676.81 (25927.83, 78544.07) | 46.56 (25.42, 77.30) | 64.93 (35.28, 107.05) | 1.03 (0.94, 1.12) |
| Iran (Islamic Republic of) | 16571.78 (8734.51, 28047.33) | 32036.22 (17360.72, 52760.74) | 119.67 (63.40, 201.13) | 147.34 (79.46, 243.86) | 0.93 (0.81, 1.04) |
| Iraq | 4681.77 (2364.17, 8175.13) | 13551.25 (6858.45, 23050.41) | 105.88 (53.57, 183.23) | 124.32 (62.86, 211.44) | 1.01 (0.80, 1.22) |
| Ireland | 1735.04 (955.77, 2856.38) | 3060.47 (1700.55, 4930.82) | 195.64 (107.68, 321.67) | 288.54 (160.32, 464.58) | 1.26 (1.13, 1.40) |
| Israel | 2086.87 (1129.32, 3414.32) | 4642.48 (2558.73, 7728.99) | 169.83 (91.84, 277.96) | 214.26 (118.18, 356.59) | 0.82 (0.74, 0.91) |
| Italy | 52520.58 (30147.57, 84537.12) | 41186.40 (23473.71, 66377.23) | 373.10 (214.19, 601.03) | 380.31 (216.95, 615.44) | 0.08 (0.03, 0.12) |
| Jamaica | 766.31 (392.34, 1320.07) | 984.99 (493.79, 1692.48) | 116.44 (59.45, 200.25) | 124.96 (62.61, 214.76) | 0.25 (0.21, 0.29) |
| Japan | 63551.83 (37199.85, 100545.24) | 56895.40 (34328.34, 88583.43) | 215.34 (125.95, 340.79) | 264.09 (158.63, 413.27) | 0.59 (0.49, 0.68) |
| Jordan | 874.96 (437.73, 1518.00) | 3431.90 (1719.67, 5876.90) | 94.61 (47.53, 163.18) | 107.70 (53.95, 184.08) | 0.74 (0.61, 0.86) |
| Kazakhstan | 3618.10 (1911.42, 6048.60) | 4796.39 (2590.07, 7997.04) | 83.72 (44.09, 140.19) | 105.59 (56.85, 176.74) | 1.18 (0.91, 1.45) |
| Kenya | 3540.20 (1894.94, 5958.24) | 9681.93 (5181.34, 16124.61) | 60.89 (32.75, 101.97) | 67.68 (36.25, 112.22) | 0.34 (0.25, 0.43) |
| Kiribati | 8.05 (3.85, 13.82) | 13.14 (6.50, 23.07) | 39.72 (18.82, 68.40) | 39.72 (19.58, 69.79) | -0.02 (-0.06, 0.02) |
| Kuwait | 794.17 (423.79, 1327.94) | 2675.02 (1415.08, 4452.73) | 176.04 (93.89, 294.56) | 196.60 (104.00, 329.33) | 0.51 (0.39, 0.64) |
| Kyrgyzstan | 759.41 (391.83, 1294.38) | 1100.80 (556.14, 1882.14) | 65.95 (33.74, 112.75) | 62.67 (31.57, 107.31) | -0.01 (-0.33, 0.31) |
| Lao People's Democratic Republic | 387.00 (184.17, 689.72) | 1189.53 (592.66, 2045.91) | 36.95 (17.45, 65.72) | 57.71 (28.66, 99.34) | 1.53 (1.44, 1.62) |
| Latvia | 553.35 (297.23, 927.09) | 384.85 (204.63, 636.29) | 88.80 (47.71, 148.83) | 111.50 (59.37, 184.69) | 1.09 (0.92, 1.27) |
| Lebanon | 811.42 (411.53, 1404.33) | 1705.07 (889.37, 2903.71) | 104.05 (52.90, 179.91) | 118.07 (61.18, 201.09) | 0.68 (0.59, 0.77) |
| Lesotho | 208.55 (104.65, 359.91) | 358.09 (182.35, 614.69) | 52.46 (26.28, 90.38) | 65.86 (33.33, 113.10) | 0.76 (0.75, 0.78) |
| Liberia | 314.41 (153.32, 547.20) | 693.72 (347.90, 1224.61) | 51.30 (24.81, 89.34) | 47.13 (23.52, 82.95) | 0.15 (-0.04, 0.35) |
| Libya | 1525.99 (794.88, 2567.96) | 2022.61 (1052.06, 3490.34) | 152.77 (79.76, 256.59) | 105.34 (54.88, 181.96) | -0.75 (-1.01, -0.49) |
| Lithuania | 787.91 (411.26, 1333.63) | 601.09 (326.54, 998.28) | 86.71 (45.23, 146.75) | 114.06 (61.95, 190.03) | 1.26 (1.10, 1.41) |
| Luxembourg | 275.24 (154.31, 443.29) | 534.26 (303.57, 862.05) | 287.22 (160.94, 463.51) | 369.00 (209.63, 597.20) | 0.83 (0.75, 0.91) |
| Madagascar | 1588.72 (790.27, 2752.04) | 4204.17 (2066.54, 7157.53) | 53.63 (26.53, 92.87) | 53.97 (26.54, 91.69) | 0.12 (0.06, 0.18) |
| Malawi | 1155.31 (565.76, 2033.46) | 2708.96 (1332.17, 4653.18) | 46.20 (22.48, 81.49) | 49.26 (23.86, 84.77) | 0.29 (0.25, 0.33) |
| Malaysia | 2988.91 (1502.29, 5143.35) | 7462.37 (3884.23, 12760.10) | 62.57 (31.31, 107.82) | 86.87 (45.17, 148.68) | 1.05 (1.01, 1.08) |
| Maldives | 28.02 (14.04, 49.15) | 83.37 (42.60, 142.37) | 52.61 (26.15, 92.12) | 73.81 (37.63, 126.57) | 1.19 (1.15, 1.24) |
| Mali | 970.29 (466.68, 1699.15) | 3524.39 (1776.67, 6147.89) | 46.88 (22.52, 82.11) | 58.86 (29.44, 102.34) | 0.78 (0.75, 0.81) |
| Malta | 172.89 (94.77, 290.21) | 225.30 (125.47, 370.65) | 193.03 (105.91, 325.42) | 260.67 (145.38, 429.26) | 0.92 (0.85, 0.98) |
| Marshall Islands | 4.75 (2.32, 8.37) | 6.98 (3.45, 12.31) | 43.85 (21.17, 77.46) | 46.41 (22.90, 81.90) | 0.09 (0.06, 0.12) |
| Mauritania | 316.75 (161.73, 539.73) | 805.21 (414.03, 1383.88) | 62.04 (31.55, 105.63) | 70.00 (35.82, 120.21) | 0.47 (0.41, 0.54) |
| Mauritius | 187.58 (93.54, 319.16) | 241.21 (121.87, 414.70) | 59.58 (29.62, 101.51) | 79.61 (40.38, 136.91) | 0.85 (0.80, 0.90) |
| Mexico | 35834.80 (19117.31, 60493.28) | 52875.59 (28265.41, 88941.82) | 149.86 (80.21, 252.00) | 153.28 (81.95, 258.03) | 0.06 (0.04, 0.08) |
| Micronesia (Federated States of) | 10.97 (5.53, 19.17) | 12.34 (5.96, 21.63) | 43.25 (21.55, 75.76) | 45.62 (22.02, 79.90) | 0.16 (0.13, 0.18) |
| Monaco | 29.02 (16.12, 46.50) | 34.50 (19.93, 55.72) | 454.70 (252.92, 730.46) | 537.86 (311.54, 869.95) | 0.56 (0.54, 0.58) |
| Mongolia | 342.68 (172.14, 577.04) | 692.56 (359.20, 1153.77) | 59.84 (29.83, 100.86) | 84.81 (43.86, 141.47) | 1.45 (1.26, 1.65) |
| Montenegro | 135.13 (70.40, 227.44) | 127.12 (67.62, 213.48) | 85.75 (44.65, 144.43) | 95.27 (50.87, 159.93) | 0.74 (0.55, 0.94) |
| Morocco | 5637.64 (2770.64, 9778.38) | 9751.06 (4897.15, 16837.59) | 83.26 (40.96, 144.42) | 101.83 (51.14, 175.91) | 0.70 (0.63, 0.78) |
| Mozambique | 1266.84 (595.01, 2258.10) | 4122.47 (2065.24, 7186.11) | 37.54 (17.56, 66.97) | 49.51 (24.70, 86.29) | 1.12 (1.05, 1.19) |
| Myanmar | 3705.86 (1782.36, 6547.87) | 8518.88 (4320.47, 14621.27) | 32.59 (15.53, 57.72) | 56.36 (28.58, 96.65) | 2.03 (1.88, 2.19) |
| Namibia | 302.78 (152.79, 519.00) | 673.52 (349.88, 1153.58) | 81.42 (41.13, 138.94) | 97.25 (50.47, 166.47) | 0.75 (0.66, 0.84) |
| Nauru | 1.95 (1.00, 3.34) | 1.98 (1.00, 3.40) | 75.45 (38.66, 129.22) | 66.37 (33.46, 114.15) | -0.39 (-0.82, 0.04) |
| Nepal | 2363.28 (1147.05, 4087.17) | 6083.66 (3043.76, 10681.51) | 48.17 (23.36, 83.22) | 64.10 (32.04, 112.44) | 0.92 (0.86, 0.98) |
| Netherlands | 6147.71 (3783.29, 9388.19) | 8093.82 (4816.10, 12573.76) | 156.37 (96.40, 238.56) | 231.56 (137.99, 359.69) | 1.65 (1.52, 1.78) |
| New Zealand | 3310.55 (1823.57, 5422.66) | 4153.82 (2296.73, 6847.18) | 365.64 (201.33, 599.02) | 361.35 (199.33, 597.66) | 0.07 (0.02, 0.12) |
| Nicaragua | 953.18 (475.60, 1661.76) | 1914.45 (967.74, 3324.58) | 95.02 (47.40, 165.61) | 102.84 (51.95, 178.59) | 0.44 (0.37, 0.52) |
| Niger | 895.11 (426.30, 1558.52) | 2766.30 (1344.13, 4885.61) | 47.11 (22.36, 82.08) | 46.71 (22.47, 82.2) | 0.06 (-0.03, 0.15) |
| Nigeria | 15163.53 (8144.59, 25445.18) | 47911.15 (25638.58, 79797.76) | 67.38 (36.35, 112.45) | 77.16 (41.40, 127.91) | 0.86 (0.67, 1.05) |
| Niue | 0.29 (0.14, 0.50) | 0.25 (0.12, 0.43) | 57.83 (28.63, 100.32) | 67.11 (33.50, 116.64) | 0.64 (0.56, 0.72) |
| North Macedonia | 388.06 (198.70, 656.81) | 426.95 (223.41, 714.58) | 76.16 (38.99, 128.95) | 87.50 (45.88, 146.71) | 0.64 (0.53, 0.76) |
| Northern Mariana Islands | 14.70 (7.53, 25.05) | 9.05 (4.58, 15.26) | 97.72 (49.88, 166.62) | 86.43 (44.07, 145.25) | -0.63 (-0.76, -0.51) |
| Norway | 2693.31 (1515.25, 4374.65) | 3459.04 (1947.84, 5589.25) | 260.34 (146.24, 423.95) | 301.24 (169.10, 488.16) | 0.55 (0.50, 0.61) |
| Oman | 504.78 (258.87, 871.35) | 1659.16 (875.07, 2838.21) | 138.92 (71.15, 239.11) | 163.40 (85.73, 280.38) | 0.81 (0.68, 0.93) |
| Pakistan | 15979.19 (8391.23, 27126.83) | 49113.48 (25766.08, 82187.39) | 63.35 (33.25, 107.22) | 77.14 (40.49, 128.98) | 0.71 (0.65, 0.76) |
| Palau | 2.80 (1.43, 4.92) | 2.34 (1.18, 4.01) | 65.12 (33.12, 114.51) | 70.98 (36.05, 121.74) | 0.26 (0.23, 0.29) |
| Palestine | 352.08 (171.31, 617.73) | 1189.26 (602.70, 2038.61) | 72.06 (34.95, 126.06) | 85.68 (43.36, 146.56) | 0.53 (0.48, 0.59) |
| Panama | 813.18 (413.73, 1386.25) | 1819.09 (962.89, 3074.99) | 123.45 (62.70, 210.43) | 169.57 (89.89, 286.55) | 1.13 (1.06, 1.20) |
| Papua New Guinea | 441.23 (213.47, 780.88) | 1281.08 (639.39, 2228.37) | 42.15 (20.28, 74.74) | 47.05 (23.42, 81.91) | 0.24 (0.18, 0.31) |
| Paraguay | 1074.06 (563.26, 1777.50) | 2516.64 (1343.07, 4082.18) | 105.57 (55.26, 174.57) | 128.54 (68.56, 208.67) | 0.65 (0.58, 0.73) |
| Peru | 9199.03 (4623.55, 15969.97) | 19947.16 (10337.83, 34039.85) | 156.48 (78.53, 270.99) | 205.69 (106.56, 351.57) | 0.89 (0.79, 0.99) |
| Philippines | 8196.99 (4474.98, 13625.16) | 18070.27 (9793.89, 29941.62) | 48.85 (26.71, 81.06) | 59.78 (32.43, 98.96) | 0.59 (0.51, 0.66) |
| Poland | 7100.83 (3904.71, 11682.31) | 9752.87 (5638.86, 15517.29) | 77.67 (42.58, 127.90) | 124.77 (71.75, 199.69) | 1.81 (1.73, 1.88) |
| Portugal | 5000.07 (2804.72, 8147.73) | 4893.61 (2709.62, 7964.60) | 199.99 (112.23, 325.89) | 239.96 (133.43, 391.15) | 0.60 (0.46, 0.73) |
| Puerto Rico | 1641.67 (853.94, 2818.49) | 1404.49 (729.67, 2365.37) | 171.70 (89.35, 294.67) | 199.46 (103.77, 336.17) | 0.53 (0.42, 0.63) |
| Qatar | 160.15 (83.77, 267.86) | 1280.14 (687.50, 2147.87) | 194.52 (101.38, 326.66) | 236.92 (125.91, 399.77) | 1.08 (0.94, 1.23) |
| Republic of Korea | 19161.09 (10531.32, 31535.03) | 21427.77 (11894.08, 34610.61) | 143.44 (78.65, 236.20) | 208.54 (116.08, 337.11) | 1.21 (1.15, 1.27) |
| Republic of Moldova | 830.94 (434.31, 1388.94) | 637.25 (347.85, 1047.31) | 73.78 (38.42, 123.15) | 81.41 (44.74, 133.78) | 0.59 (0.30, 0.89) |
| Romania | 4149.29 (2171.12, 7008.24) | 3476.53 (1840.87, 5835.02) | 74.40 (38.96, 125.67) | 97.88 (51.97, 164.03) | 1.20 (1.02, 1.37) |
| Russian Federation | 37191.93 (21166.02, 60367.37) | 33342.55 (19150.82, 53439.89) | 101.20 (57.37, 164.49) | 112.39 (64.15, 181.15) | 0.71 (0.44, 0.98) |
| Rwanda | 861.28 (433.07, 1494.43) | 2181.71 (1075.12, 3798.28) | 48.20 (23.95, 83.94) | 58.42 (28.71, 101.72) | 0.88 (0.68, 1.08) |
| Saint Kitts and Nevis | 15.76 (8.15, 27) | 27.00 (13.86, 45.05) | 142.53 (73.22, 244.36) | 180.10 (92.60, 300.66) | 0.82 (0.74, 0.90) |
| Saint Lucia | 46.36 (23.59, 80.13) | 61.17 (30.89, 104.66) | 123.35 (62.80, 213.10) | 141.26 (71.39, 242.02) | 0.48 (0.45, 0.50) |
| Saint Vincent and the Grenadines | 32.73 (16.87, 56.26) | 36.90 (18.79, 62.81) | 111.63 (57.38, 191.89) | 137.14 (69.95, 233.37) | 0.75 (0.67, 0.82) |
| Samoa | 19.13 (9.42, 32.71) | 27.23 (13.35, 47.83) | 47.36 (22.97, 81.24) | 53.36 (26.03, 93.75) | 0.47 (0.42, 0.53) |
| San Marino | 17.84 (10.02, 29.45) | 18.37 (10.22, 30.05) | 284.16 (159.61, 468.93) | 291.44 (162.71, 477.90) | 0.17 (-0.04, 0.38) |
| Sao Tome and Principe | 16.61 (8.31, 28.96) | 39.80 (20.29, 69.65) | 58.91 (29.19, 102.97) | 67.78 (34.45, 118.30) | 0.64 (0.54, 0.74) |
| Saudi Arabia | 4652.05 (2381.26, 7976.77) | 14687.71 (7690.40, 24674.98) | 131.01 (67.26, 224.34) | 147.61 (77.32, 248.47) | 0.56 (0.46, 0.66) |
| Senegal | 1142.89 (586.60, 1964.17) | 2769.38 (1409.20, 4699.96) | 60.76 (30.95, 104.31) | 66.29 (33.68, 112.54) | 0.30 (0.24, 0.36) |
| Serbia | 1869.32 (979.98, 3132.35) | 1734.61 (923.44, 2900.01) | 81.88 (42.96, 137.30) | 92.69 (49.55, 155.39) | 0.76 (0.56, 0.95) |
| Seychelles | 13.84 (6.93, 23.41) | 19.95 (10.24, 34.15) | 69.72 (34.68, 118.05) | 86.96 (44.72, 148.93) | 0.66 (0.60, 0.72) |
| Sierra Leone | 590.80 (292.64, 1028.85) | 1301.49 (648.22, 2271.90) | 53.38 (26.23, 93.04) | 52.29 (25.96, 91.26) | -0.02 (-0.18, 0.13) |
| Singapore | 1800.93 (984.06, 2936.33) | 3366.01 (1910.60, 5467.82) | 187.21 (102.21, 305.44) | 265.72 (151.55, 432.83) | 1.17 (1.13, 1.22) |
| Slovakia | 1153.39 (592.75, 1940.23) | 1227.23 (652.19, 2058.09) | 88.56 (45.44, 149.11) | 109.74 (58.46, 184.37) | 0.94 (0.85, 1.04) |
| Slovenia | 476.25 (248.44, 811.72) | 414.70 (219.67, 700.00) | 97.31 (50.82, 166.05) | 112.52 (59.84, 190.56) | 0.66 (0.60, 0.72) |
| Solomon Islands | 31.77 (15.46, 55.63) | 71.50 (35.62, 124.10) | 38.50 (18.51, 67.45) | 40.13 (19.90, 69.75) | 0.02 (-0.07, 0.11) |
| Somalia | 567.75 (270.79, 1016.47) | 1453.30 (657.06, 2647.21) | 31.59 (14.94, 56.68) | 27.56 (12.35, 50.30) | -0.38 (-0.47, -0.28) |
| South Africa | 9626.05 (5191.06, 15960.86) | 15522.94 (8435.53, 25618.30) | 92.38 (49.99, 152.84) | 100.13 (54.23, 165.43) | 0.43 (0.37, 0.49) |
| South Sudan | 1082.59 (562.98, 1864.05) | 1372.15 (684.98, 2389.81) | 75.61 (39.10, 129.65) | 56.51 (28.19, 98.32) | -0.65 (-0.86, -0.44) |
| Spain | 29293.63 (16502.54, 46466.80) | 32314.92 (17979.67, 51969.20) | 302.90 (170.71, 480.33) | 382.00 (213.14, 615.97) | 0.81 (0.72, 0.90) |
| Sri Lanka | 2182.02 (1056.17, 3730.52) | 3727.08 (1905.27, 6362.29) | 45.46 (21.96, 77.76) | 68.41 (35.07, 116.78) | 1.39 (1.33, 1.45) |
| Sudan | 3399.67 (1639.91, 5863.56) | 9851.84 (4809.86, 16972.16) | 66.81 (32.19, 114.96) | 82.30 (40.23, 141.52) | 0.97 (0.88, 1.06) |
| Suriname | 137.43 (71.58, 229.68) | 209.32 (109.25, 347.87) | 131.74 (68.53, 220.32) | 147.20 (76.92, 244.56) | 0.64 (0.55, 0.72) |
| Sweden | 5259.77 (2871.60, 8633.84) | 7076.48 (3886.45, 11668.95) | 270.49 (147.45, 445.82) | 340.60 (186.20, 565.39) | 0.82 (0.72, 0.91) |
| Switzerland | 4481.69 (2700.10, 6954.12) | 5147.57 (3063.08, 8003.09) | 262.83 (158.22, 408.00) | 285.70 (170.37, 445.78) | 0.28 (0.24, 0.31) |
| Syrian Arab Republic | 2421.00 (1251.30, 4112.56) | 3226.02 (1592.71, 5629.73) | 79.67 (41.05, 134.63) | 85.01 (42.29, 146.83) | 0.38 (0.27, 0.49) |
| Taiwan (Province of China) | 3885.83 (2013.63, 6549.40) | 4665.51 (2395.00, 7802.14) | 67.44 (34.78, 113.81) | 93.40 (48.13, 156.09) | 0.99 (0.89, 1.09) |
| Tajikistan | 888.58 (458.59, 1495.21) | 1527.35 (779.24, 2592.94) | 64.99 (33.34, 109.66) | 57.53 (29.26, 97.78) | -0.04 (-0.46, 0.38) |
| Thailand | 9000.29 (4552.68, 15640.53) | 11057.34 (5610.25, 19078.79) | 53.19 (26.75, 92.62) | 75.27 (38.47, 130.00) | 1.06 (1.02, 1.11) |
| Timor-Leste | 86.04 (42.60, 150.89) | 211.52 (106.18, 363.47) | 42.78 (21.03, 75) | 56.10 (28.07, 96.66) | 1.21 (1.03, 1.40) |
| Togo | 492.18 (242.51, 854.14) | 1227.21 (605.77, 2077.28) | 52.71 (25.93, 91.07) | 54.58 (26.88, 92.49) | 0.07 (-0.02, 0.15) |
| Tokelau | 0.18 (0.09, 0.32) | 0.18 (0.09, 0.32) | 47.77 (23.15, 85.68) | 57.70 (29.06, 100.43) | 0.71 (0.66, 0.76) |
| Tonga | 11.41 (5.58, 20.13) | 13.95 (6.85, 24.15) | 47.44 (23.15, 83.59) | 53.67 (26.28, 93.09) | 0.33 (0.30, 0.36) |
| Trinidad and Tobago | 475.61 (246.56, 817.19) | 616.85 (321.32, 1042.84) | 146.52 (75.76, 251.97) | 194.14 (101.19, 328.15) | 1.42 (1.26, 1.58) |
| Tunisia | 1989.51 (1019.45, 3458.54) | 3271.75 (1650.94, 5604.03) | 90.08 (46.07, 156.34) | 112.66 (56.87, 193.42) | 0.79 (0.73, 0.85) |
| Turkey | 18349.78 (9369.91, 31192.78) | 35546.69 (18215.47, 60869.10) | 122.56 (62.86, 207.59) | 170.02 (87.29, 291.67) | 1.46 (1.36, 1.57) |
| Turkmenistan | 734.31 (380.48, 1230.80) | 1264.38 (663.09, 2140.83) | 74.52 (38.45, 125.04) | 99.66 (52.20, 168.72) | 1.36 (1.03, 1.69) |
| Tuvalu | 0.98 (0.48, 1.72) | 1.44 (0.71, 2.52) | 39.40 (19.14, 69.23) | 47.53 (23.53, 83.36) | 0.45 (0.39, 0.52) |
| Uganda | 1983.13 (947.67, 3538.36) | 6702.51 (3414.39, 11632.26) | 45.87 (21.86, 81.66) | 58.43 (29.59, 101.09) | 0.92 (0.88, 0.96) |
| Ukraine | 10452.60 (5471.60, 17639.54) | 7093.85 (3737.37, 12031.36) | 84.98 (44.54, 143.35) | 81.05 (43.34, 138.03) | 0.20 (-0.07, 0.47) |
| United Arab Emirates | 746.21 (390.61, 1265.14) | 2484.96 (1326.85, 4173.66) | 199.23 (103.92, 338.47) | 171.44 (91.50, 290.15) | -0.49 (-0.55, -0.43) |
| United Kingdom | 31786.90 (18006.72, 51010.16) | 40359.89 (23172.82, 64490.90) | 227.86 (129.27, 366.19) | 277.80 (159.09, 445.27) | 0.70 (0.61, 0.79) |
| United Republic of Tanzania | 3479.04 (1720.55, 6062.51) | 10268.33 (5118.56, 18001.66) | 52.34 (25.73, 90.98) | 64.34 (32.03, 112.54) | 0.89 (0.77, 1.00) |
| United States of America | 184147.48 (106000.95, 296438.61) | 194771.16 (112350.74, 315058.33) | 278.99 (160.46, 450.47) | 268.87 (154.68, 436.19) | -0.31 (-0.47, -0.15) |
| United States Virgin Islands | 47.87 (25.17, 82.07) | 34.33 (18.45, 57.77) | 175.92 (92.70, 301.02) | 219.13 (117.87, 368.51) | 0.72 (0.48, 0.96) |
| Uruguay | 1259.59 (678.11, 2117.58) | 1660.04 (902.17, 2775.99) | 168.22 (90.57, 282.78) | 207.60 (113.02, 347.48) | 0.63 (0.52, 0.74) |
| Uzbekistan | 3442.67 (1746.82, 5929.83) | 6816.11 (3530.83, 11678.03) | 62.86 (31.63, 108.48) | 77.24 (39.95, 132.35) | 0.89 (0.68, 1.10) |
| Vanuatu | 16.09 (8.16, 27.81) | 35.83 (17.60, 63.88) | 42.09 (21.08, 72.85) | 43.57 (21.31, 77.69) | 0.12 (0.10, 0.14) |
| Venezuela | 7405.94 (3879.98, 12517.25) | 8500.99 (4312.63, 14547.83) | 141.76 (74.08, 239.60) | 132.80 (67.56, 227.09) | 0.09 (-0.04, 0.22) |
| Viet Nam | 6809.61 (3229.52, 11917.74) | 14163.61 (6997.20, 24375.78) | 35.99 (16.92, 63.14) | 58.02 (28.62, 99.70) | 1.63 (1.60, 1.65) |
| Yemen | 2103.03 (1064.68, 3608.95) | 6133.52 (3041.29, 10550.49) | 70.74 (35.70, 121.37) | 69.34 (34.37, 119.35) | 0.25 (0.06, 0.43) |
| Zambia | 1209.84 (619.58, 2083.37) | 3752.78 (1921.65, 6465.92) | 59.08 (30.06, 101.29) | 69.92 (35.61, 120.13) | 0.90 (0.70, 1.09) |
| Zimbabwe | 1823.85 (922.66, 3143.31) | 2608.87 (1314.47, 4472.36) | 68.53 (34.50, 117.94) | 60.46 (30.38, 103.59) | -0.66 (-0.83, -0.49) |

Abbreviations: DALYs, disability-adjusted life-years; ED, eating disorder; WCBA, women of childbearing age; ASDR, age-standardized DALY rate; EAPC, estimated annual percentage change; UI, uncertainty interval; CI, confidence interval
