# Supplementary material for: Patterns and trends of eating disorders among women of childbearing age: a comprehensive analysis from 1990 to 2021 with future predictions
Source: Eat Weight Disord. 2026 Mar 23;31(1):40. doi: 10.1007/s40519-026-01842-8 (PMC13132930; doi:10.1007/s40519-026-01842-8)
Supplement: Supplementary file 14 — Supplementary Material 14. [file 40519_2026_1842_MOESM14_ESM.docx]

Table S4. DALYs of AN cases among WCBA in 1990 and 2021 at the national level, along with their EAPCs from 1990 to 2021.

| Location | DALYs | | | | |
| --- | --- | --- | --- | --- | --- |
|  | Number of cases(95% UI) | | ASDR per 100,000 population (95% UI) | | EAPC(95% CI) |
|  | 1990 | 2021 | 1990 | 2021 | 1990-2021 |
| Afghanistan | 355.02 (144.16, 664.24) | 1115.39 (456.31, 2152.49) | 13.63 (5.43, 25.72) | 13.20 (5.23, 25.73) | 0.14 (-0.07, 0.35) |
| Albania | 177.30 (74.40, 333.39) | 142.51 (61.69, 265.14) | 18.30 (7.33, 34.29) | 23.25 (9.95, 43.33) | 0.98 (0.89, 1.08) |
| Algeria | 1471.29 (644.21, 2725.54) | 2241.79 (960.33, 4247.27) | 20.91 (8.92, 38.90) | 21.30 (9.26, 40.01) | 0.19 (0.14, 0.24) |
| American Samoa | 2.80 (1.26, 5.21) | 2.11 (0.84, 3.89) | 20.15 (9.04, 38.34) | 18.17 (7.13, 33.52) | -0.30 (-0.33, -0.27) |
| Andorra | 13.96 (7.52, 23.25) | 15.53 (8.26, 26.39) | 82.36 (44.34, 140.85) | 85.72 (46.46, 146.60) | 0.24 (0.15, 0.33) |
| Angola | 435.66 (181.06, 802.42) | 1669.83 (748.27, 3107.99) | 16.41 (6.91, 30.44) | 18.86 (8.41, 35.62) | 0.78 (0.66, 0.89) |
| Antigua and Barbuda | 4.51 (2.02, 8.09) | 6.28 (2.86, 11.66) | 25.03 (11.28, 44.73) | 27.60 (12.73, 51.02) | 0.31 (0.25, 0.37) |
| Argentina | 3772.24 (1830.99, 6649.52) | 5547.98 (2752.37, 9770.89) | 43.18 (21.06, 76.53) | 45.18 (22.41, 80.19) | 0.05 (-0.01, 0.12) |
| Armenia | 188.20 (81.55, 348.99) | 158.40 (67.82, 294.86) | 20.18 (8.69, 38.05) | 23.93 (10.39, 44.50) | 1.00 (0.77, 1.22) |
| Australia | 4488.30 (2412.30, 7528.03) | 6994.42 (3872.53, 11686.28) | 93.00 (48.81, 158.84) | 112.72 (60.89, 189.96) | 1.28 (0.95, 1.60) |
| Austria | 1412.02 (788.25, 2362.56) | 1678.13 (944.10, 2679.19) | 65.22 (36.45, 111.15) | 83.45 (46.64, 134.45) | 0.93 (0.87, 1.00) |
| Azerbaijan | 504.50 (225.56, 925.62) | 719.54 (332.11, 1344.85) | 23.04 (10.23, 42.95) | 27.19 (12.54, 50.80) | 1.04 (0.65, 1.42) |
| Bahamas | 25.12 (11.40, 45.27) | 31.49 (14.44, 57.23) | 30.42 (13.30, 55.62) | 29.43 (13.57, 53.49) | -0.01 (-0.09, 0.07) |
| Bahrain | 34.97 (15.84, 62.54) | 90.54 (40.04, 165.87) | 26.49 (11.78, 47.48) | 28.22 (12.69, 50.75) | 0.23 (0.19, 0.26) |
| Bangladesh | 3985.62 (1604.47, 7679.72) | 8076.04 (3280.34, 15107.74) | 13.64 (5.34, 26.62) | 17.12 (7.03, 31.67) | 0.76 (0.67, 0.86) |
| Barbados | 18.47 (8.28, 34.28) | 15.94 (6.99, 29.70) | 25.43 (11.15, 46.94) | 24.46 (10.62, 45.58) | -0.14 (-0.24, -0.04) |
| Belarus | 684.25 (310.29, 1263.36) | 564.33 (252.57, 1028.25) | 26.49 (12.30, 48.98) | 31.31 (14.90, 56.72) | 0.83 (0.65, 1.01) |
| Belgium | 1917.92 (1043.19, 3199.31) | 2263.25 (1298.38, 3756.87) | 71.85 (38.74, 119.87) | 87.53 (49.74, 147.38) | 0.76 (0.70, 0.83) |
| Belize | 9.80 (4.15, 18.09) | 27.23 (12.10, 50.76) | 19.05 (7.79, 35.73) | 20.85 (9.20, 39.28) | 0.29 (0.22, 0.36) |
| Benin | 175.44 (71.39, 340.55) | 561.59 (233.28, 1055.84) | 14.01 (5.62, 27.54) | 14.94 (6.09, 28.18) | 0.27(0.23,0.31) |
| Bermuda | 5.37 (2.42, 9.86) | 4.11 (1.86, 7.52) | 32.21 (14.19, 58.83) | 35.48 (16.57, 65) | 0.45(0.39,0.52) |
| Bhutan | 26.36 (10.20, 50.08) | 44.92 (19.40, 82.60) | 15.21 (5.73, 29.13) | 20.99 (8.86, 38.67) | 1.09(1.06,1.12) |
| Bolivia | 278.65 (120.18, 527.77) | 618.98 (267.47, 1149.53) | 16.22 (6.95, 30.9) | 19.21 (8.57, 36.05) | 0.58(0.53,0.62) |
| Bosnia and Herzegovina | 184.80 (76.96, 358.31) | 164.58 (73.69, 301.25) | 15.39 (6.31, 30.06) | 24.63 (11.18, 45.61) | 1.85(1.65,2.04) |
| Botswana | 77.45 (35.05, 144.1) | 176.54 (78.91, 326.86) | 19.92 (8.87, 37.73) | 25.1 (10.82, 46.27) | 0.77(0.74,0.81) |
| Brazil | 12589.45 (6906.30, 21146.18) | 18226.55 (10127.31, 30557.95) | 28.69 (15.56, 48.22) | 32.62 (18.01, 54.8) | 0.53(0.48,0.58) |
| Brunei Darussalam | 79.80 (41.99, 135.61) | 130.85 (71.39, 216.47) | 93.41 (48.77, 162.59) | 95.43 (52.33, 159.02) | 0.29(0.21,0.37) |
| Bulgaria | 469.69 (201.91, 869.3) | 330.93 (148.10, 598.08) | 23.37 (10.11, 44.12) | 27.19 (12.26, 49.12) | 0.69(0.56,0.83) |
| Burkina Faso | 296.12 (119.55, 560.93) | 871.62 (350.25, 1691.54) | 12.59 (5.05, 24.16) | 13.77 (5.36, 27.05) | 0.31(0.27,0.35) |
| Burundi | 181.64 (71.95, 361.03) | 399.27 (149.15, 760.48) | 12.66 (4.95, 25.09) | 11.18 (4.14, 21.25) | -0.36(-0.42,-0.30) |
| Cabo Verde | 14.36 (5.94, 27.02) | 30.36 (13.13, 57.08) | 15.09 (5.92, 28.74) | 19.52 (8.33, 37.47) | 0.95(0.88,1.02) |
| Cambodia | 299.01 (114.25, 586.52) | 637.71 (249.44, 1204.83) | 10.72 (4.12, 21.2) | 14.06 (5.72, 26.74) | 0.93(0.85,1.00) |
| Cameroon | 460.00 (202.24, 859.04) | 1442.93 (643.23, 2709.65) | 16.49 (7.1, 31.37) | 16.16 (7.05, 30.38) | 0.05(-0.01,0.11) |
| Canada | 4881.46 (2493.04, 8539.3) | 5694.22 (3017.62, 9805.20) | 64.52 (31.8, 114.78) | 69.95 (36.39, 120.42) | 0.34(0.30,0.39) |
| Central African Republic | 92.67 (36.22, 177.13) | 173.14 (62.54, 342.28) | 12.77 (4.89, 24.41) | 11.18 (3.99, 22.31) | -0.41(-0.44,-0.38) |
| Chad | 193.37 (75.73, 376.8) | 652.04 (261.24, 1234.57) | 12.75 (5.14, 25.15) | 14.15 (5.63, 26.96) | 0.47(0.40,0.54) |
| Chile | 1590.33 (777.68, 2801.54) | 2280.37 (1111.11, 3982.40) | 38.46 (18.85, 67.79) | 47.31 (23.23, 82.82) | 0.59(0.51,0.67) |
| China | 41859.47 (22505.54, 72816) | 46332.79 (25507.23, 79886.75) | 11.81 (6.26, 20.75) | 18.22 (9.93, 31.45) | 1.63(1.56,1.69) |
| Colombia | 2084.75 (913.85, 3846.34) | 3219.15 (1407.64, 5852.63) | 21.09 (9.08, 39.02) | 24.47 (10.52, 44.46) | 0.44(0.38,0.50) |
| Comoros | 19.88 (8.43, 37.27) | 31.70 (12.86, 60.08) | 15.99 (6.64, 30.06) | 15.37 (6.19, 29.38) | -0.14(-0.19,-0.09) |
| Congo | 117.90 (54.11, 219.37) | 279.55 (113.75, 519.99) | 17.45 (7.9, 32.5) | 18.26 (7.46, 34.21) | 0.32(0.25,0.40) |
| Cook Islands | 0.95 (0.40, 1.78) | 0.93 (0.42, 1.77) | 18.62 (7.58, 35.01) | 22.05 (10.03, 41.56) | 0.47(0.41,0.52) |
| Costa Rica | 186.80 (81.97, 345.59) | 330.99 (154.28, 606.45) | 21.45 (8.98, 40.08) | 25.98 (12.1, 47.33) | 0.64(0.57,0.70) |
| Cote d'Ivoire | 538.90 (225.76, 1015.51) | 1241.79 (525.98, 2352.49) | 16.57 (6.84, 31.31) | 16.78 (7.09, 31.88) | -0.01(-0.11,0.10) |
| Croatia | 302.53 (136.12, 557.03) | 299.16 (155.02, 513.76) | 25.28 (11.2, 46.78) | 35.01 (18.37, 60.42) | 1.50(1.35,1.66) |
| Cuba | 706.9 (302.62, 1308.49) | 472.34 (187.52, 915.76) | 21.05 (8.8, 39.16) | 21.38 (8.82, 40.77) | 0.27(0.13,0.41) |
| Cyprus | 131.61 (69.93, 223.84) | 227.93 (121.05, 384.79) | 60.33 (31.91, 103.69) | 65.79 (34.34, 113.17) | 0.32(0.21,0.43) |
| Czechia | 702.18 (324.32, 1303.49) | 762.78 (397.26, 1296.82) | 27.96 (12.8, 51.61) | 38.11 (19.72, 65.56) | 1.28(1.18,1.38) |
| Democratic People's Republic of Korea | 772.95 (292.83, 1497.55) | 723.42 (264, 1401.15) | 13 (4.91, 25.26) | 12.2 (4.47, 23.49) | -0.06(-0.14,0.02) |
| Democratic Republic of the Congo | 1378.85 (566.14, 2560.88) | 2806.58 (1085.46, 5401.86) | 14 (5.68, 25.81) | 11.6 (4.5, 22.51) | -0.54(-0.77,-0.30) |
| Denmark | 1057.88 (592.76, 1716.03) | 1073.8 (600.86, 1785.78) | 75.4 (41.02, 123.31) | 80.21 (44.75, 134.27) | 0.16(0.10,0.23) |
| Djibouti | 20.53 (8.84, 38.05) | 54.09 (21.7, 101.85) | 17.44 (7.48, 32.34) | 16.97 (6.92, 32.2) | -0.05(-0.16,0.07) |
| Dominica | 4.11 (1.79, 7.66) | 3.74 (1.64, 7.02) | 20.95 (8.76, 39.32) | 22.54 (9.86, 42.21) | 0.27(0.24,0.30) |
| Dominican Republic | 440.8 (193.52, 800.29) | 753.42 (329.37, 1386.25) | 19.84 (8.63, 36.65) | 24.94 (11, 46.15) | 0.76(0.72,0.80) |
| Ecuador | 581.16 (244.11, 1069.97) | 1054.23 (459.73, 1980.6) | 19.97 (8.16, 36.83) | 21.8 (9.43, 40.98) | 0.42(0.35,0.49) |
| Egypt | 2498.26 (1060.22, 4679.43) | 5626.19 (2479.62, 10434.25) | 17.28 (7.22, 32.43) | 20.61 (9.04, 38) | 0.59(0.56,0.62) |
| El Salvador | 272.37 (116.01, 504.19) | 393.46 (174.72, 729.42) | 17.78 (7.29, 32.88) | 21.46 (9.5, 39.89) | 0.59(0.55,0.64) |
| Equatorial Guinea | 15.74 (6.36, 29.75) | 112.33 (50.86, 202.92) | 14.09 (5.69, 26.69) | 26.7 (11.6, 48.87) | 2.92(2.40,3.45) |
| Eritrea | 113.21 (45.39, 217.97) | 251.54 (103.03, 478.2) | 12.64 (4.96, 24.35) | 13.97 (5.76, 26.67) | 0.15(0.01,0.30) |
| Estonia | 102.16 (46.62, 186.69) | 87.08 (42.39, 158.73) | 28.19 (12.86, 51.36) | 36.72 (18.29, 65.87) | 1.00(0.93,1.07) |
| Eswatini | 45.01 (19.25, 83.86) | 75.81 (32.31, 142.38) | 18.98 (7.88, 36.07) | 21.56 (9.12, 41.2) | 0.31(0.28,0.35) |
| Ethiopia | 1433.04 (725.69, 2532.99) | 4141.55 (2111.55, 7275.53) | 11.12 (5.55, 19.68) | 13.02 (6.55, 22.96) | 0.62(0.46,0.78) |
| Fiji | 31.4 (12.76, 60.02) | 37.06 (14.64, 70.33) | 15 (6.18, 28.84) | 16.6 (6.75, 31.08) | 0.28(0.24,0.33) |
| Finland | 1217.4 (679.51, 1967.54) | 1149.83 (640.91, 1884.13) | 96.02 (53.71, 152.72) | 98.82 (55.17, 162.89) | 0.16(0.04,0.27) |
| France | 12127.66 (6723.44, 20040.73) | 12272.58 (6769.04, 20428.42) | 75.25 (41.82, 123.82) | 83.1 (45.18, 138.88) | 0.34(0.32,0.36) |
| Gabon | 65.37 (29.58, 119.2) | 131.56 (61.36, 241.8) | 24.16 (10.32, 44.77) | 23.8 (10.86, 44.98) | -0.00(-0.03,0.02) |
| Gambia | 41.64 (16.71, 79.33) | 102.83 (41.49, 193.48) | 15.38 (5.96, 29.22) | 14.48 (5.66, 27.47) | -0.18(-0.21,-0.15) |
| Georgia | 345.47 (149.48, 632.65) | 173.68 (75.85, 317.56) | 24.13 (10.39, 44.03) | 24.75 (11.22, 44.69) | 0.52(0.18,0.87) |
| Germany | 19598.57 (11304.82, 32050.11) | 18242.44 (10454.88, 29563.43) | 89.37 (50.38, 146.52) | 100.87 (57.38, 165.44) | 0.36(0.31,0.41) |
| Ghana | 614.93 (239.17, 1177.94) | 1802.08 (782.19, 3408.85) | 15.18 (5.83, 29.14) | 18.16 (7.7, 34.32) | 0.58(0.53,0.63) |
| Greece | 1941.17 (1024.82, 3199.22) | 1749.51 (954.21, 2907.09) | 70.45 (36.81, 116.56) | 84.9 (45.64, 141.63) | 0.77(0.65,0.90) |
| Greenland | 13.8 (7.19, 23.64) | 12.19 (6.45, 20.53) | 77.99 (40.18, 135.91) | 85.76 (44.39, 147.38) | 0.46(0.38,0.53) |
| Grenada | 4.54 (1.96, 8.59) | 6.34 (2.96, 11.55) | 20.2 (8.59, 38.69) | 24.23 (11.4, 45.05) | 0.63(0.58,0.69) |
| Guam | 9.56 (4.16, 17.86) | 8.82 (3.76, 16.72) | 25.26 (10.82, 47.25) | 24.94 (10.5, 46.91) | 0.06(0.02,0.09) |
| Guatemala | 399.26 (170.33, 738.79) | 1030.53 (454.46, 1914.58) | 18.65 (7.9, 34.87) | 21.23 (9.21, 40.14) | 0.46(0.42,0.49) |
| Guinea | 213.98 (89.72, 403.28) | 538.16 (225.89, 1007.05) | 14.37 (6.1, 27.3) | 14.27 (5.86, 26.54) | -0.08(-0.16,0.00) |
| Guinea-Bissau | 36.29 (14.64, 69.53) | 79.39 (30.74, 149.51) | 13.53 (5.31, 25.63) | 13.49 (5.28, 25.45) | -0.07(-0.12,-0.01) |
| Guyana | 40.95 (16.78, 79.41) | 46.66 (20.88, 84.81) | 17.28 (6.91, 33.46) | 21.7 (9.58, 40.09) | 0.65(0.59,0.70) |
| Haiti | 264.03 (108.49, 494.5) | 516.68 (209.18, 972.24) | 15.37 (6.15, 28.81) | 14.35 (6, 27.09) | -0.16(-0.21,-0.11) |
| Honduras | 215.95 (89.35, 403.73) | 598.94 (256.56, 1128.3) | 17.45 (6.98, 33.17) | 19.31 (8.14, 36.98) | 0.30(0.27,0.34) |
| Hungary | 575.9 (248.53, 1057.03) | 651.13 (328.3, 1138.64) | 23.24 (9.87, 42.99) | 34.05 (17.25, 59.07) | 1.59(1.45,1.73) |
| Iceland | 53.18 (28.63, 88.81) | 64.2 (33.77, 108.28) | 70.83 (37.9, 119.76) | 75.33 (39.74, 128.05) | 0.28(0.23,0.33) |
| India | 28805.63 (15781.67, 49275.25) | 69158.96 (38114.89, 115422.34) | 13.22 (7.12, 22.8) | 17.82 (9.74, 29.96) | 1.03(0.99,1.06) |
| Indonesia | 7217.65 (3972.52, 12585.48) | 12221.02 (6660.31, 20679.48) | 13.58 (7.38, 24.12) | 17.13 (9.21, 28.89) | 0.65(0.58,0.72) |
| Iran (Islamic Republic of) | 3375.16 (1836.41, 5737.85) | 5752.64 (3099.59, 9843.55) | 22.51 (12.19, 38.29) | 29.64 (16.08, 50.45) | 1.21(1.03,1.40) |
| Iraq | 976.45 (434.76, 1813.1) | 2508.92 (1109.04, 4602.91) | 20.21 (8.84, 38.26) | 22.04 (9.52, 41.02) | 0.61(0.46,0.77) |
| Ireland | 552.89 (294.73, 924.9) | 868.43 (472.62, 1446.03) | 55.49 (29.4, 93.97) | 75.15 (40.95, 126.88) | 0.95(0.82,1.07) |
| Israel | 544.19 (281.32, 909.42) | 1165.65 (599.04, 1960.89) | 37.88 (19.3, 64.15) | 47.1 (24.33, 80.15) | 0.84(0.71,0.97) |
| Italy | 11187.81 (6528.26, 17609.64) | 7067.84 (4020.07, 11220.25) | 70.7 (41.3, 111.87) | 62.2 (35.54, 99.42) | -0.44(-0.52,-0.35) |
| Jamaica | 141.02 (63.21, 258.26) | 165.78 (70.39, 314.49) | 20.22 (8.98, 37.38) | 21.18 (9.37, 40.18) | 0.11(0.06,0.15) |
| Japan | 25846.31 (14950, 41631.64) | 25625.74 (15752.01, 39732.78) | 79.5 (45.56, 128.12) | 105.15 (64.6, 165.15) | 0.83(0.68,0.98) |
| Jordan | 194.31 (85.05, 358.88) | 649.96 (283.62, 1208.62) | 18.79 (7.82, 35.15) | 19.54 (8.48, 36.29) | 0.33(0.24,0.42) |
| Kazakhstan | 1107.81 (476.81, 2050.79) | 1406.62 (646.65, 2540.64) | 24.72 (10.26, 45.72) | 30.61 (13.98, 55.21) | 1.03(0.80,1.26) |
| Kenya | 937.26 (508.01, 1600.34) | 2352.87 (1301.17, 3958.13) | 14.86 (7.93, 25.4) | 15.64 (8.58, 26.44) | 0.14(0.07,0.21) |
| Kiribati | 2.38 (0.93, 4.62) | 3.75 (1.43, 7.19) | 11.72 (4.69, 22.48) | 11.61 (4.6, 21.99) | -0.10(-0.14,-0.05) |
| Kuwait | 162.32 (76.52, 299.29) | 463.95 (212.12, 827.86) | 33.6 (15.75, 62.61) | 35.86 (16.51, 63.17) | 0.31(0.19,0.42) |
| Kyrgyzstan | 243.24 (106.28, 446.2) | 340.45 (145.62, 636.67) | 20.06 (8.38, 37.47) | 19.28 (8.47, 36.5) | -0.01(-0.26,0.25) |
| Lao People's Democratic Republic | 125.68 (46.51, 245.02) | 333.98 (134.58, 637.66) | 11.6 (4.26, 22.99) | 16.17 (6.55, 31.23) | 1.15(1.09,1.22) |
| Latvia | 177.19 (82.88, 321.58) | 118.79 (56.5, 216.61) | 28.06 (13.1, 51) | 34.97 (16.68, 63.71) | 0.98(0.83,1.13) |
| Lebanon | 161.27 (70.67, 294.8) | 285.26 (117.69, 537.43) | 19.96 (8.65, 36.23) | 21.14 (8.68, 39.16) | 0.36(0.29,0.43) |
| Lesotho | 57.77 (22.75, 108.98) | 92.32 (40.32, 168.31) | 13.93 (5.36, 26.36) | 16.29 (7.07, 29.71) | 0.60(0.57,0.62) |
| Liberia | 85.27 (34.36, 162.68) | 185.05 (72.98, 353.1) | 13.36 (5.21, 25.38) | 12.16 (4.83, 23.16) | 0.04(-0.12,0.19) |
| Libya | 313.08 (148.6, 573.67) | 359.33 (150.62, 677.67) | 27.19 (12.42, 50.48) | 19.23 (8.17, 35.71) | -0.83(-1.02,-0.64) |
| Lithuania | 249.45 (114.67, 459.17) | 181.16 (87.52, 319.03) | 26.98 (12.02, 49.17) | 34.48 (16.93, 61.05) | 1.14(1.00,1.28) |
| Luxembourg | 88.82 (49.5, 145.45) | 165.98 (91.54, 271.2) | 83.33 (45.84, 137.43) | 103.6 (57.52, 167.4) | 0.75(0.69,0.81) |
| Madagascar | 440.55 (183.47, 866.87) | 1121.08 (455.87, 2094.55) | 13.96 (5.7, 28.12) | 13.68 (5.41, 25.79) | -0.01(-0.06,0.04) |
| Malawi | 323.15 (123.7, 620.75) | 743.85 (295.72, 1403.33) | 12.19 (4.57, 23.29) | 12.67 (4.99, 24.06) | 0.18(0.15,0.22) |
| Malaysia | 883.89 (376.37, 1682.76) | 1977.07 (902.85, 3708.77) | 17.95 (7.62, 34.81) | 22.93 (10.37, 43.25) | 0.78(0.73,0.83) |
| Maldives | 8.88 (3.64, 16.74) | 21.15 (9.07, 39.79) | 15.32 (6, 29.29) | 19.99 (8.57, 37.64) | 0.87(0.81,0.93) |
| Mali | 264.9 (96.49, 518.41) | 933.63 (369.49, 1723.78) | 12.33 (4.46, 24.18) | 14.52 (5.7, 27.14) | 0.51(0.48,0.55) |
| Malta | 50.46 (24.83, 86.88) | 59.6 (30.42, 103.01) | 52.02 (25.88, 90.78) | 65.13 (33.42, 113.47) | 0.63(0.56,0.70) |
| Marshall Islands | 1.44 (0.52, 2.83) | 1.95 (0.76, 3.69) | 12.61 (4.57, 24.71) | 12.97 (5.22, 24.46) | 0.02(-0.01,0.05) |
| Mauritania | 83.76 (34.06, 160.15) | 204.47 (87.89, 374.53) | 15.4 (6.32, 29.34) | 16.66 (7.01, 30.76) | 0.29(0.24,0.34) |
| Mauritius | 53.5 (22.09, 100.15) | 61.08 (26.28, 114.48) | 17.01 (6.97, 31.55) | 20.61 (9.11, 37.83) | 0.56(0.49,0.63) |
| Mexico | 5954.82 (3228.76, 10142.98) | 8442.06 (4618.12, 14178.24) | 23.2 (12.45, 39.55) | 24.51 (13.23, 41.12) | 0.17(0.11,0.22) |
| Micronesia (Federated States of) | 3.34 (1.26, 6.37) | 3.53 (1.38, 6.86) | 12.51 (4.71, 23.95) | 12.86 (4.92, 24.75) | 0.04(-0.01,0.09) |
| Monaco | 8.13 (4.38, 13.61) | 9.22 (5.08, 15.17) | 117.16 (63.17, 194.42) | 131.84 (73.71, 221.31) | 0.39(0.37,0.42) |
| Mongolia | 114.36 (50.25, 212.33) | 203.5 (88.54, 384.8) | 18.46 (7.74, 34.99) | 24.85 (10.8, 47.42) | 1.20(1.05,1.35) |
| Montenegro | 41.01 (17.9, 74.31) | 37.61 (16.75, 69.16) | 24.55 (10.56, 44.58) | 27.34 (11.96, 50.24) | 0.68(0.51,0.84) |
| Morocco | 1180.97 (505.59, 2214.83) | 1751.63 (768.8, 3270.14) | 16.62 (7.11, 31.46) | 18.58 (8.21, 34.22) | 0.34(0.28,0.39) |
| Mozambique | 359.44 (124.72, 710.82) | 1124.86 (465.35, 2165.5) | 10.26 (3.6, 20.31) | 12.61 (4.94, 24.77) | 0.79(0.74,0.84) |
| Myanmar | 1203.49 (421.61, 2323.55) | 2446.89 (973.12, 4613.46) | 10.35 (3.59, 20.08) | 16.17 (6.67, 30.22) | 1.59(1.49,1.70) |
| Namibia | 81.35 (34.88, 150.74) | 163.93 (69.35, 301.79) | 19.92 (8.28, 37.4) | 22.62 (9.3, 41.97) | 0.56(0.48,0.65) |
| Nauru | 0.53 (0.23, 0.99) | 0.53 (0.23, 0.99) | 19.7 (8.48, 37.51) | 17.57 (7.51, 32.57) | -0.37(-0.70,-0.05) |
| Nepal | 657.36 (257.09, 1242.17) | 1500.93 (610.31, 2864.46) | 12.86 (4.88, 24.36) | 15.55 (6.42, 29.6) | 0.64(0.59,0.69) |
| Netherlands | 2329.13 (1332.87, 3701.73) | 3442.57 (2019.18, 5448.19) | 52.17 (29.8, 83.19) | 86.66 (50.29, 137.8) | 2.24(2.04,2.45) |
| New Zealand | 932.76 (492.46, 1580.1) | 1092.77 (587.98, 1858.41) | 92.74 (48.83, 157.73) | 86.27 (45.83, 148.11) | -0.02(-0.12,0.08) |
| Nicaragua | 189.4 (78.45, 354.6) | 362.88 (148.91, 686.41) | 17.54 (7.07, 33.19) | 19.41 (7.97, 36.52) | 0.44(0.37,0.50) |
| Niger | 249.28 (93.99, 485.28) | 763.65 (283.49, 1447.5) | 12.41 (4.65, 24.32) | 11.8 (4.13, 22.42) | -0.09(-0.16,-0.02) |
| Nigeria | 3886.43 (2155.63, 6605.95) | 11452.08 (6356.24, 19324.49) | 15.98 (8.75, 27.11) | 17.05 (9.37, 28.76) | 0.53(0.39,0.66) |
| Niue | 0.08 (0.03, 0.15) | 0.06 (0.03, 0.12) | 15.96 (6.74, 30.12) | 17.76 (7.59, 33.12) | 0.43(0.37,0.50) |
| North Macedonia | 117.48 (53.51, 215.4) | 116.67 (50.54, 213.91) | 22.03 (9.95, 40.19) | 24.03 (10.74, 43.78) | 0.43(0.34,0.53) |
| Northern Mariana Islands | 3.74 (1.67, 6.8) | 2.24 (0.95, 4.25) | 24.42 (10.68, 44.76) | 21.61 (9.46, 40.54) | -0.61(-0.74,-0.48) |
| Norway | 669.83 (398.72, 1078.71) | 759.52 (455.01, 1222.64) | 60.26 (35.45, 97.37) | 63.06 (37.74, 102.07) | 0.11(-0.05,0.26) |
| Oman | 98.75 (45.92, 182.34) | 270.6 (120.52, 497.48) | 25.18 (11.46, 46.78) | 27.5 (12.2, 51.21) | 0.44(0.35,0.52) |
| Pakistan | 4181.68 (2050.2, 7394.52) | 11259.77 (5744.37, 19947.98) | 15.49 (7.49, 27.74) | 17.33 (8.64, 31.18) | 0.43(0.39,0.47) |
| Palau | 0.76 (0.32, 1.45) | 0.58 (0.23, 1.1) | 17.58 (7.29, 33.08) | 18.4 (7.64, 34.6) | 0.12(0.08,0.15) |
| Palestine | 78.34 (33.43, 147.56) | 233.86 (99.08, 446.45) | 14.73 (6.09, 27.98) | 16.23 (7.01, 31.22) | 0.21(0.15,0.28) |
| Panama | 162.73 (71.55, 294.84) | 367.19 (177.86, 652.06) | 23.07 (9.82, 42.31) | 32.38 (15.2, 57.96) | 1.16(1.10,1.22) |
| Papua New Guinea | 132.64 (50.57, 256.25) | 353.74 (139.63, 689.14) | 12.22 (4.74, 23.55) | 13.25 (5.34, 26.14) | 0.12(0.04,0.19) |
| Paraguay | 309.46 (144.99, 563.07) | 719.56 (328.3, 1306.52) | 28.36 (12.98, 52.79) | 34.39 (15.04, 62.9) | 0.69(0.61,0.76) |
| Peru | 1180.35 (516.37, 2212.99) | 2173.01 (981.36, 4026.89) | 18.88 (8.17, 36.12) | 22.56 (10.02, 41.7) | 0.82(0.74,0.90) |
| Philippines | 2568.68 (1412.52, 4357.57) | 4876.29 (2701.95, 8211.57) | 14.68 (8.03, 24.79) | 15.95 (8.65, 27.03) | 0.14(0.06,0.21) |
| Poland | 1986.85 (1058.46, 3456.17) | 3375.28 (2003.83, 5460.74) | 21.82 (11.61, 38) | 41.94 (24.5, 67.97) | 2.56(2.42,2.71) |
| Portugal | 1697.35 (898.98, 2834.93) | 1454.17 (737.19, 2474.78) | 60.62 (32.24, 102.11) | 67.07 (33.93, 113.07) | 0.16(0.04,0.28) |
| Puerto Rico | 277.26 (126.49, 509.69) | 217.04 (97.55, 396.83) | 27.91 (12.56, 52.11) | 30.71 (14, 55.62) | 0.29(0.21,0.37) |
| Qatar | 27.79 (13.08, 49.83) | 187.05 (85.65, 340.81) | 33.43 (15.52, 59.97) | 37.22 (16.93, 68.28) | 0.65(0.54,0.77) |
| Republic of Korea | 7045.22 (3397.31, 12278.17) | 6992.34 (3462.81, 12053.36) | 47.18 (22.52, 82.66) | 63.69 (31.89, 111.06) | 0.97(0.92,1.02) |
| Republic of Moldova | 313.59 (143.31, 577.94) | 246.04 (115.48, 436.1) | 26.91 (11.94, 49.37) | 31.65 (15.22, 56.55) | 0.77(0.51,1.03) |
| Romania | 1360.46 (600.9, 2527.44) | 1087.78 (485.76, 1966.92) | 23.26 (10.02, 43.21) | 30.13 (13.48, 54.51) | 1.09(0.95,1.22) |
| Russian Federation | 11716.83 (6434.38, 19513.17) | 10347.7 (5876.24, 17125.71) | 32.04 (17.37, 53.23) | 36.08 (20.19, 59.74) | 0.67(0.43,0.92) |
| Rwanda | 238.69 (94.49, 454.66) | 564.17 (226.24, 1089.34) | 12.63 (5, 24.3) | 14.6 (5.92, 28.42) | 0.66(0.48,0.83) |
| Saint Kitts and Nevis | 2.8 (1.24, 5.11) | 4.24 (1.94, 7.87) | 23.71 (10.28, 43.31) | 28.47 (13.2, 52.71) | 0.58(0.52,0.65) |
| Saint Lucia | 8.57 (3.72, 16.01) | 9.89 (4.28, 18.56) | 21.22 (8.96, 39.85) | 23.35 (10.28, 44.16) | 0.32(0.29,0.35) |
| Saint Vincent and the Grenadines | 6.26 (2.74, 11.62) | 6.25 (2.8, 11.76) | 19.76 (8.53, 36.86) | 23.01 (10.33, 43.2) | 0.52(0.47,0.58) |
| Samoa | 5.83 (2.49, 11.16) | 7.73 (3.01, 14.69) | 13.39 (5.64, 25.57) | 14.8 (5.78, 28.36) | 0.34(0.28,0.41) |
| San Marino | 5.26 (2.67, 9.18) | 4.84 (2.45, 8.33) | 74.04 (36.57, 130.14) | 71.6 (36.86, 123.86) | -0.07(-0.25,0.11) |
| Sao Tome and Principe | 4.59 (1.83, 8.76) | 10.2 (4.23, 19.23) | 14.96 (5.89, 28.88) | 16.46 (6.71, 30.85) | 0.47(0.40,0.53) |
| Saudi Arabia | 993.93 (440.09, 1839.95) | 2536.28 (1131.35, 4682.27) | 25.29 (10.69, 47.49) | 25.55 (11.24, 46.91) | 0.03(-0.12,0.17) |
| Senegal | 308.84 (131.56, 577.98) | 704.58 (290.94, 1319.28) | 15.22 (6.26, 28.89) | 16.03 (6.61, 29.72) | 0.14(0.09,0.18) |
| Serbia | 563.22 (254.07, 1082.95) | 505.58 (231.42, 929.22) | 23.73 (10.68, 45.84) | 26.36 (12.02, 49.41) | 0.67(0.50,0.84) |
| Seychelles | 4.11 (1.78, 7.69) | 5.26 (2.26, 10.03) | 19.5 (8.24, 36.89) | 23.28 (10.19, 44.43) | 0.51(0.45,0.57) |
| Sierra Leone | 157.05 (64.25, 291.8) | 340.63 (135.16, 647.44) | 13.76 (5.54, 25.58) | 13.02 (5.2, 25) | -0.13(-0.24,-0.01) |
| Singapore | 641.52 (323.57, 1113.17) | 1065.5 (541.91, 1828.16) | 60.06 (29.26, 105.26) | 79.78 (40.65, 137.79) | 0.90(0.84,0.95) |
| Slovakia | 340.23 (153.59, 632.38) | 325.33 (146.72, 595) | 25.18 (11.3, 47.18) | 29.31 (13.83, 53.6) | 0.69(0.61,0.77) |
| Slovenia | 139.07 (61.02, 258.9) | 109.52 (48.58, 196.3) | 27.24 (11.74, 50.45) | 29.96 (13.35, 53.18) | 0.44(0.38,0.49) |
| Solomon Islands | 10.09 (3.84, 18.92) | 20.82 (7.46, 41.48) | 11.36 (4.29, 21.31) | 11.67 (4.24, 23.2) | -0.06(-0.13,0.01) |
| Somalia | 165.28 (55.95, 330.15) | 430.69 (148.49, 868.91) | 9.01 (3.13, 17.94) | 7.77 (2.63, 15.98) | -0.40(-0.48,-0.32) |
| South Africa | 2352.13 (1230.13, 4023.22) | 3362.92 (1784.35, 5702.43) | 21.12 (10.86, 36.3) | 21.87 (11.62, 37.17) | 0.24(0.19,0.28) |
| South Sudan | 286.03 (120.97, 527.86) | 365.39 (146.23, 681.34) | 18.16 (7.55, 33.85) | 13.98 (5.54, 26.57) | -0.65(-0.80,-0.49) |
| Spain | 9697.11 (5298.12, 15890.35) | 9138.38 (4835.61, 15435.09) | 90.63 (49.17, 148.49) | 105.4 (56.53, 178.29) | 0.49(0.42,0.55) |
| Sri Lanka | 668.42 (251.79, 1295.4) | 1018.09 (417.49, 1887.58) | 13.91 (5.3, 26.9) | 18.91 (7.82, 35.09) | 1.06(1.02,1.10) |
| Sudan | 746.69 (302.51, 1410) | 1954.66 (763.1, 3696.67) | 13.9 (5.52, 26.43) | 15.81 (6.25, 30.11) | 0.55(0.50,0.59) |
| Suriname | 24.75 (10.91, 45.98) | 34.84 (15.61, 65.18) | 22.35 (9.35, 42.61) | 24.2 (10.98, 45.27) | 0.45(0.39,0.51) |
| Sweden | 1421.29 (771.71, 2393.81) | 1827.26 (1004.74, 3008.02) | 67.36 (36.44, 115.52) | 82.22 (44.22, 137.23) | 0.73(0.65,0.81) |
| Switzerland | 1931.77 (1156.06, 2993.13) | 2121.12 (1267.13, 3351.62) | 100.75 (60.32, 157.43) | 105.55 (63.06, 168.2) | 0.23(0.19,0.26) |
| Syrian Arab Republic | 527.89 (223.99, 968.51) | 655.11 (265.06, 1230.98) | 15.87 (6.63, 28.98) | 16.12 (6.43, 30.36) | 0.12(0.03,0.21) |
| Taiwan (Province of China) | 1175.58 (502.69, 2256.68) | 1193.76 (528.58, 2199.07) | 20.24 (8.47, 38.5) | 25.91 (11.85, 47.68) | 0.75(0.66,0.85) |
| Tajikistan | 291.93 (127.58, 540.7) | 482.9 (197.01, 907.93) | 19.67 (8.3, 36.94) | 17.83 (7.23, 33.53) | -0.07(-0.40,0.27) |
| Thailand | 2688.85 (1080.64, 5137.26) | 2832.48 (1150.79, 5245.98) | 15.51 (6.24, 30.12) | 20.44 (8.72, 37.58) | 0.81(0.76,0.86) |
| Timor-Leste | 26.29 (10.18, 50.86) | 63.68 (26.31, 121.72) | 12.84 (4.89, 25.36) | 15.73 (6.31, 30.29) | 0.91(0.77,1.05) |
| Togo | 134.51 (54.26, 266.38) | 312.79 (120.48, 592.2) | 13.62 (5.45, 27.57) | 13.75 (5.44, 25.89) | -0.04(-0.11,0.03) |
| Tokelau | 0.05 (0.02, 0.1) | 0.05 (0.02, 0.1) | 13.73 (5.34, 27.29) | 15.76 (6.51, 31.59) | 0.48(0.45,0.52) |
| Tonga | 3.47 (1.35, 6.66) | 3.87 (1.58, 7.31) | 13.45 (5.09, 26.05) | 14.61 (6.15, 27.69) | 0.18(0.13,0.24) |
| Trinidad and Tobago | 82.26 (35.72, 150.92) | 94.91 (41.92, 176.19) | 24.49 (10.52, 44.45) | 30.19 (13.33, 56.48) | 1.07(0.95,1.19) |
| Tunisia | 413.79 (180.64, 769.87) | 548.59 (218.03, 1043.61) | 17.67 (7.67, 33.05) | 19.99 (8.23, 37.88) | 0.40(0.34,0.46) |
| Turkey | 3005.58 (1338.27, 5565.89) | 4829.14 (2226.14, 8865.11) | 18.67 (8.15, 34.41) | 23.57 (10.86, 43.31) | 0.69(0.58,0.80) |
| Turkmenistan | 235.64 (100.26, 428.59) | 383.77 (177.24, 703.65) | 22.32 (9.26, 41.09) | 28.87 (13.37, 53.57) | 1.18(0.90,1.47) |
| Tuvalu | 0.28 (0.11, 0.55) | 0.4 (0.15, 0.78) | 11.62 (4.56, 23.14) | 13.32 (5.11, 25.73) | 0.31(0.23,0.38) |
| Uganda | 570.78 (226.33, 1114.73) | 1794.1 (765.99, 3335.56) | 12.25 (4.86, 24.69) | 14.48 (6.05, 27.18) | 0.67(0.62,0.71) |
| Ukraine | 3188.74 (1444.44, 5834.08) | 2068.87 (910.47, 3807.42) | 26.08 (12.09, 47.81) | 25.75 (11.87, 46.97) | 0.23(0.02,0.45) |
| United Arab Emirates | 143.83 (67.37, 259.88) | 402.1 (174.46, 734.5) | 36.61 (16.99, 66.6) | 31.06 (14.07, 55.39) | -0.57(-0.64,-0.50) |
| United Kingdom | 9308.62 (5229.42, 15138.23) | 11863.96 (6925.4, 19008.07) | 60.79 (33.99, 99) | 74.65 (43.65, 119.5) | 0.63(0.48,0.79) |
| United Republic of Tanzania | 962.93 (363.62, 1798.08) | 2651.28 (1116.38, 4880.82) | 13.39 (4.82, 25.01) | 15.68 (6.58, 29.22) | 0.68(0.59,0.77) |
| United States of America | 59116.99 (32897.14, 95641.15) | 66244.68 (38100, 106691.54) | 81.07 (45.35, 131.27) | 81.45 (46.22, 132.2) | 0.03(-0.01,0.06) |
| United States Virgin Islands | 7.98 (3.56, 15.01) | 5.23 (2.38, 9.57) | 28.4 (12.46, 53.87) | 33.3 (15.12, 60.84) | 0.50(0.32,0.69) |
| Uruguay | 324.45 (156.71, 577.81) | 395.01 (190.9, 703.99) | 40.25 (19.29, 72.97) | 46.69 (21.99, 83.84) | 0.41(0.33,0.50) |
| Uzbekistan | 1132.2 (501.5, 2127.1) | 2051.02 (883.07, 3798.01) | 19.4 (8.45, 36.88) | 23.2 (10.19, 43.17) | 0.75(0.59,0.91) |
| Vanuatu | 4.88 (1.81, 9.49) | 10.24 (3.86, 19.09) | 12.39 (4.53, 23.88) | 12.31 (4.57, 22.83) | 0.02(-0.02,0.05) |
| Venezuela | 1349.52 (611.42, 2469.83) | 1495.43 (668.92, 2789.56) | 24.37 (10.86, 45) | 23.55 (10.62, 43.82) | 0.13(0.03,0.22) |
| Viet Nam | 2197.2 (825.08, 4349.6) | 3775.41 (1487.36, 7107.49) | 11.34 (4.17, 22.56) | 16.46 (6.72, 30.75) | 1.26(1.23,1.28) |
| Yemen | 450.21 (184.27, 881.92) | 1253.53 (536, 2462.63) | 14.53 (5.81, 28.52) | 13.85 (5.99, 27) | 0.02(-0.11,0.15) |
| Zambia | 340.2 (141.96, 628.29) | 972.6 (401.04, 1825.51) | 15.01 (6.26, 27.63) | 16.93 (7.05, 31.86) | 0.67(0.51,0.82) |
| Zimbabwe | 506.29 (220.43, 951.74) | 696.9 (286.34, 1318.47) | 17.33 (7.54, 32.61) | 15.48 (6.3, 29.66) | -0.61(-0.76,-0.47) |

Abbreviations: DALYs, disability-adjusted life-years; AN, anorexia nervosa; WCBA, women of childbearing age; ASDR, age-standardized DALY rate; EAPC, estimated annual percentage change; UI, uncertainty interval; CI, confidence interval
