# Supplementary material for: Patterns and trends of eating disorders among women of childbearing age: a comprehensive analysis from 1990 to 2021 with future predictions
Source: Eat Weight Disord. 2026 Mar 23;31(1):40. doi: 10.1007/s40519-026-01842-8 (PMC13132930; doi:10.1007/s40519-026-01842-8)
Supplement: Supplementary file 15 — Supplementary Material 15. [file 40519_2026_1842_MOESM15_ESM.docx]

Table S5. DALYs of BN cases among WCBA in 1990 and 2021 at the national level, along with their EAPCs from 1990 to 2021.

| Location | DALYs | | | | |
| --- | --- | --- | --- | --- | --- |
|  | Number of cases(95% UI) | | ASDR per 100,000 population (95% UI) | | EAPC(95% CI) |
|  | 1990 | 2021 | 1990 | 2021 | 1990-2021 |
| Afghanistan | 1162.13 (481.10, 2229.13) | 4053.48 (1747.23, 7793.76) | 52.53 (22.83, 98.33) | 52.93 (23.20, 100.09) | 0.46 (0.16, 0.75) |
| Albania | 382.46 (161.08, 707.68) | 355.3 (157.02, 651.30) | 42.74 (17.91, 78.87) | 59.07 (26.07, 108.58) | 1.43 (1.30, 1.55) |
| Algeria | 5617.24 (2512.41, 10380.21) | 10891.38 (5035.85, 19780.73) | 89.97 (40.97, 164.36) | 98.52 (45.19, 179.91) | 0.57 (0.47, 0.67) |
| American Samoa | 7.52 (3.24, 14.18) | 5.83 (2.45, 11.01) | 58.49 (25.27, 109.39) | 51.98 (22.01, 97.59) | -0.29 (-0.32, -0.25) |
| Andorra | 32.60 (16.37, 57.2) | 40.61 (20.28, 70.04) | 215.36 (107.46, 379.68) | 229.78 (113.58, 401.97) | 0.32 (0.23, 0.41) |
| Angola | 1216.00 (508.63, 2318.96) | 5040.56 (2213.38, 9435.71) | 49.87 (21.07, 94.34) | 62.86 (27.84, 116.61) | 1.14 (0.98, 1.30) |
| Antigua and Barbuda | 21.63 (10.16, 39.05) | 33.61 (15.82, 60.28) | 125.00 (58.65, 225.55) | 144.69 (67.84, 260.68) | 0.47 (0.40, 0.54) |
| Argentina | 10452.99 (4866.84, 18893.86) | 17423.14 (8263.86, 31392.63) | 130.01 (60.63, 234.56) | 149.32 (70.59, 269.95) | 0.47 (0.41, 0.54) |
| Armenia | 419.51 (177.50, 784.09) | 394.75 (179.95, 722.54) | 45.20 (18.84, 85.09) | 55.37 (24.85, 102.50) | 1.23 (0.92, 1.53) |
| Australia | 13596.87 (6691.06, 23988.67) | 25169.48 (14545.72, 40238.15) | 307.27 (150.69, 543.49) | 438.09 (252.49, 702.94) | 1.18 (1.05, 1.30) |
| Austria | 3906.32 (1925.01, 6782.15) | 4670.63 (2300.90, 8050.33) | 197.58 (97.23, 344.48) | 247.95 (121.01, 430.63) | 0.80 (0.73, 0.86) |
| Azerbaijan | 1104.19 (473.72, 2048.43) | 1768.22 (796.08, 3278.49) | 53.64 (22.85, 99.35) | 64.80 (28.69, 121.39) | 1.31 (0.82, 1.80) |
| Bahamas | 123.78 (58.16, 221.72) | 163.88 (76.35, 292.68) | 158.97 (74.74, 284.06) | 156.86 (73.12, 280.03) | 0.10 (0.01, 0.18) |
| Bahrain | 152.12 (70.68, 277.85) | 452.32 (218.96, 824.94) | 119.95 (55.72, 219.37) | 139.90 (67.45, 256.40) | 0.64 (0.58, 0.71) |
| Bangladesh | 10008.05 (4165.85, 19137.77) | 25164.67 (10705.24, 46816.03) | 37.92 (15.92, 71.58) | 53.66 (22.87, 99.66) | 1.15 (1.04, 1.27) |
| Barbados | 90.97 (42.16, 159.25) | 83.51 (38.81, 148.48) | 127.27 (58.74, 223.31) | 125.08 (58.08, 222.73) | -0.06 (-0.20, 0.07) |
| Belarus | 1472.91 (639.77, 2735.46) | 1303.46 (572.85, 2353.35) | 57.26 (24.59, 107.10) | 66.89 (28.92, 122.68) | 0.91 (0.67, 1.16) |
| Belgium | 3548.90 (1723.38, 6301.19) | 4129.35 (2010.95, 7216.58) | 146.80 (70.94, 261.95) | 175.54 (84.81, 309.42) | 0.63 (0.56, 0.71) |
| Belize | 40.85 (18.73, 74.56) | 128.13 (59.04, 232.97) | 88.11 (40.70, 158.84) | 102.77 (47.49, 186.34) | 0.48 (0.39, 0.57) |
| Benin | 482.47 (205.05, 904.08) | 1597.08 (684.27, 2993.73) | 40.79 (17.46, 76.09) | 46.43 (19.98, 86.17) | 0.53 (0.48, 0.57) |
| Bermuda | 29.27 (14.10, 52.39) | 23.86 (11.45, 41.82) | 169.48 (80.94, 305.32) | 198.37 (94.64, 349.77) | 0.67 (0.58, 0.76) |
| Bhutan | 66.72 (27.64, 125.63) | 149.34 (64.37, 281.93) | 43.62 (18.33, 81.17) | 69.96 (30.04, 132.26) | 1.61 (1.59, 1.64) |
| Bolivia | 1865.70 (861.01, 3411.68) | 4809.77 (2278.23, 8637.98) | 114.98 (53.24, 209.41) | 149.85 (70.98, 269.34) | 0.96 (0.92, 0.99) |
| Bosnia and Herzegovina | 395.55 (158.19, 755.64) | 401.22 (173.06, 741.47) | 33.31 (13.26, 63.77) | 59.40 (25.56, 110.55) | 2.26 (2.04, 2.48) |
| Botswana | 212.15 (92.29, 405.36) | 577.86 (268.18, 1075.72) | 61.70 (27.05, 116.38) | 83.45 (38.54, 155.78) | 1.02 (0.99, 1.05) |
| Brazil | 35420.77 (16955.59, 63681.19) | 57134.42 (28392.16, 100546.56) | 85.71 (41.20, 153.20) | 100.52 (49.72, 178.07) | 0.66 (0.60, 0.72) |
| Brunei Darussalam | 134.34 (63.95, 241.21) | 204.50 (98.63, 363.78) | 183.34 (86.93, 328.83) | 165.89 (79.65, 297.23) | -0.16 (-0.22, -0.09) |
| Bulgaria | 1125.60 (487.62, 2135.45) | 881.23 (388.29, 1607.25) | 56.85 (24.61, 108.29) | 69.43 (30.20, 128.30) | 0.88 (0.70, 1.06) |
| Burkina Faso | 787.32 (318.73, 1521.79) | 2474.60 (996.17, 4678.52) | 35.76 (14.65, 68.44) | 42.39 (17.23, 79.52) | 0.60 (0.55, 0.65) |
| Burundi | 481.18 (193.58, 919.12) | 1072.93 (424.03, 2088.28) | 35.53 (14.33, 67.59) | 32.04 (12.66, 61.92) | -0.32 (-0.38, -0.26) |
| Cabo Verde | 38.67 (16.61, 73.83) | 99.58 (44.31, 184.18) | 44.68 (19.24, 84.49) | 64.06 (28.35, 118.67) | 1.38 (1.30, 1.46) |
| Cambodia | 628.71 (232.36, 1248.00) | 1622.90 (662.86, 3128.90) | 23.64 (8.75, 46.66) | 34.87 (14.17, 67.39) | 1.41 (1.29, 1.53) |
| Cameroon | 1263.29 (531.95, 2367.00) | 4210.19 (1868.45, 7851.15) | 49.86 (21.14, 92.35) | 50.84 (22.63, 94.29) | 0.21 (0.11, 0.31) |
| Canada | 13259.30 (6296.20, 23129.94) | 14491.25 (7091.89, 25490.91) | 182.39 (85.57, 320.55) | 185.38 (89.96, 328.48) | 0.14 (0.11, 0.16) |
| Central African Republic | 243.92 (98.26, 472.35) | 469.77 (188.01, 917.13) | 35.72 (14.49, 68.69) | 32.31 (13.00, 62.40) | -0.32 (-0.35, -0.28) |
| Chad | 509.46 (217.97, 946.75) | 1781.75 (738.18, 3331.24) | 36.30 (15.65, 66.86) | 43.39 (18.28, 80.26) | 0.79 (0.68, 0.90) |
| Chile | 4394.03 (2099.26, 7930.12) | 7391.45 (3513.95, 13293.96) | 116.49 (55.69, 209.99) | 160.21 (75.70, 290.22) | 1.07 (1.03, 1.10) |
| China | 88266.79 (40969.27, 160813.86) | 131940.89 (62425.34, 231872.34) | 26.00 (12.18, 47.06) | 44.76 (20.70, 80.24) | 1.84 (1.69, 1.98) |
| Colombia | 8535.19 (3910.16, 15715.80) | 14867.34 (6884.48, 26880.69) | 90.83 (41.70, 166.61) | 112.38 (51.93, 203.86) | 0.75 (0.65, 0.85) |
| Comoros | 54.07 (22.75, 101.26) | 93.74 (39.94, 177.89) | 48.66 (20.72, 89.81) | 46.89 (20.02, 88.59) | -0.06 (-0.12, -0.01) |
| Congo | 320.72 (137.60, 594.74) | 862.22 (367.57, 1595.69) | 53.49 (23.22, 98.29) | 59.12 (25.32, 108.88) | 0.52 (0.40, 0.63) |
| Cook Islands | 2.43 (1.04, 4.49) | 2.81 (1.21, 5.35) | 51.10 (21.96, 93.68) | 66.90 (29.15, 127.38) | 0.76 (0.73, 0.80) |
| Costa Rica | 845.40 (402.20, 1535.11) | 1547.50 (723.90, 2775.48) | 100.42 (47.68, 181.91) | 119.81 (55.78, 215.93) | 0.62 (0.60, 0.64) |
| Cote d'Ivoire | 1490.87 (673.56, 2799.81) | 3761.40 (1653.55, 7098.33) | 50.17 (22.68, 93.17) | 53.61 (23.68, 100.68) | 0.17 (0.03, 0.30) |
| Croatia | 731.64 (317.46, 1340.24) | 608.59 (270.42, 1103.38) | 62.08 (26.74, 114.52) | 73.03 (32.30, 133.57) | 0.82 (0.72, 0.92) |
| Cuba | 3266.90 (1526.67, 5894.72) | 2429.52 (1128.41, 4329.25) | 101.77 (47.69, 183.42) | 104.11 (48.27, 186.00) | 0.45 (0.27, 0.64) |
| Cyprus | 296.19 (141.65, 519.56) | 598.43 (297.77, 1052.49) | 150.25 (71.70, 264.27) | 179.68 (88.05, 320.39) | 0.61 (0.52, 0.71) |
| Czechia | 1662.66 (739.07, 3033.50) | 1642.33 (735.73, 3014.47) | 68.22 (30.28, 124.98) | 81.48 (36.37, 152.20) | 0.74 (0.67, 0.81) |
| Democratic People's Republic of Korea | 1533.18 (581.77, 3017.16) | 1554.69 (592.56, 3090.87) | 26.55 (10.13, 51.94) | 24.34 (9.30, 48.48) | -0.23 (-0.34, -0.12) |
| Democratic Republic of the Congo | 3677.43 (1497.61, 7049.26) | 7465.10 (2895.02, 14410.93) | 40.50 (16.62, 76.94) | 33.15 (12.99, 63.50) | -0.52 (-0.83, -0.20) |
| Denmark | 2166.50 (1028.24, 3809.45) | 2419.42 (1209.80, 4327.65) | 171.56 (81.32, 302.92) | 198.70 (99.17, 357.05) | 0.54 (0.48, 0.61) |
| Djibouti | 55.39 (22.71, 106.85) | 176.93 (78.73, 323.81) | 53.24 (22.17, 101.32) | 54.26 (24.03, 99.75) | 0.08 (-0.07, 0.23) |
| Dominica | 17.84 (8.15, 32.25) | 18.26 (8.39, 32.81) | 98.83 (45.41, 177.42) | 111.59 (51.28, 200.56) | 0.45 (0.40, 0.49) |
| Dominican Republic | 1901.78 (876.21, 3446.43) | 3802.58 (1777.08, 6838.99) | 92.29 (42.72, 166.30) | 128.31 (59.94, 230.88) | 1.16 (1.13, 1.19) |
| Ecuador | 4047.27 (1914.26, 7333.67) | 8322.54 (3954.46, 14749.09) | 149.26 (71.04, 268.42) | 172.86 (81.97, 306.82) | 0.68 (0.59, 0.77) |
| Egypt | 9496.69 (4229.67, 17727.54) | 24971.28 (11415.63, 45311.12) | 69.65 (31.14, 128.86) | 94.05 (43.02, 170.30) | 1.09 (1.04, 1.15) |
| El Salvador | 1108.04 (501.54, 2068.25) | 1744.82 (815.36, 3185.11) | 78.73 (35.85, 145.72) | 95.80 (44.79, 174.92) | 0.67 (0.63, 0.71) |
| Equatorial Guinea | 42.01 (16.95, 79.11) | 359.50(159.54, 644.16) | 40.64 (16.68, 76.05) | 93.64 (41.86, 166.73) | 3.77 (3.10, 4.44) |
| Eritrea | 294.17 (118.21, 563.75) | 728.98 (307.31, 1370.53) | 35.42 (14.32, 67.21) | 42.25 (17.83, 79.07) | 0.39 (0.22, 0.57) |
| Estonia | 212.78 (91.62, 390.13) | 200.89 (92.27, 371.08) | 57.05 (24.39, 105.18) | 77.85 (35.23, 145.71) | 1.30 (1.18, 1.41) |
| Eswatini | 121.71 (53.26, 228.70) | 228.18 (101.99, 414.20) | 58.64 (25.91, 109.29) | 68.48 (30.76, 123.91) | 0.44 (0.41, 0.48) |
| Ethiopia | 3856.96 (1709.13, 7143.40) | 12858.96 (5872.16, 23503.36) | 31.71 (14.19, 58.12) | 43.45 (20.08, 78.70) | 1.23 (0.98, 1.47) |
| Fiji | 78.68 (31.99, 148.83) | 103.95 (44.39, 200.19) | 38.57 (15.70, 72.66) | 45.55 (19.44, 87.77) | 0.50 (0.46, 0.53) |
| Finland | 2154.15 (1060.10, 3822.51) | 2225.57 (1076.16, 3936.07) | 178.64 (87.60, 320.20) | 204.66 (98.49, 364.15) | 0.51 (0.43, 0.60) |
| France | 26577.73 (13204.96, 46447.62) | 29118.37 (14214.60, 51159.56) | 185.15 (91.69, 324.50) | 216.99 (105.44, 382.78) | 0.52 (0.48, 0.55) |
| Gabon | 190.41 (85.08, 351.92) | 420.71 (189.21, 765.51) | 80.20 (36.47, 146.41) | 82.60 (37.38, 149.25) | 0.10 (0.08, 0.12) |
| Gambia | 113.50 (47.83, 211.53) | 292.80 (126.35, 557.76) | 45.75 (19.50, 84.52) | 44.64 (19.39, 84.31) | -0.05 (-0.09, -0.01) |
| Georgia | 797.52 (357.39, 1458.91) | 424.41 (187.44, 787.60) | 56.63 (25.20, 103.80) | 57.09 (24.79, 107.44) | 0.65 (0.20, 1.10) |
| Germany | 26381.82 (12874.39, 46253.90) | 25496.63 (12831.48, 44680.43) | 136.20 (66.18, 239.98) | 157.72 (78.52, 279.75) | 0.50 (0.46, 0.55) |
| Ghana | 1680.55 (709.19, 3214.96) | 5536.26 (2508.67, 10166.92) | 44.60 (18.96, 84.60) | 58.02 (26.25, 106.32) | 0.89 (0.80, 0.98) |
| Greece | 4072.07 (2024.25, 7180.60) | 3475.93 (1705.61, 6085.96) | 164.19 (81.60, 289.85) | 178.53 (86.68, 316.62) | 0.35 (0.22, 0.49) |
| Greenland | 33.21 (15.98, 57.95) | 28.48 (14.13, 50.46) | 208.77 (100.43, 366.67) | 221.79 (109.40, 395.07) | 0.41 (0.32, 0.49) |
| Grenada | 19.64 (9.12, 36.05) | 31.62 (14.68, 56.76) | 93.75 (43.69, 171.13) | 122.00 (56.63, 219.43) | 0.97 (0.89, 1.04) |
| Guam | 28.17 (12.47, 51.79) | 27.47 (12.42, 50.84) | 76.36 (33.77, 140.47) | 77.93 (35.32, 144.22) | 0.19 (0.14, 0.23) |
| Guatemala | 1638.40 (735.21, 3018.56) | 4381.23 (2070.31, 7868.81) | 83.63 (37.75, 152.89) | 94.70 (44.68, 169.63) | 0.47 (0.44, 0.50) |
| Guinea | 600.60 (261.21, 1135.27) | 1576.43 (668.96, 2969.03) | 41.99 (18.29, 79.06) | 44.74 (19.06, 83.76) | 0.09 (-0.02, 0.20) |
| Guinea-Bissau | 96.81 (39.85, 183.22) | 229.80 (94.74, 436.80) | 39.09 (16.16, 73.49) | 41.20 (17.03, 77.81) | 0.07 (0.01, 0.12) |
| Guyana | 175.00 (77.13, 326.92) | 223.38 (102.56, 403.55) | 78.59 (34.84, 145.59) | 105.97 (48.79, 191.46) | 0.94 (0.90, 0.97) |
| Haiti | 1087.03 (485.59, 1995.08) | 2277.61 (999.91, 4245.12) | 66.06 (29.53, 120.80) | 62.53 (27.41, 116.50) | -0.07 (-0.11, -0.03) |
| Honduras | 855.14 (383.87, 1577.57) | 2451.89 (1079.15, 4490.50) | 75.34 (34.07, 137.86) | 81.70 (36.14, 149.19) | 0.26 (0.23, 0.30) |
| Hungary | 1677.90 (726.95, 3089.94) | 1626.57 (758.70, 2956.27) | 69.77 (30.07, 129.48) | 85.34 (39.59, 156.92) | 0.75 (0.70, 0.80) |
| Iceland | 114.39 (54.47, 205.27) | 157.48 (77.90, 275.80) | 172.99 (82.32, 310.59) | 203.37 (100.31, 357.95) | 0.59 (0.54, 0.64) |
| India | 83267.57 (38955.81, 150975.28) | 243030.37 (117876.92, 431551.97) | 39.47 (18.60, 71.01) | 63.16 (30.69, 112.04) | 1.69 (1.62, 1.76) |
| Indonesia | 16808.68 (7800.09, 30782.60) | 35455.79 (16783.90, 63685.57) | 33.17 (15.48, 60.20) | 47.94 (22.64, 86.47) | 1.15 (1.05, 1.26) |
| Iran (Islamic Republic of) | 13196.62 (6273.63, 23865.08) | 26283.58 (13018.99, 46216.69) | 97.19 (46.84, 173.36) | 118.03 (57.54, 210.85) | 0.87 (0.76, 0.97) |
| Iraq | 3705.32 (1638.34, 6751.63) | 11042.33 (5082.12, 19796.59) | 85.71 (38.53, 153.91) | 102.08 (47.12, 182.54) | 1.09 (0.87, 1.30) |
| Ireland | 1182.15 (555.54, 2133.45) | 2192.04 (1067.82, 3860.06) | 133.68 (62.94, 240.75) | 203.49 (98.56, 360.94) | 1.38 (1.24, 1.52) |
| Israel | 1542.68 (744.54, 2721.04) | 3476.83 (1695.64, 6132.83) | 125.97 (60.77, 222.07) | 159.97 (78.01, 282.15) | 0.83 (0.75, 0.91) |
| Italy | 41332.77 (21390.91, 70910.58) | 34118.55 (17978.34, 57418.17) | 293.16 (151.62, 503.94) | 310.21 (162.55, 526.19) | 0.20 (0.15, 0.26) |
| Jamaica | 625.29 (282.18, 1159.89) | 819.21 (376.86, 1468.92) | 96.12 (43.62, 176.78) | 103.50 (47.43, 186.21) | 0.27 (0.23, 0.32) |
| Japan | 37705.52 (18569.21, 67182.26) | 31269.66 (15549.82, 54496.30) | 126.60 (62.25, 226.30) | 143.27 (70.77, 252.83) | 0.32 (0.27, 0.36) |
| Jordan | 680.66 (297.71, 1270.20) | 2781.94 (1243.49, 5122.95) | 75.89 (33.95, 139.30) | 88.10 (39.63, 161.08) | 0.82 (0.68, 0.96) |
| Kazakhstan | 2510.29 (1084.22, 4683.90) | 3389.77 (1543.29, 6168.14) | 58.16 (24.96, 108.72) | 73.31 (32.87, 134.75) | 1.21 (0.93, 1.49) |
| Kenya | 2602.95 (1195.45, 4797.39) | 7329.06 (3483.50, 13153.64) | 46.33 (21.72, 83.59) | 52.20 (25.08, 92.48) | 0.39 (0.29, 0.48) |
| Kiribati | 5.67 (2.20, 10.79) | 9.39 (3.82, 18.39) | 28.37 (11.02, 54.00) | 28.54 (11.56, 55.83) | 0.01 (-0.03, 0.05) |
| Kuwait | 631.85 (302.86, 1120.15) | 2211.07 (1050.65, 3837.81) | 140.64 (67.42, 249.26) | 158.38 (74.15, 280.79) | 0.55 (0.43, 0.68) |
| Kyrgyzstan | 516.17 (220.95, 976.85) | 760.35 (322.07, 1441.09) | 45.51 (19.39, 86.09) | 43.08 (18.10, 81.98) | -0.01 (-0.35, 0.33) |
| Lao People's Democratic Republic | 261.32 (97.94, 514.98) | 855.55 (348.95, 1620.06) | 25.67 (9.64, 50.11) | 41.67 (16.96, 78.86) | 1.66 (1.55, 1.77) |
| Latvia | 376.17 (167.63, 702.77) | 266.06 (121.09, 474.91) | 59.49 (26.39, 111.69) | 74.48 (33.61, 134.66) | 1.11 (0.93, 1.29) |
| Lebanon | 650.15 (289.48, 1208.49) | 1419.81 (669.41, 2549.59) | 84.10 (37.72, 155.57) | 96.80 (45.02, 175.24) | 0.75 (0.65, 0.84) |
| Lesotho | 150.78 (64.78, 288.55) | 265.77 (114.73, 498.69) | 38.67 (16.67, 73.49) | 49.62 (21.45, 92.71) | 0.81 (0.80, 0.83) |
| Liberia | 229.14 (93.11, 444.25) | 508.67 (211.33, 995.68) | 38.17 (15.59, 73.32) | 35.22 (14.72, 68.35) | 0.19 (-0.01, 0.39) |
| Libya | 1212.91 (568.76, 2229.52) | 1663.28 (760.55, 3037.86) | 124.77 (59.57, 225.73) | 86.02 (39.25, 157.74) | -0.73 (-0.99, -0.46) |
| Lithuania | 538.46 (237.04, 1008.23) | 419.93 (196.80, 766.14) | 58.75 (25.71, 110.46) | 77.82 (36.24, 143.08) | 1.28 (1.12, 1.45) |
| Luxembourg | 186.43 (91.31, 326.37) | 368.27 (187.17, 642.67) | 192.62 (93.93, 339.66) | 249.70 (125.94, 440.46) | 0.84 (0.75, 0.93) |
| Madagascar | 1148.17 (481.56, 2196.42) | 3083.09 (1259.39, 5798.36) | 39.88 (16.84, 75.49) | 40.54 (16.68, 75.37) | 0.16 (0.10, 0.22) |
| Malawi | 832.16 (334, 1615.34) | 1965.11 (770.10, 3801.05) | 34.27 (13.83, 65.88) | 36.88 (14.48, 70.37) | 0.32 (0.28, 0.37) |
| Malaysia | 2105.03 (887.86, 3976.71) | 5485.30 (2515.74, 10078.22) | 44.67 (18.85, 84.12) | 63.58 (29.01, 117.09) | 1.13 (1.09, 1.16) |
| Maldives | 19.13 (7.70, 37.75) | 62.22 (27.00, 115.99) | 37.36 (15.23, 72.62) | 53.72 (22.95, 101.46) | 1.30 (1.25, 1.34) |
| Mali | 705.39 (275.92, 1351.60) | 2590.76 (1110.16, 5013.84) | 34.79 (13.76, 66.18) | 44.58 (19.19, 85.14) | 0.86 (0.83, 0.89) |
| Malta | 122.43 (58.69, 218.99) | 165.70 (81.97, 289.65) | 134.87 (64.34, 243.05) | 187.65 (92.10, 331.83) | 1.03 (0.97, 1.09) |
| Marshall Islands | 3.31 (1.33, 6.49) | 5.03 (2.01, 9.77) | 31.54 (12.69, 61.43) | 33.77 (13.52, 65.32) | 0.12 (0.08, 0.16) |
| Mauritania | 232.98 (100.18, 427.92) | 600.74 (257.34, 1134.89) | 46.70 (20.25, 85.29) | 53.42 (23.21, 99.89) | 0.53 (0.46, 0.60) |
| Mauritius | 134.08 (55.71, 255.78) | 180.14 (77.13, 332.77) | 42.65 (17.68, 81.39) | 58.83 (25.19, 109.06) | 0.95 (0.90, 1.00) |
| Mexico | 29879.98 (14727.09, 53050.54) | 44433.53 (22235.19, 78372.99) | 126.49 (62.92, 222.31) | 128.57 (64.31, 227.12) | 0.04 (0.03, 0.06) |
| Micronesia (Federated States of) | 7.63 (3.09, 14.92) | 8.81 (3.51, 17.14) | 31.03 (12.63, 60.23) | 33.16 (13.35, 64.02) | 0.20 (0.18, 0.22) |
| Monaco | 20.89 (10.47, 35.29) | 25.28 (13.18, 43.17) | 320.07 (159.09, 546.77) | 386.61 (200.84, 663.55) | 0.63 (0.60, 0.65) |
| Mongolia | 228.32 (94.54, 433.37) | 489.06 (218.29, 902.52) | 40.99 (16.97, 77.12) | 58.86 (25.99, 109.39) | 1.52 (1.32, 1.73) |
| Montenegro | 94.12 (41.10, 174.15) | 89.51 (40.35, 165.81) | 59.68 (26.01, 110.55) | 66.09 (29.69, 123.22) | 0.75 (0.54, 0.95) |
| Morocco | 4456.66 (1932.40, 8217.10) | 7999.42 (3605.03, 14727.68) | 66.89 (29.25, 122.78) | 83.29 (37.50, 153.61) | 0.78 (0.70, 0.86) |
| Mozambique | 907.41 (343.01, 1785.24) | 2997.61 (1226.92, 5702.37) | 27.58 (10.50, 53.90) | 37.12 (15.46, 70.08) | 1.22 (1.14, 1.30) |
| Myanmar | 2502.37 (922.96, 4955.08) | 6071.99 (2536.25, 11460.67) | 22.49 (8.26, 44.37) | 40.30 (16.89, 75.91) | 2.19 (2.02, 2.37) |
| Namibia | 221.43 (96.94, 414.28) | 509.59 (229.72, 941.69) | 61.28 (27.16, 113.21) | 74.17 (33.50, 136.53) | 0.80 (0.70, 0.89) |
| Nauru | 1.42 (0.62, 2.70) | 1.44 (0.62, 2.76) | 55.86 (24.50, 105.32) | 49.11 (20.93, 93.44) | -0.38 (-0.84, 0.08) |
| Nepal | 1705.93 (706.50, 3224.03) | 4582.73 (1930.90, 8582.50) | 35.51 (14.80, 66.69) | 48.63 (20.55, 90.70) | 1.00 (0.94, 1.07) |
| Netherlands | 3818.59 (2030.48, 6341.10) | 4651.25 (2323.65, 8085.29) | 96.56 (51.31, 160.59) | 131.31 (65.51, 229.28) | 1.20 (1.11, 1.28) |
| New Zealand | 2377.79 (1150.78, 4234.00) | 3061.05 (1492.62, 5396.22) | 262.42 (126.90, 467.54) | 264.66 (128.00, 469.52) | 0.09 (0.06, 0.12) |
| Nicaragua | 763.78 (337.84, 1425.60) | 1551.57 (711.52, 2876.61) | 77.46 (34.41, 143.67) | 83.32 (38.16, 154.49) | 0.44 (0.36, 0.52) |
| Niger | 645.83 (253.26, 1231.51) | 2002.65 (814.12, 3934.25) | 34.96 (13.87, 66.10) | 35.17 (14.46, 68.07) | 0.11 (0.01, 0.22) |
| Nigeria | 11277.10 (5314.09, 20609.8)0 | 36459.07 (17082.91, 66045.22) | 51.59 (24.74, 92.51) | 60.17 (28.63, 107.13) | 0.94 (0.74, 1.15) |
| Niue | 0.21 (0.08, 0.39) | 0.18 (0.08, 0.35) | 42.05 (17.32, 78.54) | 49.61 (20.90, 94.16) | 0.71 (0.63, 0.80) |
| North Macedonia | 270.59 (113.92, 502.75) | 310.28 (138.99, 566.15) | 53.04 (22.30, 98.68) | 62.14 (27.66, 114.78) | 0.73 (0.61, 0.85) |
| Northern Mariana Islands | 10.95 (4.87, 20.29) | 6.80 (3.00, 12.40) | 72.99 (32.37, 135.19) | 64.77 (28.73, 117.76) | -0.62 (-0.75, -0.50) |
| Norway | 2023.48 (1003.60, 3519.85) | 2699.52 (1377.26, 4642.71) | 194.92 (96.41, 340.42) | 233.19 (118.20, 403.55) | 0.70 (0.62, 0.77) |
| Oman | 406.03 (190.00, 754.66) | 1388.56 (669.93, 2489.21) | 113.22 (53.28, 208.47) | 135.10 (64.48, 244.44) | 0.88 (0.75, 1.01) |
| Pakistan | 11797.50 (5431.31, 21623.20) | 37853.70 (17237.45, 68400.31) | 48.08 (22.44, 87.03) | 59.93 (27.41, 107.83) | 0.78 (0.72, 0.84) |
| Palau | 2.03 (0.87, 3.87) | 1.76 (0.76, 3.23) | 47.80 (20.45, 90.70) | 52.85 (22.95, 97.10) | 0.31 (0.27, 0.34) |
| Palestine | 273.74 (112.13, 521.74) | 955.40 (427.68, 1756.90) | 57.61 (24.08, 108.22) | 69.66 (31.33, 127.25) | 0.60 (0.54, 0.66) |
| Panama | 650.45 (297.91, 1194.69) | 1451.90 (680.37, 2597.11) | 99.63 (45.82, 182.02) | 135.27 (63.48, 242.01) | 1.10 (1.03, 1.17) |
| Papua New Guinea | 308.58 (119.41, 609.56) | 927.34 (389.57, 1771.26) | 30.22 (11.79, 59.30) | 34.27 (14.39, 65.43) | 0.30 (0.22, 0.37) |
| Paraguay | 764.60 (339.21, 1417.24) | 1797.09 (810.05, 3247.03) | 76.25 (33.93, 140.33) | 91.88 (41.39, 166.08) | 0.61 (0.53, 0.68) |
| Peru | 8018.68 (3767.04, 14583.43) | 17774.15 (8708.65, 31650.50) | 137.47 (64.72, 248.42) | 182.71 (89.22, 326.11) | 0.89 (0.77, 1.01) |
| Philippines | 5628.31 (2590.43, 10244.05) | 13193.97 (6136.87, 24091.31) | 34.29 (15.96, 61.74) | 43.93 (20.51, 79.91) | 0.76 (0.67, 0.85) |
| Poland | 5113.98 (2447.25, 9210.32) | 6377.58 (3074.12, 11285.53) | 55.16 (26.15, 100.27) | 79.19 (37.51, 143.00) | 1.34 (1.30, 1.39) |
| Portugal | 3302.72 (1548.60, 5952.19) | 3439.44 (1666.75, 6049.94) | 131.92 (61.86, 237.79) | 164.50 (79.33, 292.20) | 0.81 (0.65, 0.96) |
| Puerto Rico | 1364.41 (652.25, 2440.73) | 1187.45 (568.77, 2110.16) | 142.87 (68.36, 255.40) | 167.55 (80.11, 298.79) | 0.56 (0.46, 0.67) |
| Qatar | 132.36 (64.52, 234.59) | 1093.09 (548.25, 1911.33) | 159.91 (77.20, 285.20) | 197.78 (97.14, 351.00) | 1.16 (1.00, 1.31) |
| Republic of Korea | 12115.86 (5641.30, 22414.57) | 14435.44 (6714.65, 25229.04) | 91.26 (42.41, 168.64) | 137.18 (63.31, 242.23) | 1.31 (1.24, 1.38) |
| Republic of Moldova | 517.35 (215.94, 976.59) | 391.21 (166.55, 728.71) | 45.38 (18.75, 86.17) | 47.79 (20.15, 90.60) | 0.44 (0.14, 0.75) |
| Romania | 2788.84 (1146.54, 5283.87) | 2388.75 (1055.36, 4430.83) | 49.95 (20.51, 94.45) | 65.69 (28.72, 122.52) | 1.21 (1.02, 1.40) |
| Russian Federation | 25475.10 (12317.64, 44818.81) | 22994.85 (11191.77, 40085.42) | 68.07 (32.53, 121.14) | 74.67 (35.47, 132.74) | 0.69 (0.42, 0.97) |
| Rwanda | 622.58 (255.06, 1209.96) | 1617.55 (654.65, 3077.84) | 35.76 (14.72, 68.98) | 44.05 (17.99, 83.22) | 0.94 (0.74, 1.15) |
| Saint Kitts and Nevis | 12.96 (5.91, 23.54) | 22.76 (10.67, 39.71) | 118.27 (53.90, 214.18) | 150.67 (70.51, 264.06) | 0.86 (0.78, 0.95) |
| Saint Lucia | 37.79 (17.27, 69.74) | 51.28 (23.62, 92.52) | 101.90 (46.94, 186.85) | 117.41 (53.96, 212.79) | 0.50 (0.47, 0.54) |
| Saint Vincent and the Grenadines | 26.47 (12.31, 48.19) | 30.66 (14.05, 55.37) | 91.70 (42.70, 166.14) | 113.62 (52.14, 205.40) | 0.78 (0.70, 0.86) |
| Samoa | 13.30 (5.33, 25.40) | 19.50 (7.98, 37.35) | 34.24 (13.79, 64.69) | 38.95 (15.98, 74.26) | 0.52 (0.47, 0.57) |
| San Marino | 12.59 (6.11, 22.60) | 13.54 (6.70, 23.60) | 200.42 (97.26, 360.47) | 210.34 (103.42, 369.86) | 0.27 (0.05, 0.48) |
| Sao Tome and Principe | 12.02 (5.01, 23.30) | 29.60 (12.56, 57.25) | 44.15 (18.68, 84.62) | 51.27 (21.98, 98.39) | 0.68 (0.58, 0.79) |
| Saudi Arabia | 3658.12 (1671.87, 6750.20) | 12151.43 (5872.07, 21417.54) | 105.09 (48.75, 192.22) | 120.57 (57.94,214.07) | 0.65 (0.55, 0.75) |
| Senegal | 834.05 (350.01, 1587.22) | 2064.81 (870.51, 3876.74) | 45.64 (19.46, 85.71) | 50.34 (21.52, 93.86) | 0.35 (0.29, 0.42) |
| Serbia | 1306.10 (575.49, 2373.38) | 1229.03 (544.82, 2285.64) | 56.82 (24.97, 103.61) | 64.59 (28.50, 121.30) | 0.77 (0.57, 0.98) |
| Seychelles | 9.73 (4.14, 18.26) | 14.68 (6.53, 27.49) | 50.04 (21.37, 93.29) | 63.31 (28.03, 118.98) | 0.71 (0.64, 0.77) |
| Sierra Leone | 433.75 (180.12, 830.55) | 960.87 (398.89, 1862.72) | 39.81 (16.51, 75.85) | 39.43 (16.44, 75.58) | 0.01 (-0.16, 0.17) |
| Singapore | 1159.41 (542.98, 2106.73) | 2300.50 (1159.54, 4030.23) | 120.17 (55.98, 219.42) | 175.84 (86.99, 314.53) | 1.30 (1.25, 1.35) |
| Slovakia | 813.16 (345.61, 1507.19) | 901.89 (418.40, 1643.18) | 61.98 (26.22, 115.32) | 78.39 (35.92, 144.94) | 1.03 (0.93, 1.14) |
| Slovenia | 337.18 (147.83, 633.33) | 305.18 (140.30, 552.97) | 68.29 (29.80, 128.80) | 80.59 (36.86, 148.14) | 0.74 (0.68, 0.80) |
| Solomon Islands | 21.68 (8.53, 43.18) | 50.68 (19.91, 98.83) | 27.42 (10.91, 53.70) | 28.90 (11.39, 56.03) | 0.06 (-0.04, 0.16) |
| Somalia | 402.47 (146.27, 801.44) | 1022.61 (363.83, 2119.46) | 22.84 (8.29, 45.38) | 19.99 (7.16, 40.98) | -0.36 (-0.47, -0.26) |
| South Africa | 7273.92 (3443.65, 13079.35) | 12160.02 (5938.65, 21466.31) | 70.97 (33.94, 126.37) | 77.87 (37.75, 138.14) | 0.48 (0.42, 0.54) |
| South Sudan | 796.57 (351.65, 1479.73) | 1006.76 (427.67, 1935.39) | 57.37 (25.75, 105.05) | 42.72 (18.55, 81.00) | -0.64 (-0.86, -0.42) |
| Spain | 19596.52 (9562.15, 33801.46) | 23176.53 (11342.36, 40862.91) | 203.00 (99.20, 349.83) | 266.16 (129.24, 475.35) | 0.96 (0.83, 1.09) |
| Sri Lanka | 1513.61 (591.67, 2956.17) | 2708.98 (1160.36, 5070.06) | 31.83 (12.43, 62.00) | 49.45 (21.20, 92.66) | 1.50 (1.43, 1.57) |
| Sudan | 2652.98 (1089.06, 4989.41) | 7897.18 (3486.84, 14579.39) | 53.20 (22.13, 99.05) | 66.71 (29.54, 122.20) | 1.06 (0.96, 1.17) |
| Suriname | 112.68 (52.99, 200.52) | 174.48 (81.23, 310.15) | 109.05 (51.58, 193.45) | 122.41 (57.00, 217.74) | 0.67 (0.58, 0.76) |
| Sweden | 3838.49 (1787.05, 6915.41) | 5249.22 (2505.11, 9440.18) | 195.35 (90.29, 354.78) | 249.59 (117.38, 454.54) | 0.86 (0.74, 0.97) |
| Switzerland | 2549.92 (1222.62, 4503.70) | 3026.44 (1467.34, 5273.74) | 148.06 (70.95, 263.17) | 164.73 (79.14, 291.02) | 0.32 (0.27, 0.37) |
| Syrian Arab Republic | 1893.11 (831.48, 3532.64) | 2570.91 (1116.99, 4868.58) | 64.01 (28.62, 117.44) | 69.06 (30.75, 128.43) | 0.44 (0.32, 0.55) |
| Taiwan (Province of China) | 2710.25 (1158.89, 5049.61) | 3471.75 (1542.94, 6308.98) | 46.95 (19.92, 87.72) | 66.98 (29.40, 123.58) | 1.09 (0.99, 1.19) |
| Tajikistan | 596.65 (249.15, 1135.21) | 1044.45 (436.57, 1957.64) | 44.85 (18.87, 84.70) | 39.36 (16.39, 73.99) | -0.03 (-0.48, 0.42) |
| Thailand | 6311.44 (2613.69, 12094.70) | 8224.85 (3533.39, 15166.04) | 37.67 (15.56, 71.98) | 54.77 (23.42, 101.47) | 1.15 (1.09, 1.20) |
| Timor-Leste | 59.75 (23.76, 117.08) | 147.84 (61.86, 285.54) | 30.11 (11.92, 58.71) | 40.50 (17.09, 77.58) | 1.32 (1.11, 1.52) |
| Togo | 357.67 (151.08, 684.53) | 914.42 (386.75, 1702.97) | 39.32 (16.69, 74.36) | 41.06 (17.36, 76.31) | 0.10 (0.02, 0.19) |
| Tokelau | 0.12 (0.05, 0.25) | 0.13 (0.06, 0.26) | 34.42 (13.29, 67.84) | 42.34 (17.53, 81.32) | 0.79 (0.73, 0.85) |
| Tonga | 7.95 (3.12, 15.68) | 10.08 (4.24, 19.45) | 34.31 (13.72, 66.72) | 39.46 (16.69, 75.70) | 0.39 (0.36, 0.41) |
| Trinidad and Tobago | 393.35 (185.36, 720.53) | 521.94 (246.05, 926.90) | 121.49 (57.19, 222.54) | 162.73 (76.33, 289.69) | 1.47 (1.31, 1.64) |
| Tunisia | 1575.71 (706.64, 2945.64) | 2723.16 (1249.61, 4910.56) | 72.49 (32.74, 134.20) | 92.54 (42.08, 168.03) | 0.87 (0.81, 0.93) |
| Turkey | 15344.20 (7223.97, 27837.21) | 30717.55 (14659.94, 54714.03) | 103.81 (49.39, 186.42) | 146.05 (69.55, 261.46) | 1.58 (1.45, 1.71) |
| Turkmenistan | 498.67 (210.69, 936.85) | 880.61 (387.08, 1615.10) | 51.65 (21.84, 96.25) | 69.44 (30.51, 127.38) | 1.39 (1.05, 1.74) |
| Tuvalu | 0.70 (0.27, 1.34) | 1.03 (0.42, 2.03) | 28.12 (10.91, 54.00) | 34.61 (14.14, 67.77) | 0.51 (0.45, 0.57) |
| Uganda | 1412.35 (565.62, 2757.27) | 4908.41 (2060.02, 9413.58) | 33.90 (13.75, 65.17) | 44.08 (18.68, 83.37) | 0.99(0.94, 1.03) |
| Ukraine | 7263.86 (3155.56, 13554.09) | 5024.98 (2191.63, 9230.25) | 58.23 (25.06, 109.34) | 54.97 (23.70, 102.49) | 0.19 (-0.10, 0.48) |
| United Arab Emirates | 602.38 (286.43, 1089.45) | 2082.86 (1023.46, 3629.66) | 160.91 (76.15, 291.70) | 139.12 (67.56, 247.49) | -0.47 (-0.52, -0.41) |
| United Kingdom | 22478.28 (11338.03, 39333.73) | 28495.93 (14554.33, 48987.09) | 160.09 (80.44, 281.79) | 193.76 (98.00, 336.07) | 0.70 (0.64, 0.77) |
| United Republic of Tanzania | 2516.11 (1002.96, 4872.66) | 7617.04 (3234.95, 14310.54) | 39.11 (15.88, 74.79) | 48.82 (20.94, 90.98) | 0.94 (0.82, 1.07) |
| United States of America | 125030.49 (62158.22, 217886.94) | 128526.48 (64120.15, 226218.58) | 188.11 (92.84, 330.12) | 176.62 (87.59, 312.96) | -0.48 (-0.71, -0.26) |
| United States Virgin Islands | 39.89 (18.78, 71.86) | 29.10 (14.39, 51.27) | 146.56 (69.10, 263.38) | 184.38 (90.90, 325.33) | 0.76 (0.51, 1.01) |
| Uruguay | 935.14 (433.14, 1692.85) | 1265.03 (609.98, 2269.68) | 124.92 (57.89, 226.09) | 157.20 (75.75, 283.06) | 0.69 (0.57, 0.81) |
| Uzbekistan | 2310.47 (981.96, 4413.01) | 4765.09 (2084.19, 8997.81) | 43.16 (18.24, 81.86) | 53.30 (23.12, 101.34) | 0.92 (0.70, 1.15) |
| Vanuatu | 11.21 (4.60, 21.46) | 25.60 (10.50, 49.79) | 30.03 (12.30, 57.19) | 31.55 (12.99, 61.21) | 0.16 (0.13, 0.19) |
| Venezuela | 6056.41 (2839.80, 10850.02) | 7005.55 (3252.03, 12691.66) | 116.85 (54.84, 208.78) | 108.94 (50.51, 197.66) | 0.08 (-0.05, 0.22) |
| Viet Nam | 4612.41 (1754.89, 9044.55) | 10388.20 (4314.45, 19404.27) | 24.88 (9.46, 48.71) | 41.63 (17.08, 78.25) | 1.76 (1.72, 1.79) |
| Yemen | 1652.82 (733.14, 3090.84) | 4879.99 (2111.93, 9010.57) | 56.58(25.22, 104.93) | 55.86 (24.32, 102.71) | 0.30 (0.11, 0.49) |
| Zambia | 869.64 (364.99, 1644.35) | 2780.18 (1190.86, 5130.02) | 44.20 (18.81, 82.34) | 52.99 (22.78, 97.03) | 0.95 (0.75, 1.16) |
| Zimbabwe | 1317.55 (557.56, 2464.66) | 1911.97 (795.54, 3597.30) | 51.19 (21.93, 94.82) | 45.09 (18.91, 84.37) | -0.67 (-0.85, -0.50) |

Abbreviations: DALYs, disability-adjusted life-years; BN, bulimia nervosa; WCBA, women of childbearing age; ASDR, age-standardized DALY rate; EAPC, estimated annual percentage change; UI, uncertainty interval; CI, confidence interval
